# Supplementary material for: Genome-wide association study identifies genetic factors that modify age at onset in Machado-Joseph disease
Source: Aging (Albany NY). 2020 Mar 23;12(6):4742–56. doi: 10.18632/aging.102825 (PMC7138549; doi:10.18632/aging.102825)
Supplement: Supplementary Figures [file aging-12-102825-s006..pdf]

SUPPLEMENTARY FIGURES

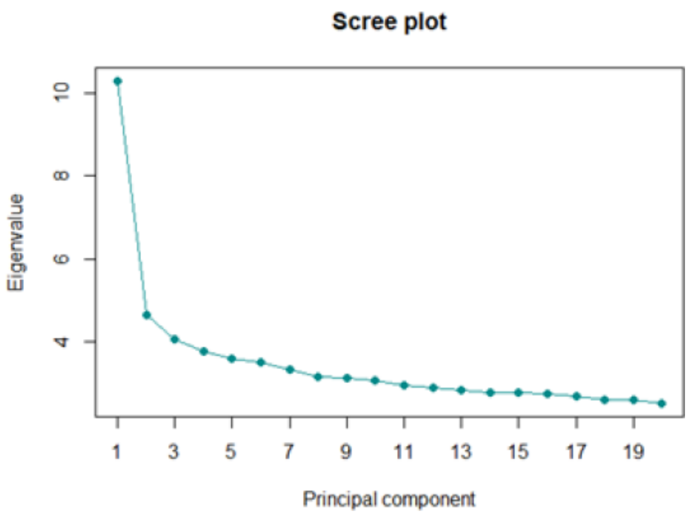

Supplementary Figure 1. Scree plot showing the eigenvalues of the first 20 principal components (PCs). This plot indicates that the first three PCs explain the majority of the variability in data.

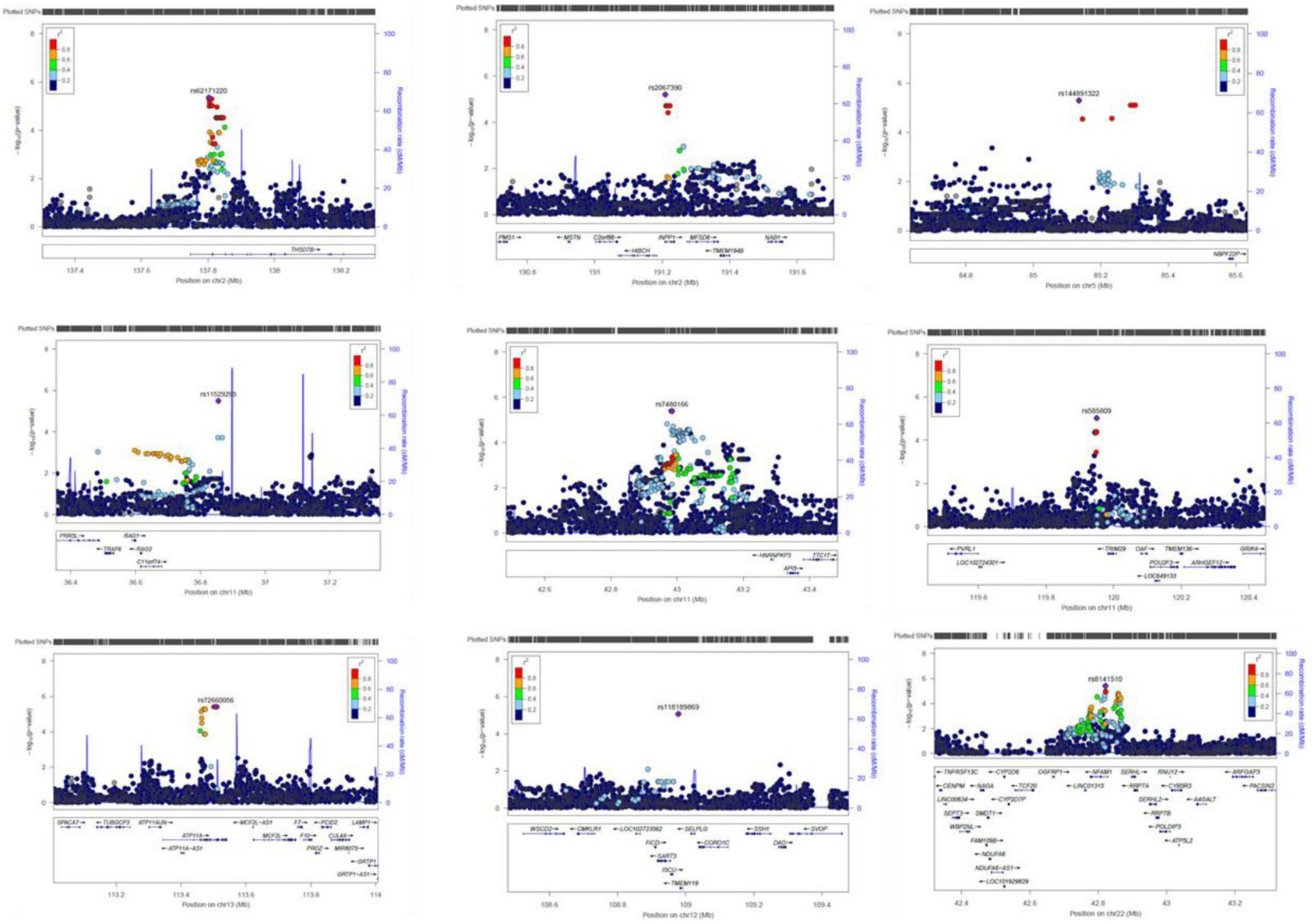

Supplementary Figure 2. Regional LocusZoom plots for the nine modifier loci that modify AO of MJD. Purple line indicates the genetic recombination rate (cM/Mb). SNPs in linkage disequilibrium with identified are shown in color gradient indicating r2 levels (hg19, 1KGP, Nov 2014, EUR).
