# Supplementary material for: Genome-wide association study identifies genetic factors that modify age at onset in Machado-Joseph disease
Source: Aging (Albany NY). 2020 Mar 23;12(6):4742–56. doi: 10.18632/aging.102825 (PMC7138549; doi:10.18632/aging.102825)
Supplement: Supplementary Tables 1, 2, 5 [file aging-12-102825-s005..pdf]

SUPPLEMENTARY TABLES

Supplementary Table 1. Linear relationship between AO and CAG_{exp}, CAG_{nor}, geographical origin, sex and pairwise interaction of the given factors. A total of 62.7 % of the variability in the AO is explained by given factors.

Model description	Multiple R^2	Adjusted R^2	P-value	ΔR^2
AO ~ CAG_{exp}	0.6200	0.6195	$<2.2 \times 10^{-16}$	
AO ~ CAG_{exp} + origin	0.6241	0.6216	$<2.2 \times 10^{-16}$	0.0021
AO ~ CAG_{exp} + origin + sex	0.6265	0.6235	$<2.2 \times 10^{-16}$	0.0019
AO ~ CAG_{exp} + origin + sex + CAG_{nor}	0.6282	0.6247	$<2.2 \times 10^{-16}$	0.0012
AO ~ CAG_{exp} + origin + sex + CAG_{nor} + CAG_{exp}:CAG_{nor}	0.6301	0.6261	$<2.2 \times 10^{-16}$	0.0014
AO ~ CAG_{exp} + origin + sex + CAG_{nor} + CAG_{exp}:CAG_{nor} + CAG_{exp}:origin	0.6328	0.6267	$<2.2 \times 10^{-16}$	0.0006
AO ~ CAG_{exp} + origin + sex + CAG_{nor} + CAG_{exp}:CAG_{nor} + CAG_{exp}:origin + CAG_{nor}:origin	0.6352	0.6271	$<2.2 \times 10^{-16}$	0.0004

Supplementary Table 2. Subjects and cohort demographics.

Geographical origin	# of patients	Mean (SD) AO	M:F
Portugal	330	40.0 (±12.4)	1.0
Brazil	311	34.9 (±11.7)	1.1
North America	55	37.8 (±12.2)	0.7
Germany	51	37.6 (±9.2)	1.2
NA	34	37.1 (±11.1)	1.4
Australia	5	52.8 (±10.1)	0.3

M:F - male-female ratio.

Supplementary Table 5. Gene sets and pathways enriched in i-GSEA4GWAS.

Pathway/Gene set name	Gene Set ID	P-value	FDR
GO: TRANSPORT VESICLE	GO:0030133	0.001	0.0082
KEGG: OLFACTORY TRANSDUCTION	hsa04740	0.001	0.0083
Olfactory Signaling Pathway	R-HSA-381753	0.001	0.0088
GO: SYNAPSE PART	GO:0044456	0.001	0.0093
Synthesis of bile acids and bile salts via 24-hydroxycholesterol	R-HSA-193775	0.001	0.0095
Negative feedback regulation of MAPK pathway	R-HSA-5674499	0.001	0.0101
Purine salvage	R-HSA-74217	0.001	0.0106
GO: METALLOEXOPEPTIDASE ACTIVITY	GO:0008235	0.001	0.0109
GO: SYNAPSE	GO:0045202	0.001	0.0115
GO: MONOCARBOXYLIC ACID TRANSPORT	GO:0015718	0.001	0.0133
GO: OUTER MEMBRANE	GO:0019867	0.001	0.0141
GO: AMINOPEPTIDASE ACTIVITY	GO:0004177	0.001	0.015
Localization of the PINCH-ILK-PARVIN complex to focal adhesions	R-HSA-446343	0.001	0.0159
BioCarta: PLATELETAPP PATHWAY	M6487	0.001	0.0161
GO: EXOPEPTIDASE ACTIVITY	GO:0008238	0.001	0.018
TFAP2 (AP-2) family regulates transcription of other transcription factors	R-HSA-8866906	0.001	0.0212

GO: MITOCHONDRIAL OUTER MEMBRANE	GO:0005741	0.001	0.0215
GO: ORGANELLE OUTER MEMBRANE	GO:0031968	0.003	0.0216
GO: HISTONE DEACETYLASE COMPLEX	GO:0000118	0.001	0.0218
Fanconi Anemia Pathway	R-HSA-6783310	0.001	0.0221
GO: PROTEASOME COMPLEX	GO:0000502	0.001	0.0221
GO: RECEPTOR MEDIATED ENDOCYTOSIS	GO:0006898	0.001	0.0225
GO: GABA RECEPTOR ACTIVITY	GO:0016917	0.001	0.0226
Metabolism of Angiotensinogen to Angiotensins	R-HSA-2022377	0.001	0.0231
GO: TRANS GOLGI NETWORK TRANSPORT VESICLE	GO:0030140	0.001	0.0236
Calcitonin-like ligand receptors	R-HSA-419812	0.001	0.0242
GO: MONOCARBOXYLIC ACID TRANSMEMBRANE TRANSPORTER ACTIVITY	GO:0008028.	0.001	0.0245
HDL remodeling	R-HSA-8964058	0.001	0.0262
Synthesis of epoxy (EET) and dihydroxyeicosatrienoic acids (DHET)	R-HSA-2142670	0.001	0.0263
GO: RESPONSE TO HORMONE STIMULUS	GO:0009725	0.001	0.0273
GO: PEPTIDE METABOLIC PROCESS	GO:0006518	0.004	0.0348
GRB7 events in ERBB2 signaling	R-HSA-1306955	0.001	0.0348
Mitochondrial Uncoupling Proteins	R-HSA-166187	0.008	0.0354
The fatty acid cycling model	R-HSA-167826	0.008	0.0354
The proton buffering model	R-HSA-167827	0.008	0.0354
ATF6 (ATF6-alpha) activates chaperone genes	R-HSA-381183	0.002	0.037
GO: GOLGI ASSOCIATED VESICLE	GO:0005798	0.003	0.0371
Free fatty acids regulate insulin secretion	R-HSA-400451	0.001	0.0374
GO: XENOBIOTIC METABOLIC PROCESS	GO:0006805	0.001	0.0382
GO: DENDRITE	GO:0030425	0.001	0.0386
Beta-oxidation of pristanoyl-CoA	R-HSA-389887	0.001	0.0413
Toxicity of botulinum toxin type D (BoNT/D)	R-HSA-5250955	0.001	0.0414
Toxicity of botulinum toxin type F (BoNT/F)	R-HSA-5250981	0.001	0.0414
Processing and activation of SUMO	R-HSA-3215018	0.001	0.0416
KEGG: NUCLEOTIDE EXCISION REPAIR	hsa03420	0.001	0.0422
GO: RESPONSE TO XENOBIOTIC STIMULUS	GO:0009410	0.001	0.0462
