# Supplementary material for: Genome-wide association study identifies genetic factors that modify age at onset in Machado-Joseph disease
Source: Aging (Albany NY). 2020 Mar 23;12(6):4742–56. doi: 10.18632/aging.102825 (PMC7138549; doi:10.18632/aging.102825)
Supplement: Supplementary Table 3 [file aging-12-102825-s004..docx]

| **Supplementary Table 3.** **Functional annotation of SNPs. rsID:** reference SNP ID; MAF: minor alelle frequency; r^2^: the maximum r^2^ of the SNP with one of the independent significant SNPs; nearest gene: the nearest Gene of the SNP based on ANNOVAR annotations; distance: distance to the nearest gene; func: functional consequence of the SNP on the gene obtained from ANNOVAR; CADD: CADD score which is computed based on 63 annotations. | | | | | | | | | | | | | | |
| --- | --- | --- | --- | --- | --- | --- | --- | --- | --- | --- | --- | --- | --- | --- |
| rsID | chromosome | position | other allele | effect allele | MAF | gwasP | beta | se | r^2^ | lead SNP | nearest gene | distance | func | CADD |
| rs17727536 | 2 | 137815066 | A | G | 0.081 | 0.001781 | 2.34956 | 0.751991 | 0.753951 | rs62171220 | THSD7B | 0 | intronic | 5.562 |
| rs62171225 | 2 | 137809746 | G | A | 0.1097 | 9.47E-06 | 2.68848 | 0.607035 | 0.986517 |  | THSD7B | 0 | intronic | 4.246 |
| rs78384590 | 2 | 137809443 | C | T | 0.1097 | 9.47E-06 | 2.68848 | 0.607035 | 0.986517 |  | THSD7B | 0 | intronic | 2.848 |
| rs62171224 | 2 | 137807239 | A | C | 0.1836 | 0.000312 | 1.85725 | 0.515216 | 0.536419 |  | THSD7B | 0 | intronic | 2.612 |
| rs62170965 | 2 | 137782981 | T | C | 0.09859 | 0.001961 | 1.90845 | 0.616416 | 0.733427 |  | THSD7B | 0 | intronic | 1.687 |
| rs62171246 | 2 | 137833858 | G | A | 0.1103 | 3.08E-05 | 2.5124 | 0.602882 | 0.983984 |  | THSD7B | 0 | intronic | 1.654 |
| rs10928593 | 2 | 137826816 | T | C | 0.1103 | 3.08E-05 | 2.5124 | 0.602882 | 0.983984 |  | THSD7B | 0 | intronic | 13.82 |
| rs62172275 | 2 | 137837083 | G | T | 0.1103 | 3.08E-05 | 2.5124 | 0.602882 | 0.983474 |  | THSD7B | 0 | intronic | 13.03 |
| rs62172274 | 2 | 137837032 | A | G | 0.1103 | 3.08E-05 | 2.5124 | 0.602882 | 0.983474 |  | THSD7B | 0 | intronic | 11.56 |
| rs62171230 | 2 | 137812855 | G | A | 0.1098 | 9.47E-06 | 2.68848 | 0.607035 | 0.986004 |  | THSD7B | 0 | intronic | 8.479 |
| rs62171234 | 2 | 137827198 | C | T | 0.1096 | 1.10E-05 | 2.66813 | 0.606912 | 0.980263 |  | THSD7B | 0 | intronic | 6.488 |
| rs79909386 | 2 | 137824760 | T | C | 0.1102 | 3.08E-05 | 2.5124 | 0.602882 | 0.98498 |  | THSD7B | 0 | intronic | 3.491 |
| rs62172282 | 2 | 137845936 | C | T | 0.1096 | 3.08E-05 | 2.5124 | 0.602882 | 0.98076 |  | THSD7B | 0 | intronic | 3.453 |
| rs11883933 | 2 | 137832705 | T | C | 0.1855 | 0.000124 | 2.01087 | 0.524029 | 0.530676 |  | THSD7B | 0 | intronic | 3.354 |
| rs60034177 | 2 | 137810908 | C | A | 0.1097 | 9.47E-06 | 2.68848 | 0.607035 | 0.986517 |  | THSD7B | 0 | intronic | 2.529 |
| ._3606574 | 2 | 137827244 | C | T | 0.1103 | 3.08E-05 | 2.5124 | 0.602882 | 0.983984 |  | THSD7B | 0 | intronic | 1.901 |
| rs62170966 | 2 | 137788932 | A | C | 0.09854 | 0.002707 | 1.85554 | 0.618682 | 0.733886 |  | THSD7B | 0 | intronic | 1.49 |
| rs62172280 | 2 | 137843858 | G | C | 0.1102 | 3.08E-05 | 2.5124 | 0.602882 | 0.983464 |  | THSD7B | 0 | intronic | 1.121 |
| rs62171244 | 2 | 137833099 | G | A | 0.1103 | 3.08E-05 | 2.5124 | 0.602882 | 0.983984 |  | THSD7B | 0 | intronic | 0.884 |
| rs62171243 | 2 | 137832935 | C | G | 0.1103 | 3.08E-05 | 2.5124 | 0.602882 | 0.983984 |  | THSD7B | 0 | intronic | 0.634 |
| rs4467332 | 2 | 137804803 | G | T | 0.1068 | 5.10E-06 | 2.71272 | 0.594799 | 0.998953 |  | THSD7B | 0 | intronic | 0.561 |
| rs115523037 | 2 | 137846769 | A | G | 0.11 | 3.08E-05 | 2.5124 | 0.602882 | 0.982895 |  | THSD7B | 0 | intronic | 0.512 |
| rs36070202 | 2 | 137850215 | T | C | 0.1871 | 7.67E-05 | 2.04881 | 0.518108 | 0.511144 |  | THSD7B | 0 | intronic | 0.404 |
| rs80284286 | 2 | 137841248 | G | A | 0.1103 | 3.08E-05 | 2.5124 | 0.602882 | 0.983469 |  | THSD7B | 0 | intronic | 14.21 |
| rs62172276 | 2 | 137837088 | A | G | 0.1103 | 3.08E-05 | 2.5124 | 0.602882 | 0.983474 |  | THSD7B | 0 | intronic | 14.1 |
| rs6727323 | 2 | 137814804 | G | A | 0.1102 | 0.000365 | 2.09319 | 0.587313 | 0.984468 |  | THSD7B | 0 | intronic | 13.21 |
| rs60939389 | 2 | 137814138 | T | C | 0.1098 | 0.000205 | 2.14048 | 0.576513 | 0.985491 |  | THSD7B | 0 | exonic | 11.23 |
| rs62171220 | 2 | 137802855 | C | G | 0.1064 | 4.45E-06 | 2.71275 | 0.591089 | 1 |  | THSD7B | 0 | intronic | 8.687 |
| rs62173771 | 2 | 137769773 | T | C | 0.09888 | 0.001961 | 1.90845 | 0.616416 | 0.730574 |  | THSD7B | 0 | intronic | 7.414 |
| rs62170969 | 2 | 137794678 | A | G | 0.0986 | 0.001785 | 1.94279 | 0.621919 | 0.733422 |  | THSD7B | 0 | intronic | 6.814 |
| rs62170961 | 2 | 137775126 | T | C | 0.09866 | 0.001961 | 1.90845 | 0.616416 | 0.73298 |  | THSD7B | 0 | intronic | 6.29 |
| rs62171233 | 2 | 137825276 | T | C | 0.1103 | 3.08E-05 | 2.5124 | 0.602882 | 0.98346 |  | THSD7B | 0 | intronic | 5.976 |
| rs62171221 | 2 | 137803313 | T | A | 0.1065 | 5.10E-06 | 2.71272 | 0.594799 | 0.998954 |  | THSD7B | 0 | intronic | 5.028 |
| rs59631603 | 2 | 137811041 | G | A | 0.1097 | 9.47E-06 | 2.68848 | 0.607035 | 0.986517 |  | THSD7B | 0 | intronic | 4.965 |
| rs13026531 | 2 | 137805397 | G | T | 0.1789 | 0.000122 | 1.99228 | 0.518666 | 0.552163 |  | THSD7B | 0 | intronic | 4.02 |
| rs62171222 | 2 | 137803798 | T | C | 0.1066 | 9.93E-06 | 2.62279 | 0.593554 | 0.998954 |  | THSD7B | 0 | intronic | 3.221 |
| rs62171241 | 2 | 137831155 | T | G | 0.1103 | 3.08E-05 | 2.5124 | 0.602882 | 0.983984 |  | THSD7B | 0 | intronic | 3.07 |
| rs11886749 | 2 | 137834132 | T | C | 0.1855 | 0.000124 | 2.01087 | 0.524029 | 0.530676 |  | THSD7B | 0 | intronic | 2.946 |
| rs62171223 | 2 | 137804104 | A | C | 0.1066 | 5.10E-06 | 2.71272 | 0.594799 | 0.998954 |  | THSD7B | 0 | intronic | 2.696 |
| rs11888486 | 2 | 137811629 | A | G | 0.1097 | 5.10E-06 | 2.71272 | 0.594799 | 0.986517 |  | THSD7B | 0 | intronic | 2.378 |
| rs76046341 | 2 | 137841937 | G | A | 0.1103 | 3.08E-05 | 2.5124 | 0.602882 | 0.983469 |  | THSD7B | 0 | intronic | 2.039 |
| rs62171240 | 2 | 137831031 | C | T | 0.1103 | 3.08E-05 | 2.5124 | 0.602882 | 0.983984 |  | THSD7B | 0 | intronic | 1.863 |
| rs57824544 | 2 | 137775883 | A | G | 0.09858 | 0.001961 | 1.90845 | 0.616416 | 0.733433 |  | THSD7B | 0 | intronic | 1.753 |
| rs62171232 | 2 | 137821243 | C | A | 0.1102 | 0.000365 | 2.09319 | 0.587313 | 0.98498 |  | THSD7B | 0 | intronic | 1.583 |
| rs1030623 | 2 | 137826111 | C | T | 0.1106 | 3.08E-05 | 2.5124 | 0.602882 | 0.980409 |  | THSD7B | 0 | intronic | 1.528 |
| rs9287459 | 2 | 137812019 | G | A | 0.1847 | 0.00016 | 1.92232 | 0.509171 | 0.53225 |  | THSD7B | 0 | intronic | 1.524 |
| rs62171245 | 2 | 137833173 | G | A | 0.1104 | 3.08E-05 | 2.5124 | 0.602882 | 0.983465 |  | THSD7B | 0 | intronic | 1.41 |
| rs143830505 | 2 | 137828757 | A | G | 0.1103 | 3.08E-05 | 2.5124 | 0.602882 | 0.983984 |  | THSD7B | 0 | intronic | 1.392 |
| rs62170967 | 2 | 137791760 | G | A | 0.09859 | 0.001961 | 1.90845 | 0.616416 | 0.733427 |  | THSD7B | 0 | intronic | 1.299 |
| rs72985553 | 2 | 137779070 | A | G | 0.09839 | 0.001699 | 1.92776 | 0.614272 | 0.732736 |  | THSD7B | 0 | intronic | 0.638 |
| rs62171228 | 2 | 137811749 | G | C | 0.1097 | 9.47E-06 | 2.68848 | 0.607035 | 0.986517 |  | THSD7B | 0 | intronic | 0.599 |
| rs62172279 | 2 | 137842028 | T | C | 0.1103 | 3.08E-05 | 2.5124 | 0.602882 | 0.983469 |  | THSD7B | 0 | intronic | 0.371 |
| rs5006938 | 2 | 137804755 | G | A | 0.1092 | 7.07E-06 | 2.66048 | 0.592317 | 0.974923 |  | THSD7B | 0 | intronic | 0.31 |
| rs62171231 | 2 | 137818079 | A | G | 0.1102 | 0.000365 | 2.09319 | 0.587313 | 0.984468 |  | THSD7B | 0 | intronic | 0.111 |
| rs62172281 | 2 | 137843961 | G | A | 0.1102 | 3.08E-05 | 2.5124 | 0.602882 | 0.983464 |  | THSD7B | 0 | intronic | 0.026 |
| rs62170960 | 2 | 137773404 | T | C | 0.09875 | 0.002707 | 1.85554 | 0.618682 | 0.732523 |  | THSD7B | 0 | intronic | 5.691 |
| rs1432235 | 2 | 137805010 | G | T | 0.107 | 5.10E-06 | 2.71272 | 0.594799 | 0.998954 |  | THSD7B | 0 | intronic | 1.026 |
| rs79892386 | 2 | 191217432 | A | G | 0.0374 | 3.90E-05 | 4.79657 | 1.16604 | 0.978409 | rs2067390 | INPP1 | 0 | intronic | 0.287 |
| rs4274625 | 2 | 191215035 | A | G | 0.05503 | 0.023162 | 1.53347 | 0.675312 | 0.663872 |  | INPP1 | 0 | intronic | 5.923 |
| rs79285470 | 2 | 191221777 | G | A | 0.03817 | 1.94E-05 | 4.68752 | 1.09727 | 0.978458 |  | INPP1 | 0 | intronic | 6.664 |
| rs7601609 | 2 | 191219850 | G | T | 0.05609 | 0.028145 | 1.48036 | 0.674343 | 0.654636 |  | INPP1 | 0 | intronic | 6.088 |
| rs79600449 | 2 | 191213313 | T | C | 0.0376 | 1.94E-05 | 4.68752 | 1.09727 | 0.981147 |  | INPP1 | 0 | intronic | 4.285 |
| rs76928748 | 2 | 191210862 | T | C | 0.03755 | 1.94E-05 | 4.68752 | 1.09727 | 0.982497 |  | INPP1 | 0 | intronic | 3.699 |
| rs140133024 | 2 | 191223051 | C | G | 0.03817 | 1.94E-05 | 4.68752 | 1.09727 | 0.978458 |  | INPP1 | 0 | intronic | 1.087 |
| rs139703012 | 2 | 191214728 | G | A | 0.0376 | 1.94E-05 | 4.68752 | 1.09727 | 0.981147 |  | INPP1 | 0 | intronic | 0.27 |
| rs2067390 | 2 | 191209028 | T | A | 0.0382 | 6.39E-06 | 4.74017 | 1.05033 | 1 |  | INPP1 | 0 | intronic | 2.174 |
| rs144891322 | 5 | 85135387 | T | C | 0.0214 | 5.18E-06 | 6.09914 | 1.33831 | 1 | rs144891322 | CTD-2036A18.2 | 8932 | intergenic | 1.341 |
| rs116703166 | 5 | 85295591 | A | G | 0.02318 | 8.16E-06 | 6.16024 | 1.38097 | 0.947369 |  | PTP4A1P4 | 87752 | intergenic | 8.74 |
| rs115318856 | 5 | 85232893 | A | G | 0.02237 | 2.80E-05 | 5.87828 | 1.40331 | 0.939005 |  | CTD-2036A18.2 | 87875 | intergenic | 4.988 |
| rs148861235 | 5 | 85302229 | A | G | 0.02318 | 8.16E-06 | 6.16024 | 1.38097 | 0.947369 |  | PTP4A1P4 | 81114 | intergenic | 2.138 |
| rs146233327 | 5 | 85288187 | G | C | 0.02318 | 8.16E-06 | 6.16024 | 1.38097 | 0.947369 |  | PTP4A1P4 | 95156 | intergenic | 2.872 |
| rs1604087 | 5 | 85146176 | G | A | 0.02206 | 2.98E-05 | 5.10516 | 1.22277 | 0.981201 |  | CTD-2036A18.2 | 1158 | intergenic | 0.142 |
| rs12418749 | 11 | 36706549 | A | G | 0.1035 | 0.002135 | -1.92513 | 0.626907 | 0.603719 | rs11529293 | CTD-2119L1.1 | 11317 | intergenic | 14.29 |
| ._18710870 | 11 | 36612700 | T | C | 0.1027 | 0.000614 | -2.12088 | 0.619156 | 0.596652 |  | RAG1:RAG2 | 0:00 | intronic | 7.177 |
| rs7949234 | 11 | 36741502 | C | T | 0.1086 | 0.00264 | -1.85735 | 0.617708 | 0.691852 |  | CTD-2119L1.1 | 22084 | intergenic | 1.791 |
| rs2227973 | 11 | 36597313 | A | G | 0.1048 | 0.000806 | -2.10776 | 0.629075 | 0.581039 |  | RAG1:RAG2 | 0:00 | exonic | 17.3 |
| rs10836582 | 11 | 36655751 | T | C | 0.1036 | 0.001152 | -1.92099 | 0.591005 | 0.595166 |  | C11orf74 | 0 | intronic | 5.221 |
| ._18712253 | 11 | 36741120 | C | T | 0.1087 | 0.002955 | -1.7724 | 0.59629 | 0.690944 |  | CTD-2119L1.1 | 21702 | intergenic | 4.623 |
| rs12293560 | 11 | 36701581 | C | T | 0.1022 | 0.001356 | -2.00771 | 0.626645 | 0.610784 |  | C11orf74 | 6757 | intergenic | 4.185 |
| rs12419177 | 11 | 36677521 | A | T | 0.1035 | 0.001158 | -2.0481 | 0.630351 | 0.596068 |  | C11orf74 | 0 | intronic | 2.614 |
| rs10734444 | 11 | 36743826 | C | T | 0.1072 | 0.00264 | -1.85735 | 0.617708 | 0.708096 |  | CTD-2119L1.1 | 24408 | intergenic | 1.472 |
| rs79952749 | 11 | 36698773 | G | T | 0.1025 | 0.001586 | -1.84912 | 0.585438 | 0.609634 |  | C11orf74 | 3949 | intergenic | 0.977 |
| rs7119197 | 11 | 36607768 | A | G | 0.1044 | 0.000975 | -1.87338 | 0.568081 | 0.587107 |  | RAG1:RAG2 | 0:00 | intronic | 0.669 |
| rs6484872 | 11 | 36759190 | T | A | 0.1065 | 0.02168 | -1.29645 | 0.564676 | 0.727068 |  | CTD-2119L1.1 | 39772 | intergenic | 0.198 |
| rs59563638 | 11 | 36692302 | G | A | 0.1022 | 0.001528 | -1.98899 | 0.627589 | 0.608576 |  | C11orf74 | 0 | intronic | 0.15 |
| rs4325287 | 11 | 36729657 | C | T | 0.1051 | 0.001743 | -1.94167 | 0.620165 | 0.649284 |  | CTD-2119L1.1 | 10239 | intergenic | 0.013 |
| rs3758874 | 11 | 36655089 | A | G | 0.1035 | 0.001158 | -2.0481 | 0.630351 | 0.595522 |  | C11orf74 | 0 | intronic | 4.238 |
| rs4256950 | 11 | 36752925 | G | A | 0.1073 | 0.002302 | -1.88038 | 0.616879 | 0.706302 |  | CTD-2119L1.1 | 33507 | intergenic | 3.981 |
| rs6484873 | 11 | 36759211 | A | T | 0.1065 | 0.02168 | -1.29645 | 0.564676 | 0.726991 |  | CTD-2119L1.1 | 39793 | intergenic | 2.572 |
| rs10400354 | 11 | 36663831 | C | G | 0.1037 | 0.001152 | -1.92099 | 0.591005 | 0.594794 |  | C11orf74 | 0 | intronic | 1.849 |
| rs11529293 | 11 | 36855388 | C | T | 0.1184 | 3.30E-06 | -2.71476 | 0.583662 | 1 |  | CTD-2119L1.1 | 135970 | intergenic | 1.318 |
| rs71481983 | 11 | 36672675 | C | T | 0.09821 | 0.001532 | -2.01917 | 0.637268 | 0.577769 |  | C11orf74 | 0 | intronic | 1.289 |
| rs16929258 | 11 | 36667581 | C | T | 0.1022 | 0.001158 | -2.0481 | 0.630351 | 0.603422 |  | C11orf74 | 0 | intronic | 0.739 |
| rs66816307 | 11 | 36710511 | T | C | 0.1028 | 0.001668 | -1.95022 | 0.620336 | 0.610684 |  | CTD-2119L1.1 | 7355 | intergenic | 0.617 |
| rs1498336 | 11 | 36639684 | G | T | 0.1022 | 0.001158 | -2.0481 | 0.630351 | 0.603388 |  | C11orf74 | 0 | intronic | 3.135 |
| rs1498338 | 11 | 36632383 | G | A | 0.1023 | 0.001158 | -2.0481 | 0.630351 | 0.603371 |  | C11orf74 | 0 | intronic | 0.577 |
| rs12225762 | 11 | 43032022 | A | T | 0.2984 | 0.001509 | -1.37864 | 0.434494 | 0.570816 | rs7480166 | RP11-111A24.2 | 53164 | intergenic | 4.187 |
| rs10837979 | 11 | 42973253 | G | A | 0.2928 | 0.001257 | -1.44197 | 0.447024 | 0.619144 |  | CTD-2537L20.1 | 11704 | intergenic | 0.55 |
| rs975104 | 11 | 42992520 | T | C | 0.2957 | 0.000395 | -1.58152 | 0.446321 | 0.596201 |  | CTD-2537L20.1 | 30971 | intergenic | 15.02 |
| rs10837982 | 11 | 42977361 | G | A | 0.3335 | 0.000872 | -1.43392 | 0.430736 | 0.74389 |  | CTD-2537L20.1 | 15812 | intergenic | 13.02 |
| rs10837988 | 11 | 42991421 | C | A | 0.293 | 0.001632 | -1.40905 | 0.447302 | 0.612735 |  | CTD-2537L20.1 | 29872 | intergenic | 6.241 |
| rs1906749 | 11 | 43028917 | A | G | 0.2966 | 0.001814 | -1.359 | 0.435712 | 0.578893 |  | RP11-111A24.2 | 56269 | intergenic | 3.029 |
| rs1565254 | 11 | 42991552 | G | C | 0.2924 | 0.000643 | -1.52016 | 0.445409 | 0.617819 |  | CTD-2537L20.1 | 30003 | intergenic | 2.846 |
| rs10837983 | 11 | 42981653 | C | T | 0.4035 | 4.17E-06 | -1.86279 | 0.404692 | 1 |  | CTD-2537L20.1 | 20104 | intergenic | 2.234 |
| rs10837987 | 11 | 42987138 | C | T | 0.3331 | 0.000545 | -1.49005 | 0.430974 | 0.74352 |  | CTD-2537L20.1 | 25589 | intergenic | 1.63 |
| rs4755680 | 11 | 42971480 | G | A | 0.334 | 0.000872 | -1.43392 | 0.430736 | 0.741595 |  | CTD-2537L20.1 | 9931 | intergenic | 1.291 |
| ._18786105 | 11 | 43031271 | A | G | 0.2985 | 0.001509 | -1.37864 | 0.434494 | 0.570816 |  | RP11-111A24.2 | 53915 | intergenic | 0.021 |
| rs11037192 | 11 | 42996562 | A | C | 0.2956 | 0.000887 | -1.48905 | 0.447967 | 0.596528 |  | CTD-2537L20.1 | 35013 | intergenic | 14.46 |
| rs1000540 | 11 | 42992999 | A | T | 0.2958 | 0.000395 | -1.58152 | 0.446321 | 0.596885 |  | CTD-2537L20.1 | 31450 | intergenic | 9.696 |
| rs11037183 | 11 | 42976450 | G | A | 0.3337 | 0.000872 | -1.43392 | 0.430736 | 0.743515 |  | CTD-2537L20.1 | 14901 | intergenic | 8.873 |
| rs10837984 | 11 | 42984436 | C | G | 0.2932 | 0.001402 | -1.42834 | 0.447161 | 0.620126 |  | CTD-2537L20.1 | 22887 | intergenic | 6.917 |
| rs7949008 | 11 | 43004101 | A | T | 0.2969 | 0.000533 | -1.51679 | 0.437904 | 0.58089 |  | CTD-2537L20.1 | 42552 | intergenic | 5.9 |
| rs7104402 | 11 | 43040988 | C | T | 0.2986 | 0.001509 | -1.37864 | 0.434494 | 0.569967 |  | RP11-111A24.2 | 44198 | intergenic | 5.887 |
| rs111164110 | 11 | 43029956 | T | C | 0.297 | 0.001967 | -1.35339 | 0.437265 | 0.578154 |  | RP11-111A24.2 | 55230 | intergenic | 4.998 |
| rs11602286 | 11 | 42967946 | T | C | 0.3348 | 0.00101 | -1.41598 | 0.430694 | 0.738794 |  | CTD-2537L20.1 | 6397 | intergenic | 4.936 |
| rs12364035 | 11 | 42986886 | C | T | 0.2934 | 0.00067 | -1.51552 | 0.445523 | 0.619353 |  | CTD-2537L20.1 | 25337 | intergenic | 3.443 |
| rs7480166 | 11 | 42984753 | G | A | 0.4038 | 4.17E-06 | -1.86279 | 0.404692 | 1 |  | CTD-2537L20.1 | 23204 | intergenic | 3.381 |
| rs4755681 | 11 | 42973423 | T | A | 0.3338 | 0.000872 | -1.43392 | 0.430736 | 0.742406 |  | CTD-2537L20.1 | 11874 | intergenic | 2.978 |
| rs10837986 | 11 | 42987123 | T | C | 0.333 | 0.000545 | -1.49005 | 0.430974 | 0.743391 |  | CTD-2537L20.1 | 25574 | intergenic | 2.737 |
| rs10837991 | 11 | 43019991 | C | T | 0.296 | 0.002474 | -1.31842 | 0.435623 | 0.576967 |  | CTD-2537L20.1 | 58442 | intergenic | 2.551 |
| rs2087362 | 11 | 42972439 | A | C | 0.3341 | 0.000872 | -1.43392 | 0.430736 | 0.741672 |  | CTD-2537L20.1 | 10890 | intergenic | 2.449 |
| rs4423173 | 11 | 42979924 | T | C | 0.3337 | 0.000872 | -1.43392 | 0.430736 | 0.744237 |  | CTD-2537L20.1 | 18375 | intergenic | 2.191 |
| rs11037213 | 11 | 43025565 | G | A | 0.2963 | 0.002474 | -1.31842 | 0.435623 | 0.577513 |  | RP11-111A24.2 | 59621 | intergenic | 1.501 |
| rs7128956 | 11 | 43051938 | C | T | 0.3138 | 0.006885 | -1.149 | 0.425186 | 0.613288 |  | RP11-111A24.2 | 33248 | intergenic | 1.4 |
| rs4755685 | 11 | 42995896 | C | G | 0.2956 | 0.000351 | -1.59546 | 0.446367 | 0.597186 |  | CTD-2537L20.1 | 34347 | intergenic | 1.079 |
| rs10838025 | 11 | 43161270 | T | G | 0.3645 | 0.000592 | -1.42135 | 0.413746 | 0.500149 |  | HNRNPKP3 | 0 | ncRNA_intronic | 1.004 |
| ._18785128 | 11 | 42962038 | C | T | 0.2934 | 0.001122 | -1.45114 | 0.445412 | 0.618702 |  | CTD-2537L20.1 | 489 | downstream | 0.949 |
| rs7106753 | 11 | 42971631 | G | C | 0.3339 | 0.000872 | -1.43392 | 0.430736 | 0.742406 |  | CTD-2537L20.1 | 10082 | intergenic | 0.761 |
| rs11530111 | 11 | 42965314 | A | C | 0.334 | 0.00101 | -1.41598 | 0.430694 | 0.741826 |  | CTD-2537L20.1 | 3765 | intergenic | 0.749 |
| rs4317973 | 11 | 42962984 | A | G | 0.2934 | 0.001449 | -1.41834 | 0.445364 | 0.618495 |  | CTD-2537L20.1 | 1435 | intergenic | 0.683 |
| rs975105 | 11 | 42992557 | T | A | 0.2959 | 0.000375 | -1.58729 | 0.446211 | 0.595789 |  | CTD-2537L20.1 | 31008 | intergenic | 0.627 |
| rs10768905 | 11 | 42984717 | C | T | 0.4037 | 4.17E-06 | -1.86279 | 0.404692 | 1 |  | CTD-2537L20.1 | 23168 | intergenic | 0.626 |
| rs7105872 | 11 | 43035914 | G | T | 0.2986 | 0.001509 | -1.37864 | 0.434494 | 0.570176 |  | RP11-111A24.2 | 49272 | intergenic | 0.626 |
| rs10837973 | 11 | 42962003 | G | T | 0.334 | 0.00101 | -1.41598 | 0.430694 | 0.741826 |  | CTD-2537L20.1 | 454 | downstream | 0.598 |
| rs1489208 | 11 | 42987523 | C | T | 0.334 | 0.00052 | -1.496 | 0.431098 | 0.743369 |  | CTD-2537L20.1 | 25974 | intergenic | 0.376 |
| rs11037188 | 11 | 42988865 | G | C | 0.2933 | 0.000643 | -1.52016 | 0.445409 | 0.619022 |  | CTD-2537L20.1 | 27316 | intergenic | 0.304 |
| rs7121741 | 11 | 42980015 | C | T | 0.4296 | 0.043282 | 0.803482 | 0.397571 | 0.509736 |  | CTD-2537L20.1 | 18466 | intergenic | 0.273 |
| rs7945187 | 11 | 43003213 | C | T | 0.2969 | 0.001646 | -1.3661 | 0.433997 | 0.580733 |  | CTD-2537L20.1 | 41664 | intergenic | 0.251 |
| rs11037177 | 11 | 42971739 | C | T | 0.2933 | 0.001123 | -1.45102 | 0.445404 | 0.619929 |  | CTD-2537L20.1 | 10190 | intergenic | 0.025 |
| rs10837985 | 11 | 42984474 | G | A | 0.3341 | 0.000462 | -1.5048 | 0.429742 | 0.743604 |  | CTD-2537L20.1 | 22925 | intergenic | 0.005 |
| rs1489204 | 11 | 42960784 | T | C | 0.3339 | 0.00101 | -1.41598 | 0.430694 | 0.74184 |  | CTD-2537L20.1 | 540 | upstream | 6.912 |
| rs10837989 | 11 | 42999477 | A | T | 0.296 | 0.000351 | -1.59546 | 0.446367 | 0.59574 |  | CTD-2537L20.1 | 37928 | intergenic | 6.351 |
| rs2046769 | 11 | 42968875 | T | C | 0.3344 | 0.00101 | -1.41598 | 0.430694 | 0.740537 |  | CTD-2537L20.1 | 7326 | intergenic | 3.863 |
| rs10837990 | 11 | 43005862 | C | G | 0.2967 | 0.001344 | -1.40635 | 0.438602 | 0.580464 |  | CTD-2537L20.1 | 44313 | intergenic | 0.605 |
| rs1541751 | 11 | 43051915 | G | A | 0.3133 | 0.003751 | -1.23329 | 0.425505 | 0.613893 |  | RP11-111A24.2 | 33271 | intergenic | 0.074 |
| rs661621 | 11 | 119944195 | A | T | 0.07152 | 4.41E-05 | -3.75532 | 0.919278 | 0.869437 | rs585809 | TRIM29 | 37787 | intergenic | 0.937 |
| rs648067 | 11 | 119949927 | C | A | 0.08102 | 4.35E-05 | -3.58905 | 0.877964 | 1 |  | TRIM29 | 32055 | intergenic | 18.87 |
| rs597124 | 11 | 119949821 | C | T | 0.08107 | 4.35E-05 | -3.58905 | 0.877964 | 1 |  | TRIM29 | 32161 | intergenic | 18.88 |
| rs649921 | 11 | 119949498 | G | T | 0.08118 | 4.35E-05 | -3.58905 | 0.877964 | 1 |  | TRIM29 | 32484 | intergenic | 7.236 |
| rs677409 | 11 | 119946763 | T | G | 0.08195 | 4.22E-05 | -3.55683 | 0.868611 | 0.993327 |  | TRIM29 | 35219 | intergenic | 3.058 |
| rs662549 | 11 | 119947801 | T | C | 0.08139 | 4.35E-05 | -3.58905 | 0.877964 | 0.999331 |  | TRIM29 | 34181 | intergenic | 2.164 |
| rs1287581 | 11 | 119948738 | G | T | 0.08114 | 0.000366 | -3.19951 | 0.897889 | 1 |  | TRIM29 | 33244 | intergenic | 1.273 |
| rs584282 | 11 | 119945841 | T | G | 0.08143 | 4.22E-05 | -3.55683 | 0.868611 | 0.996647 |  | TRIM29 | 36141 | intergenic | 0.851 |
| rs600720 | 11 | 119948997 | C | T | 0.08115 | 4.35E-05 | -3.58905 | 0.877964 | 1 |  | TRIM29 | 32985 | intergenic | 0.421 |
| rs585809 | 11 | 119949979 | C | T | 0.08108 | 9.50E-06 | -3.75699 | 0.848426 | 1 |  | TRIM29 | 32003 | intergenic | 0.328 |
| rs644324 | 11 | 119946171 | A | G | 0.08121 | 4.83E-05 | -3.58652 | 0.88259 | 0.997986 |  | TRIM29 | 35811 | intergenic | 0.19 |
| rs78743559 | 13 | 113469449 | C | T | 0.06653 | 5.42E-06 | -3.14032 | 0.690518 | 0.792104 | rs72660056 | ATP11A | 0 | intronic | 0.418 |
| rs13378238 | 13 | 113462408 | C | T | 0.06743 | 7.39E-06 | -2.96007 | 0.660406 | 0.767102 |  | ATP11A | 0 | intronic | 4.316 |
| rs75823447 | 13 | 113463922 | G | T | 0.06523 | 3.35E-05 | -2.77935 | 0.669963 | 0.789078 |  | ATP11A | 0 | intronic | 3.218 |
| rs9603955 | 13 | 113463817 | G | A | 0.06636 | 1.69E-05 | -2.90987 | 0.676262 | 0.783795 |  | ATP11A | 0 | intronic | 2.479 |
| rs72660055 | 13 | 113501384 | G | C | 0.06744 | 3.94E-06 | -3.29105 | 0.713186 | 0.998444 |  | ATP11A | 0 | intronic | 1.791 |
| rs72660059 | 13 | 113510723 | A | G | 0.0686 | 3.94E-06 | -3.29105 | 0.713186 | 0.996168 |  | ATP11A | 0 | intronic | 0.832 |
| rs76887510 | 13 | 113475218 | T | C | 0.07005 | 5.42E-06 | -3.14032 | 0.690518 | 0.778294 |  | ATP11A | 0 | intronic | 0.739 |
| rs9603957 | 13 | 113474348 | C | T | 0.06692 | 0.000134 | -2.5514 | 0.668168 | 0.793054 |  | ATP11A | 0 | intronic | 0.321 |
| rs77255514 | 13 | 113473991 | C | T | 0.06678 | 5.42E-06 | -3.14032 | 0.690518 | 0.793729 |  | ATP11A | 0 | intronic | 0.172 |
| rs72660056 | 13 | 113507543 | G | A | 0.06823 | 3.94E-06 | -3.29105 | 0.713186 | 1 |  | ATP11A | 0 | intronic | 0.009 |
| rs75900412 | 13 | 113472469 | G | A | 0.06666 | 0.000134 | -2.5514 | 0.668168 | 0.790814 |  | ATP11A | 0 | intronic | 0.064 |
| ._23795223 | 15 | 99690019 | C | T | 0.06937 | 0.000261 | -3.29259 | 0.901669 | 0.588778 | rs11857349 | TTC23 | 0 | intronic | 1.775 |
| rs11857349 | 15 | 99924857 | A | G | 0.05841 | 3.43E-06 | -4.57624 | 0.985529 | 1 |  | LRRC28 | 0 | intronic | 4.58 |
| ._23795041 | 15 | 99673504 | T | C | 0.0646 | 3.01E-05 | -3.97964 | 0.953799 | 0.655377 |  | SYNM | 0 | UTR3 | 0.595 |
| rs111349178 | 15 | 99913319 | C | T | 0.05834 | 3.43E-06 | -4.57624 | 0.985529 | 0.997197 |  | LRRC28 | 0 | intronic | 0.258 |
| rs112256573 | 15 | 99810484 | A | C | 0.06772 | 0.000425 | -3.26676 | 0.927043 | 0.878347 |  | LRRC28 | 0 | intronic | 0.239 |
| rs78117606 | 15 | 99800366 | G | T | 0.06785 | 0.000425 | -3.26676 | 0.927043 | 0.876242 |  | LRRC28 | 0 | intronic | 5.613 |
| rs76522936 | 15 | 99870062 | T | C | 0.06184 | 0.000265 | -3.47012 | 0.951438 | 0.892263 |  | LRRC28 | 0 | intronic | 1.878 |
| rs7288411 | 22 | 42815358 | G | A | 0.3698 | 0.000348 | 1.45257 | 0.406117 | 0.725832 | rs8141510 | NFAM1 | 0 | intronic | 13.22 |
| rs11090093 | 22 | 42777716 | G | A | 0.4474 | 0.000901 | 1.31056 | 0.394786 | 0.679946 |  | NFAM1 | 0 | UTR3 | 6.012 |
| rs7289512 | 22 | 42796914 | G | A | 0.4786 | 2.93E-05 | -1.61639 | 0.386814 | 0.516802 |  | NFAM1 | 0 | intronic | 4.44 |
| ._28078176 | 22 | 42777966 | C | T | 0.4476 | 0.000901 | 1.31056 | 0.394786 | 0.681397 |  | NFAM1 | 0 | UTR3 | 4.258 |
| rs2899356 | 22 | 42853011 | G | A | 0.3939 | 7.37E-05 | 1.57808 | 0.398093 | 0.794162 |  | CTA-126B4.7 | 10809 | intergenic | 3.608 |
| rs8139123 | 22 | 42822283 | T | C | 0.4258 | 1.19E-05 | 1.73164 | 0.395383 | 0.998366 |  | NFAM1 | 0 | intronic | 3.381 |
| rs11090092 | 22 | 42777694 | A | G | 0.4482 | 0.000901 | 1.31056 | 0.394786 | 0.681355 |  | NFAM1 | 0 | UTR3 | 3.308 |
| rs5751277 | 22 | 42858713 | A | G | 0.3943 | 2.26E-05 | 1.7111 | 0.403764 | 0.781791 |  | CTA-126B4.7 | 16511 | intergenic | 1.996 |
| rs8141510 | 22 | 42821185 | C | T | 0.4254 | 3.94E-06 | 1.82703 | 0.395925 | 1 |  | NFAM1 | 0 | intronic | 1.67 |
| rs5758736 | 22 | 42862437 | C | A | 0.3944 | 5.39E-05 | 1.61253 | 0.39935 | 0.784659 |  | CTA-126B4.7 | 20235 | intergenic | 1.554 |
| rs11090094 | 22 | 42777935 | A | C | 0.4481 | 0.000901 | 1.31056 | 0.394786 | 0.682345 |  | NFAM1 | 0 | UTR3 | 1.057 |
| rs11090090 | 22 | 42777621 | A | G | 0.4718 | 0.000316 | 1.41021 | 0.39156 | 0.611119 |  | NFAM1 | 0 | UTR3 | 0.969 |
| ._28078177 | 22 | 42778078 | A | G | 0.4481 | 0.000797 | 1.31974 | 0.393514 | 0.683044 |  | NFAM1 | 0 | UTR3 | 0.916 |
| rs11090091 | 22 | 42777662 | C | T | 0.448 | 0.000901 | 1.31056 | 0.394786 | 0.681843 |  | NFAM1 | 0 | UTR3 | 0.633 |
| rs8141826 | 22 | 42821501 | A | G | 0.4259 | 1.15E-05 | 1.7288 | 0.394035 | 0.996729 |  | NFAM1 | 0 | intronic | 0.537 |
| rs10854752 | 22 | 42777866 | T | C | 0.448 | 0.000901 | 1.31056 | 0.394786 | 0.682535 |  | NFAM1 | 0 | UTR3 | 0.412 |
| rs2092175 | 22 | 42779752 | T | A | 0.438 | 0.000212 | 1.46022 | 0.394253 | 0.677343 |  | NFAM1 | 0 | UTR3 | 0.35 |
| rs4822133 | 22 | 42866427 | T | G | 0.3826 | 4.16E-05 | 1.645 | 0.401377 | 0.75658 |  | CTA-126B4.7 | 24225 | intergenic | 12.98 |
| rs9611801 | 22 | 42859935 | A | G | 0.3943 | 2.26E-05 | 1.7111 | 0.403764 | 0.782708 |  | CTA-126B4.7 | 17733 | intergenic | 8.628 |
| rs5758735 | 22 | 42862004 | T | C | 0.3945 | 5.39E-05 | 1.61253 | 0.39935 | 0.785025 |  | CTA-126B4.7 | 19802 | intergenic | 3.409 |
| rs2413690 | 22 | 42820264 | A | G | 0.3511 | 0.004183 | 1.18203 | 0.412714 | 0.695247 |  | NFAM1 | 0 | intronic | 2.914 |
| rs2092177 | 22 | 42820250 | C | T | 0.3704 | 0.000388 | 1.48477 | 0.41843 | 0.750008 |  | NFAM1 | 0 | intronic | 0.86 |
| rs5758732 | 22 | 42857285 | A | G | 0.3937 | 5.39E-05 | 1.61253 | 0.39935 | 0.788623 |  | CTA-126B4.7 | 15083 | intergenic | 8.147 |
| rs6002715 | 22 | 42778325 | A | G | 0.4522 | 0.001038 | 1.28947 | 0.393125 | 0.696599 |  | NFAM1 | 0 | UTR3 | 4.667 |
| rs6002713 | 22 | 42778116 | C | T | 0.448 | 0.000797 | 1.31974 | 0.393514 | 0.68292 |  | NFAM1 | 0 | UTR3 | 3.327 |
| rs5758734 | 22 | 42861656 | C | A | 0.3946 | 5.39E-05 | 1.61253 | 0.39935 | 0.78451 |  | CTA-126B4.7 | 19454 | intergenic | 1.563 |
| rs6002714 | 22 | 42778210 | T | C | 0.4158 | 0.001464 | 1.25069 | 0.393096 | 0.575715 |  | NFAM1 | 0 | UTR3 | 0.856 |
| rs5758733 | 22 | 42860486 | A | G | 0.3945 | 2.26E-05 | 1.7111 | 0.403764 | 0.784245 |  | CTA-126B4.7 | 18284 | intergenic | 0.111 |
| rs2899357 | 22 | 42858610 | A | G | 0.3935 | 1.62E-05 | 1.7369 | 0.402865 | 0.785349 |  | CTA-126B4.7 | 16408 | intergenic | 2.234 |
| rs6519319 | 22 | 42815125 | G | A | 0.3198 | 0.007339 | 1.15354 | 0.43026 | 0.586134 |  | NFAM1 | 0 | intronic | 0.688 |
