# Supplementary material for: Genome-wide association study identifies genetic factors that modify age at onset in Machado-Joseph disease
Source: Aging (Albany NY). 2020 Mar 23;12(6):4742–56. doi: 10.18632/aging.102825 (PMC7138549; doi:10.18632/aging.102825)
Supplement: Supplementary Table 4 [file aging-12-102825-s002..docx]

**Supplementary Table 4.** **Previously identified HD-AO modifier loci in MJD.**

| Genetic Modifiers of Huntington’s Disease (GeM-HD) Consortium, 2015 | | | | | | | | | GWAS for MJD-AO modifiers | | |
| --- | --- | --- | --- | --- | --- | --- | --- | --- | --- | --- | --- |
| Locus # | SNP | Chr | Position | Effect Allele | Other Allele | MAF in European HD (%) | Effect Size | p-value | Frequency (%) | Effect size | p-value |
| 1 | rs147804330 | 2 | 56391203 | A | G | 6.3 | -1.6 | 7.6 × 10^-7^ | NA | NA | NA |
| 2 | rs72810940 | 2 | 75555265 | A | G | 2.9 | 2.4 | 5.9 × 10^-7^ | 2.2 | 1.44 | 0.3 |
| 3 | rs144287831 | 3 | 37068079 | C | T | 31.2 | 0.9 | 2.2 × 10^-7^ | 30.0 | -0.98 | 0.02 |
| 4 | rs11133929 | 5 | 2155168 | C | T | 9.3 | 1.5 | 2.1 × 10^-7^ | 9.8 | -0.44 | 0.52 |
| 5 | rs1037699 | 8 | 103250930 | T | C | 9.6 | -1.6 | 2.7 × 10^-8^ | 8.1 | -0.48 | 0.53 |
| 6 | rs11061229 | 12 | 131389783 | C | G | 6.6 | -1.7 | 6.7 × 10^-7^ | 5.4 | -0.54 | 0.56 |
| 7 | rs261453 | 13 | 82324504 | A | C | 11.4 | -1.3 | 9 × 10^-7^ | NA | NA | NA |
| 8 | rs148491145 | 14 | 72360176-72360182 | — | GACTCTA | 1.5 | -3.2 | 7.5 × 10^-7^ | NA | NA | NA |
| 9 | rs146353869 | 15 | 31126401 | A | C | 1.7 | -6.1 | 4.3 × 10^-20^ | 2.1 | -1.28 | 0.37 |
| 10 | rs2140734 | 15 | 31243792 | G | T | 30.4 | 1.4 | 7.1 × 10^-14^ | 2.8 | 0.27 | 0.53 |
| 11 | rs143367341 | 21 | 28348433 | G | A | 13.5 | 1.3 | 2.5 × 10^-8^ | NA | NA | NA |
|  |  |  |  |  |  |  |  |  |  |  |  |
| Genetic Modifiers of Huntington’s Disease (GeM-HD) Consortium, 2019* | | | | | | | | | GWAS for MJD-AO modifiers | | |
| Locus # | Chr | SNP | Position | Effect Allele | Other Allele | MAF in European HD (%) | Effect Size | p-value | Frequency (%) | Effect size | p-value |
| 12 | 2 | rs3791767 | 190639915 | A | C | 20.7 | -0.8 | 6.3 × 10^-8^ | 36.1 | -0.26 | 0.57 |
| 3 | 3 | rs1799977 | 37053568 | G | A | 31.0 | 0.8 | 5.1 × 10^-10^ | 30 | -0.98 | 0.02 |
| 13 | 5 | rs701383 | 79913275 | A | G | 25.7 | -0.8 | 2.5 × 10^-8^ | 22.6 | -0.32 | 0.51 |
|  | 5 | rs11336158 | 80086504 | G | A | 0.3 | 6.1 | 1.3 × 10^-9^ | NA | NA | NA |
|  | 5 | rs1650742 | 79990883 | G | T | 33.1 | 0.6 | 1.6 × 10^-6^ | 35 | 0.24 | 0.57 |
| 14 | 5 | rs79727797 | 145886836 | A | G | 2.4 | 2.3 | 3.8 × 10^-10^ | NA | NA | NA |
| 15 | 7 | rs74302792 | 6079993 | A | T | 15.9 | 0.8 | 7.4 × 10^-8^ | 21 | 0.51 | 0.32 |
| 5 | 8 | rs79136984 | 103213640 | T | G | 8.2 | -1.2 | 3.6 × 10^-9^ | 8 | -1.14 | 0.14 |
| 16 | 11 | rs7936234 | 96106737 | A | C | 19.6 | 0.6 | 1.7 × 10^-5^ | 22 | -0.07 | 0.88 |
| 10 | 15 | rs150393409 | 3120296 | A | G | 1.4 | -5.2 | 1.8 × 10^-28^ | NA | NA | NA |
|  | 15 | rs35811129 | 31241346 | A | G | 27.5 | 1.3 | 9.4 × 10^-26^ | 26 | 0.29 | 0.51 |
|  | 15 | rs151322829 | 31197995 | T | C | 0.7 | -3.8 | 1.4 × 10^-8^ | NA | NA | NA |
|  | 15 | rs3401747 | 31230611 | C | T | 38.2 | 0.8 | 8.5 × 10^-11^ | 36 | 0.41 | 0.31 |
| 17 | 19 | rs274883 | 48622545 | G | A | 16.7 | 0.9 | 5.3 × 10^-9^ | 24 | -0.54 | 0.25 |
|  | 19 | rs3730945 | 48645976 | G | C | 37.1 | -0.6 | 5.8 × 10^-7^ | 38 | 0.47 | 0.26 |
|  | 19 | rs14582163 | 48620943 | C | A | 0.1 | 7.7 | 1.5 × 10^-6^ | NA | NA | NA |
|  |  |  |  |  |  |  |  |  |  |  |  |
| *The loci supported by only one single rare SNP allele were not included. | | | | | | |  |  |  |  |  |
