# Supplementary material for: Genome-wide association study identifies genetic factors that modify age at onset in Machado-Joseph disease
Source: Aging (Albany NY). 2020 Mar 23;12(6):4742–56. doi: 10.18632/aging.102825 (PMC7138549; doi:10.18632/aging.102825)
Supplement: Supplementary Table 6 [file aging-12-102825-s003..docx]

**Supplementary Table 6.** **Gene sets and pathways enriched in i-GSEA4GWAS.**

| Pathway ID | P | permuted P |
| --- | --- | --- |
| GO:0000003_reproduction | < 1.00E-300 | < 1.00E-06 |
| GO:0002376_immune_system_process | < 1.00E-300 | < 1.00E-06 |
| GO:0003700_transcription_factor_activity | < 1.00E-300 | < 1.00E-06 |
| GO:0004672_protein_kinase_activity | < 1.00E-300 | < 1.00E-06 |
| GO:0005102_receptor_binding | < 1.00E-300 | < 1.00E-06 |
| GO:0005509_calcium_ion_binding | < 1.00E-300 | < 1.00E-06 |
| GO:0005624_membrane_fraction | < 1.00E-300 | < 1.00E-06 |
| GO:0005626_insoluble_fraction | < 1.00E-300 | < 1.00E-06 |
| GO:0005654_nucleoplasm | < 1.00E-300 | < 1.00E-06 |
| GO:0005783_endoplasmic_reticulum | < 1.00E-300 | < 1.00E-06 |
| GO:0005794_Golgi_apparatus | < 1.00E-300 | < 1.00E-06 |
| GO:0006357_regulation_of_transcription_from_RNA_polymerase_II_promoter | < 1.00E-300 | < 1.00E-06 |
| GO:0006468_protein_amino_acid_phosphorylation | < 1.00E-300 | < 1.00E-06 |
| GO:0006629_lipid_metabolic_process | < 1.00E-300 | < 1.00E-06 |
| GO:0006793_phosphorus_metabolic_process | < 1.00E-300 | < 1.00E-06 |
| GO:0006796_phosphate_metabolic_process | < 1.00E-300 | < 1.00E-06 |
| GO:0006811_ion_transport | < 1.00E-300 | < 1.00E-06 |
| GO:0007155_cell_adhesion | < 1.00E-300 | < 1.00E-06 |
| GO:0007267_cell-cell_signaling | < 1.00E-300 | < 1.00E-06 |
| GO:0008104_protein_localization | < 1.00E-300 | < 1.00E-06 |
| GO:0009605_response_to_external_stimulus | < 1.00E-300 | < 1.00E-06 |
| GO:0009887_organ_morphogenesis | < 1.00E-300 | < 1.00E-06 |
| GO:0009888_tissue_development | < 1.00E-300 | < 1.00E-06 |
| GO:0009891_positive_regulation_of_biosynthetic_process | < 1.00E-300 | < 1.00E-06 |
| GO:0009893_positive_regulation_of_metabolic_process | < 1.00E-300 | < 1.00E-06 |
| GO:0009966_regulation_of_signal_transduction | < 1.00E-300 | < 1.00E-06 |
| GO:0010033_response_to_organic_substance | < 1.00E-300 | < 1.00E-06 |
| GO:0010557_positive_regulation_of_macromolecule_biosynthetic_process | < 1.00E-300 | < 1.00E-06 |
| GO:0010604_positive_regulation_of_macromolecule_metabolic_process | < 1.00E-300 | < 1.00E-06 |
| GO:0010941_regulation_of_cell_death | < 1.00E-300 | < 1.00E-06 |
| GO:0012505_endomembrane_system | < 1.00E-300 | < 1.00E-06 |
| GO:0015031_protein_transport | < 1.00E-300 | < 1.00E-06 |
| GO:0015075_ion_transmembrane_transporter_activity | < 1.00E-300 | < 1.00E-06 |
| GO:0016301_kinase_activity | < 1.00E-300 | < 1.00E-06 |
| GO:0016310_phosphorylation | < 1.00E-300 | < 1.00E-06 |
| GO:0016462_pyrophosphatase_activity | < 1.00E-300 | < 1.00E-06 |
| GO:0016772_transferase_activity__transferring_phosphorus-containing_groups | < 1.00E-300 | < 1.00E-06 |
| GO:0016773_phosphotransferase_activity__alcohol_group_as_acceptor | < 1.00E-300 | < 1.00E-06 |
| GO:0016817_hydrolase_activity__acting_on_acid_anhydrides | < 1.00E-300 | < 1.00E-06 |
| GO:0016818_hydrolase_activity__acting_on_acid_anhydrides__in_phosphorus-containing_anhydrides | < 1.00E-300 | < 1.00E-06 |
| GO:0022008_neurogenesis | < 1.00E-300 | < 1.00E-06 |
| GO:0022414_reproductive_process | < 1.00E-300 | < 1.00E-06 |
| GO:0022607_cellular_component_assembly | < 1.00E-300 | < 1.00E-06 |
| GO:0022610_biological_adhesion | < 1.00E-300 | < 1.00E-06 |
| GO:0022857_transmembrane_transporter_activity | < 1.00E-300 | < 1.00E-06 |
| GO:0022891_substrate-specific_transmembrane_transporter_activity | < 1.00E-300 | < 1.00E-06 |
| GO:0022892_substrate-specific_transporter_activity | < 1.00E-300 | < 1.00E-06 |
| GO:0030054_cell_junction | < 1.00E-300 | < 1.00E-06 |
| GO:0030234_enzyme_regulator_activity | < 1.00E-300 | < 1.00E-06 |
| GO:0031325_positive_regulation_of_cellular_metabolic_process | < 1.00E-300 | < 1.00E-06 |
| GO:0031328_positive_regulation_of_cellular_biosynthetic_process | < 1.00E-300 | < 1.00E-06 |
| GO:0031410_cytoplasmic_vesicle | < 1.00E-300 | < 1.00E-06 |
| GO:0031982_vesicle | < 1.00E-300 | < 1.00E-06 |
| GO:0032879_regulation_of_localization | < 1.00E-300 | < 1.00E-06 |
| GO:0034645_cellular_macromolecule_biosynthetic_process | < 1.00E-300 | < 1.00E-06 |
| GO:0042127_regulation_of_cell_proliferation | < 1.00E-300 | < 1.00E-06 |
| GO:0042592_homeostatic_process | < 1.00E-300 | < 1.00E-06 |
| GO:0042981_regulation_of_apoptosis | < 1.00E-300 | < 1.00E-06 |
| GO:0042995_cell_projection | < 1.00E-300 | < 1.00E-06 |
| GO:0043067_regulation_of_programmed_cell_death | < 1.00E-300 | < 1.00E-06 |
| GO:0044085_cellular_component_biogenesis | < 1.00E-300 | < 1.00E-06 |
| GO:0044255_cellular_lipid_metabolic_process | < 1.00E-300 | < 1.00E-06 |
| GO:0044421_extracellular_region_part | < 1.00E-300 | < 1.00E-06 |
| GO:0044430_cytoskeletal_part | < 1.00E-300 | < 1.00E-06 |
| GO:0045184_establishment_of_protein_localization | < 1.00E-300 | < 1.00E-06 |
| GO:0048468_cell_development | < 1.00E-300 | < 1.00E-06 |
| GO:0048699_generation_of_neurons | < 1.00E-300 | < 1.00E-06 |
| GO:0050790_regulation_of_catalytic_activity | < 1.00E-300 | < 1.00E-06 |
| GO:0050793_regulation_of_developmental_process | < 1.00E-300 | < 1.00E-06 |
| GO:0051239_regulation_of_multicellular_organismal_process | < 1.00E-300 | < 1.00E-06 |
| GO:0051641_cellular_localization | < 1.00E-300 | < 1.00E-06 |
| GO:0051649_establishment_of_localization_in_cell | < 1.00E-300 | < 1.00E-06 |
| GO:0051716_cellular_response_to_stimulus | < 1.00E-300 | < 1.00E-06 |
| GO:0065009_regulation_of_molecular_function | < 1.00E-300 | < 1.00E-06 |
| PANTHER_BIOLOGICAL_PROCESS_Cell_adhesion | < 1.00E-300 | < 1.00E-06 |
| PANTHER_BIOLOGICAL_PROCESS_Neurogenesis | < 1.00E-300 | < 1.00E-06 |
| PANTHER_BIOLOGICAL_PROCESS_Protein_phosphorylation | < 1.00E-300 | < 1.00E-06 |
| PANTHER_BIOLOGICAL_PROCESS_Proteolysis | < 1.00E-300 | < 1.00E-06 |
| PC_EGF_receptor_(ErbB1)_signaling_pathway | < 1.00E-300 | < 1.00E-06 |
| PC_ErbB1_downstream_signaling | < 1.00E-300 | < 1.00E-06 |
| PC_ErbB_receptor_signaling_network | < 1.00E-300 | < 1.00E-06 |
| PC_Glypican_1_network | < 1.00E-300 | < 1.00E-06 |
| PC_Glypican_pathway | < 1.00E-300 | < 1.00E-06 |
| PC_Internalization_of_ErbB1 | < 1.00E-300 | < 1.00E-06 |
| PC_Proteogylcan_syndecan-mediated_signaling_events | < 1.00E-300 | < 1.00E-06 |
| PC_Syndecan-1-mediated_signaling_events | < 1.00E-300 | < 1.00E-06 |
| REACTOME_IMMUNE_SYSTEM | < 1.00E-300 | < 1.00E-06 |
| GO:0006928_cell_motion | 1.30E-297 | < 1.00E-06 |
| PANTHER_BIOLOGICAL_PROCESS_Cation_transport | 1.48E-297 | < 1.00E-06 |
| GO:0007417_central_nervous_system_development | 1.96E-280 | < 1.00E-06 |
| GO:0030182_neuron_differentiation | 8.24E-279 | < 1.00E-06 |
| GO:0030695_GTPase_regulator_activity | 3.56E-272 | < 1.00E-06 |
| GO:0043005_neuron_projection | 4.96E-268 | < 1.00E-06 |
| GO:0030030_cell_projection_organization | 6.79E-266 | < 1.00E-06 |
| GO:0030001_metal_ion_transport | 7.18E-265 | < 1.00E-06 |
| GO:0015267_channel_activity | 9.64E-259 | < 1.00E-06 |
| GO:0022803_passive_transmembrane_transporter_activity | 9.64E-259 | < 1.00E-06 |
| GO:0045202_synapse | 8.25E-258 | < 1.00E-06 |
| GO:0032989_cellular_component_morphogenesis | 1.09E-255 | < 1.00E-06 |
| GO:0022838_substrate_specific_channel_activity | 1.47E-254 | < 1.00E-06 |
| GO:0005216_ion_channel_activity | 1.69E-248 | < 1.00E-06 |
| GO:0000902_cell_morphogenesis | 7.91E-241 | < 1.00E-06 |
| GO:0019226_transmission_of_nerve_impulse | 7.27E-231 | < 1.00E-06 |
| GO:0046873_metal_ion_transmembrane_transporter_activity | 1.20E-221 | < 1.00E-06 |
| GO:0048666_neuron_development | 5.89E-217 | < 1.00E-06 |
| PANTHER_BIOLOGICAL_PROCESS_Cell_adhesion-mediated_signaling | 3.75E-215 | < 1.00E-06 |
| GO:0022836_gated_channel_activity | 2.97E-212 | < 1.00E-06 |
| GO:0007268_synaptic_transmission | 9.42E-206 | < 1.00E-06 |
| GO:0031175_neurite_development | 1.07E-202 | < 1.00E-06 |
| REACTOME_NEURONAL_SYSTEM | 1.29E-200 | < 1.00E-06 |
| GO:0005261_cation_channel_activity | 1.97E-197 | < 1.00E-06 |
| GO:0005083_small_GTPase_regulator_activity | 9.68E-195 | < 1.00E-06 |
| GO:0032990_cell_part_morphogenesis | 2.57E-192 | < 1.00E-06 |
| GO:0048858_cell_projection_morphogenesis | 2.30E-188 | < 1.00E-06 |
| GO:0044456_synapse_part | 1.25E-182 | < 1.00E-06 |
| GO:0016337_cell-cell_adhesion | 1.31E-181 | < 1.00E-06 |
| GO:0000904_cell_morphogenesis_involved_in_differentiation | 3.02E-176 | < 1.00E-06 |
| GO:0048812_neurite_morphogenesis | 1.27E-173 | < 1.00E-06 |
| GO:0048667_cell_morphogenesis_involved_in_neuron_differentiation | 5.93E-161 | < 1.00E-06 |
| PANTHER_MOLECULAR_FUNCTION_Other_receptor | 7.96E-157 | < 1.00E-06 |
| GO:0007409_axonogenesis | 4.30E-154 | < 1.00E-06 |
| GO:0034702_ion_channel_complex | 1.16E-146 | < 1.00E-06 |
| GO:0005244_voltage-gated_ion_channel_activity | 1.78E-138 | < 1.00E-06 |
| GO:0030425_dendrite | 9.45E-127 | < 1.00E-06 |
| GO:0045211_postsynaptic_membrane | 1.37E-119 | < 1.00E-06 |
| GO:0034703_cation_channel_complex | 3.02E-115 | < 1.00E-06 |
| GO:0070838_divalent_metal_ion_transport | 1.12E-111 | < 1.00E-06 |
| GO:0006816_calcium_ion_transport | 2.73E-111 | < 1.00E-06 |
| GO:0030955_potassium_ion_binding | 1.97E-95 | < 1.00E-06 |
| PANTHER_BIOLOGICAL_PROCESS_Calcium_mediated_signaling | 9.93E-94 | < 1.00E-06 |
| GO:0007411_axon_guidance | 2.10E-90 | < 1.00E-06 |
| GO:0015276_ligand-gated_ion_channel_activity | 2.69E-88 | < 1.00E-06 |
| GO:0014069_postsynaptic_density | 1.45E-77 | < 1.00E-06 |
| GO:0005262_calcium_channel_activity | 2.72E-77 | < 1.00E-06 |
| PANTHER_MOLECULAR_FUNCTION_CAM_family_adhesion_molecule | 1.71E-62 | < 1.00E-06 |
| GO:0008324_cation_transmembrane_transporter_activity | 4.97E-305 | 2.00E-06 |
| GO:0006812_cation_transport | 3.10E-299 | 2.00E-06 |
| GO:0060589_nucleoside-triphosphatase_regulator_activity | 4.91E-273 | 2.00E-06 |
| GO:0007420_brain_development | 5.26E-182 | 2.00E-06 |
| GO:0022832_voltage-gated_channel_activity | 1.78E-138 | 2.00E-06 |
| GO:0030424_axon | 2.27E-128 | 2.00E-06 |
| GO:0015674_di-__tri-valent_inorganic_cation_transport | 4.64E-123 | 2.00E-06 |
| GO:0006813_potassium_ion_transport | 6.17E-110 | 2.00E-06 |
| GO:0022834_ligand-gated_channel_activity | 2.69E-88 | 2.00E-06 |
| GO:0007612_learning | 6.44E-60 | 2.00E-06 |
| GO:0045296_cadherin_binding | 3.06E-20 | 2.00E-06 |
| GO:0008038_neuron_recognition | 7.59E-33 | 4.00E-06 |
| GO:0001964_startle_response | 1.68E-21 | 4.00E-06 |
| GO:0007610_behavior | 1.75E-266 | 6.00E-06 |
| GO:0050839_cell_adhesion_molecule_binding | 6.13E-34 | 6.00E-06 |
| GO:0008037_cell_recognition | 2.14E-54 | 8.00E-06 |
| PANTHER_BIOLOGICAL_PROCESS_Cell_communication | 6.09E-210 | 1.00E-05 |
| PANTHER_MOLECULAR_FUNCTION_Guanyl-nucleotide_exchange_factor | 2.25E-112 | 1.00E-05 |
| PID_RAC1_REG_PATHWAY | 7.25E-44 | 1.00E-05 |
| GO:0031420_alkali_metal_ion_binding | 4.98E-143 | 1.20E-05 |
| PANTHER_BIOLOGICAL_PROCESS_Neurotransmitter_release | 3.37E-89 | 1.20E-05 |
| GO:0022843_voltage-gated_cation_channel_activity | 3.68E-112 | 1.40E-05 |
| GO:0005267_potassium_channel_activity | 8.77E-101 | 1.40E-05 |
| PANTHER_MOLECULAR_FUNCTION_Voltage-gated_potassium_channel | 6.61E-80 | 1.40E-05 |
| GO:0031225_anchored_to_membrane | 5.25E-135 | 1.60E-05 |
| REACTOME_TRANSMISSION_ACROSS_CHEMICAL_SYNAPSES | 3.96E-133 | 1.60E-05 |
| GO:0003012_muscle_system_process | 2.74E-125 | 1.60E-05 |
| PANTHER_BIOLOGICAL_PROCESS_Other_neuronal_activity | 1.18E-105 | 1.60E-05 |
| REACTOME_CELL_CELL_COMMUNICATION | 7.13E-100 | 1.60E-05 |
| REACTOME_POTASSIUM_CHANNELS | 7.55E-81 | 1.60E-05 |
| GO:0005001_transmembrane_receptor_protein_tyrosine_phosphatase_activity | 1.16E-30 | 2.40E-05 |
| GO:0045595_regulation_of_cell_differentiation | 6.79E-292 | 2.80E-05 |
| GO:0019198_transmembrane_receptor_protein_phosphatase_activity | 1.16E-30 | 3.00E-05 |
| PANTHER_BIOLOGICAL_PROCESS_Sulfur_metabolism | 2.61E-47 | 3.20E-05 |
| PANTHER_MOLECULAR_FUNCTION_Other_ligand-gated_ion_channel | 4.65E-25 | 3.20E-05 |
| GO:0015629_actin_cytoskeleton | 6.64E-167 | 3.60E-05 |
| GO:0046903_secretion | 1.06E-184 | 3.80E-05 |
| REACTOME_NEUROTRANSMITTER_RECEPTOR_BINDING_AND_DOWNSTREAM_TRANSMISSION_IN_THE_POSTSYNAPTIC_CELL | 1.17E-96 | 4.00E-05 |
| GO:0034705_potassium_channel_complex | 4.28E-73 | 4.20E-05 |
| PANTHER_BIOLOGICAL_PROCESS_Nerve-nerve_synaptic_transmission | 2.42E-56 | 4.20E-05 |
| GO:0007626_locomotory_behavior | 1.94E-156 | 4.40E-05 |
| PANTHER_BIOLOGICAL_PROCESS_Developmental_processes | 1.87E-280 | 4.80E-05 |
| GO:0022037_metencephalon_development | 7.62E-37 | 4.80E-05 |
| GO:0030036_actin_cytoskeleton_organization | 2.18E-145 | 5.00E-05 |
| GO:0008076_voltage-gated_potassium_channel_complex | 4.28E-73 | 5.20E-05 |
| PANTHER_MOLECULAR_FUNCTION_Ion_channel | 7.11E-87 | 5.40E-05 |
| GO:0006936_muscle_contraction | 1.03E-114 | 5.60E-05 |
| GO:0030902_hindbrain_development | 5.28E-48 | 6.00E-05 |
| GO:0070161_anchoring_junction | 2.41E-132 | 6.40E-05 |
| PANTHER_BIOLOGICAL_PROCESS_Muscle_contraction | 4.72E-128 | 6.60E-05 |
| GO:0005913_cell-cell_adherens_junction | 9.27E-39 | 7.00E-05 |
| GO:0005217_intracellular_ligand-gated_ion_channel_activity | 2.89E-23 | 7.20E-05 |
| GO:0005912_adherens_junction | 9.70E-122 | 7.40E-05 |
| GO:0021549_cerebellum_development | 5.57E-34 | 7.40E-05 |
| PANTHER_MOLECULAR_FUNCTION_Transmembrane_receptor_regulatory/adaptor_protein | 1.41E-53 | 7.60E-05 |
| GO:0050905_neuromuscular_process | 9.47E-49 | 8.40E-05 |
| GO:0005096_GTPase_activator_activity | 5.34E-146 | 9.40E-05 |
| PC_CDC42_signaling_events | 5.05E-136 | 9.80E-05 |
| PANTHER_BIOLOGICAL_PROCESS_Synaptic_transmission | 1.84E-65 | 9.80E-05 |
| GO:0051674_localization_of_cell | 8.39E-186 | 0.000102 |
| GO:0005099_Ras_GTPase_activator_activity | 1.21E-61 | 0.000104 |
| GO:0008066_glutamate_receptor_activity | 6.17E-36 | 0.000106 |
| GO:0031012_extracellular_matrix | 1.29E-226 | 0.000108 |
| GO:0005911_cell-cell_junction | 8.00E-133 | 0.000108 |
| GO:0016323_basolateral_plasma_membrane | 8.12E-141 | 0.00011 |
| GO:0051056_regulation_of_small_GTPase_mediated_signal_transduction | 1.19E-166 | 0.000118 |
| GO:0034704_calcium_channel_complex | 1.00E-34 | 0.00012 |
| GO:0045664_regulation_of_neuron_differentiation | 5.03E-95 | 0.000122 |
| GO:0040011_locomotion | 4.27E-234 | 0.000124 |
| GO:0016917_GABA_receptor_activity | 3.60E-17 | 0.00013 |
| GO:0048870_cell_motility | 8.39E-186 | 0.000136 |
| GO:0004114_3'_5'-cyclic-nucleotide_phosphodiesterase_activity | 1.23E-35 | 0.00014 |
| GO:0004112_cyclic-nucleotide_phosphodiesterase_activity | 8.90E-37 | 0.000146 |
| GO:0050770_regulation_of_axonogenesis | 7.65E-48 | 0.00017 |
| GO:0007611_learning_or_memory | 1.06E-88 | 0.000174 |
| GO:0016477_cell_migration | 1.35E-168 | 0.000182 |
| GO:0005085_guanyl-nucleotide_exchange_factor_activity | 1.33E-119 | 0.000182 |
| GO:0044463_cell_projection_part | 1.74E-161 | 0.000184 |
| REACTOME_AXON_GUIDANCE | 2.42E-179 | 0.000188 |
| GO:0006939_smooth_muscle_contraction | 5.42E-34 | 0.000198 |
| GO:0021543_pallium_development | 2.34E-41 | 0.000206 |
| GO:0019725_cellular_homeostasis | 7.08E-243 | 0.000212 |
| GO:0019717_synaptosome | 5.33E-62 | 0.000218 |
| GO:0010975_regulation_of_neuron_projection_development | 1.16E-55 | 0.000218 |
| PC_Regulation_of_CDC42_activity | 3.12E-141 | 0.00022 |
| GO:0048813_dendrite_morphogenesis | 1.61E-15 | 0.000222 |
| GO:0042383_sarcolemma | 1.05E-54 | 0.000232 |
| GO:0043025_cell_soma | 9.12E-117 | 0.000236 |
| GO:0005097_Rab_GTPase_activator_activity | 3.93E-35 | 0.000252 |
| GO:0048878_chemical_homeostasis | 1.98E-271 | 0.000258 |
| GO:0003001_generation_of_a_signal_involved_in_cell-cell_signaling | 4.58E-59 | 0.000262 |
| GO:0032313_regulation_of_Rab_GTPase_activity | 2.46E-36 | 0.000276 |
| GO:0032483_regulation_of_Rab_protein_signal_transduction | 2.46E-36 | 0.000276 |
| GO:0032940_secretion_by_cell | 3.67E-134 | 0.000286 |
| GO:0031344_regulation_of_cell_projection_organization | 6.26E-65 | 0.000286 |
| GO:0008146_sulfotransferase_activity | 6.08E-46 | 0.000286 |
| GO:0005578_proteinaceous_extracellular_matrix | 3.74E-208 | 0.000288 |
| PID_IL8CXCR1_PATHWAY | 6.44E-26 | 0.000324 |
| PANTHER_MOLECULAR_FUNCTION_Annexin | 3.99E-61 | 0.000338 |
| GO:0009986_cell_surface | 2.34E-203 | 0.000342 |
| GO:0033267_axon_part | 4.11E-48 | 0.000346 |
| GO:0021537_telencephalon_development | 5.26E-54 | 0.00036 |
| GO:0004725_protein_tyrosine_phosphatase_activity | 4.02E-69 | 0.000386 |
| GO:0030029_actin_filament-based_process | 2.86E-148 | 4.00E-04 |
| GO:0016358_dendrite_development | 2.99E-33 | 4.00E-04 |
| REACTOME_VOLTAGE_GATED_POTASSIUM_CHANNELS | 1.04E-40 | 0.000406 |
| GO:0005230_extracellular_ligand-gated_ion_channel_activity | 1.15E-46 | 0.000416 |
| GO:0015672_monovalent_inorganic_cation_transport | 8.75E-178 | 0.000424 |
| GO:0006875_cellular_metal_ion_homeostasis | 4.21E-129 | 0.00044 |
| GO:0005088_Ras_guanyl-nucleotide_exchange_factor_activity | 1.11E-75 | 0.00044 |
| PANTHER_MOLECULAR_FUNCTION_Voltage-gated_calcium_channel | 5.39E-31 | 0.00044 |
| PANTHER_MOLECULAR_FUNCTION_Phosphodiesterase | 8.65E-42 | 0.00047 |
| PANTHER_BIOLOGICAL_PROCESS_Receptor_protein_tyrosine_kinase_signaling_pathway | 6.74E-147 | 0.00048 |
| GO:0045177_apical_part_of_cell | 1.03E-111 | 5.00E-04 |
| GO:0048706_embryonic_skeletal_system_development | 1.08E-47 | 5.00E-04 |
| REACTOME_HS_GAG_BIOSYNTHESIS | 2.23E-34 | 5.00E-04 |
| PANTHER_BIOLOGICAL_PROCESS_Neuronal_activities | 5.46E-95 | 0.000502 |
| GO:0051093_negative_regulation_of_developmental_process | 1.12E-150 | 0.000518 |
| REACTOME_ION_CHANNEL_TRANSPORT | 5.40E-41 | 0.00052 |
| GO:0045577_regulation_of_B_cell_differentiation | 5.78E-18 | 0.00052 |
| GO:0044057_regulation_of_system_process | 3.04E-196 | 0.00054 |
| GO:0007010_cytoskeleton_organization | 2.81E-233 | 0.00056 |
| GO:0007588_excretion | 9.78E-48 | 0.00058 |
| GO:0050767_regulation_of_neurogenesis | 5.56E-117 | 6.00E-04 |
| GO:0016529_sarcoplasmic_reticulum | 3.32E-35 | 6.00E-04 |
| GO:0030900_forebrain_development | 3.31E-100 | 0.00064 |
| REACTOME_GLYCOSAMINOGLYCAN_METABOLISM | 5.96E-78 | 0.00064 |
| GO:0043616_keratinocyte_proliferation | 1.91E-13 | 0.00064 |
| GO:0050890_cognition | 2.02E-295 | 0.00066 |
| GO:0016528_sarcoplasm | 3.32E-35 | 0.00068 |
| GO:0055085_transmembrane_transport | 1.62679634441455e-310 | 7.00E-04 |
| PANTHER_BIOLOGICAL_PROCESS_Metabolism_of_cyclic_nucleotides | 1.94E-48 | 7.00E-04 |
| PANTHER_BIOLOGICAL_PROCESS_Cell_structure | 9.87E-298 | 0.00072 |
| GO:0021953_central_nervous_system_neuron_differentiation | 8.25E-39 | 0.00072 |
| GO:0030032_lamellipodium_assembly | 6.41E-16 | 0.00072 |
| GO:0007167_enzyme_linked_receptor_protein_signaling_pathway | 9.58E-216 | 0.00074 |
| GO:0005245_voltage-gated_calcium_channel_activity | 2.74E-30 | 0.00078 |
| GO:0022604_regulation_of_cell_morphogenesis | 2.26E-97 | 8.00E-04 |
| GO:0005249_voltage-gated_potassium_channel_activity | 7.15E-74 | 0.00082 |
| PANTHER_MOLECULAR_FUNCTION_Other_cell_junction_protein | 2.28E-30 | 0.00082 |
| GO:0019899_enzyme_binding | 4.78E-300 | 0.00086 |
| GO:0050772_positive_regulation_of_axonogenesis | 1.97E-26 | 0.00086 |
| REACTOME_DEVELOPMENTAL_BIOLOGY | 2.16E-242 | 0.00088 |
| GO:0055065_metal_ion_homeostasis | 8.40E-133 | 0.00092 |
| GO:0005891_voltage-gated_calcium_channel_complex | 1.85E-27 | 0.00092 |
| GO:0016324_apical_plasma_membrane | 1.87E-89 | 0.00094 |
| PID_IL8CXCR2_PATHWAY | 4.72E-30 | 0.00096 |
| GO:0021587_cerebellum_morphogenesis | 1.57E-22 | 0.00096 |
| REACTOME_GABA_A_RECEPTOR_ACTIVATION | 4.31E-10 | 0.00096 |
| GO:0060402_calcium_ion_transport_into_cytosol | 1.83E-18 | 0.00098 |
| GO:0001501_skeletal_system_development | 2.97E-182 | 0.001 |
| GO:0022603_regulation_of_anatomical_structure_morphogenesis | 9.12E-146 | 0.001 |
| GO:0004385_guanylate_kinase_activity | 1.38E-14 | 0.001 |
| GO:0003779_actin_binding | 9.59E-208 | 0.00106 |
| PANTHER_BIOLOGICAL_PROCESS_Signal_transduction | 3.69E-162 | 0.00108 |
| GO:0045121_membrane_raft | 5.77E-98 | 0.00108 |
| REACTOME_HEPARAN_SULFATE_HEPARIN_HS_GAG_METABOLISM | 3.42E-42 | 0.00108 |
| PC_Neuroransmitter_Receptor_Binding_And_Downstream_Transmission_In_The_Postsynaptic_Cell | 1.93E-52 | 0.0011 |
| REACTOME_NETRIN1_SIGNALING | 3.51E-43 | 0.00112 |
| PANTHER_MOLECULAR_FUNCTION_GABA_receptor | 5.88E-13 | 0.00112 |
| GO:0050801_ion_homeostasis | 1.04E-219 | 0.00114 |
| GO:0016782_transferase_activity__transferring_sulfur-containing_groups | 3.36E-51 | 0.00114 |
| GO:0004890_GABA-A_receptor_activity | 5.88E-13 | 0.00114 |
| GO:0060401_cytosolic_calcium_ion_transport | 1.39E-19 | 0.00116 |
| GO:0007156_homophilic_cell_adhesion | 4.97E-74 | 0.00118 |
| PC_RAC1_signaling_pathway | 8.30E-116 | 0.0012 |
| PC_Regulation_of_RhoA_activity | 8.30E-116 | 0.00124 |
| PC_RhoA_signaling_pathway | 8.30E-116 | 0.00124 |
| REACTOME_LIGAND_GATED_ION_CHANNEL_TRANSPORT | 9.22E-16 | 0.00126 |
| PC_Regulation_of_RAC1_activity | 8.30E-116 | 0.00128 |
| GO:0010769_regulation_of_cell_morphogenesis_involved_in_differentiation | 3.90E-57 | 0.00128 |
| PID_TXA2PATHWAY | 3.47E-44 | 0.00128 |
| GO:0004697_protein_kinase_C_activity | 2.05E-18 | 0.00128 |
| GO:0021988_olfactory_lobe_development | 3.57E-14 | 0.00136 |
| REACTOME_TRANSMEMBRANE_TRANSPORT_OF_SMALL_MOLECULES | 8.80E-243 | 0.00138 |
| GO:0030534_adult_behavior | 6.39E-58 | 0.0014 |
| PANTHER_MOLECULAR_FUNCTION_Transcription_factor | 7.14E-92 | 0.00142 |
| REACTOME_EFFECTS_OF_PIP2_HYDROLYSIS | 5.20E-28 | 0.00142 |
| GO:0030201_heparan_sulfate_proteoglycan_metabolic_process | 1.88E-19 | 0.00142 |
| GO:0006836_neurotransmitter_transport | 4.45E-58 | 0.00146 |
| Panther_B_cell_activation | 2.65E-28 | 0.00152 |
| PANTHER_MOLECULAR_FUNCTION_Extracellular_matrix_glycoprotein | 5.70E-76 | 0.00164 |
| REACTOME_DSCAM_INTERACTIONS | 2.16E-15 | 0.00168 |
| PC_Synaptic_Transmission | 3.66E-78 | 0.00172 |
| GO:0004012_phospholipid-translocating_ATPase_activity | 5.32E-23 | 0.0018 |
| GO:0034656_nucleobase__nucleoside_and_nucleotide_catabolic_process | 4.96E-18 | 0.0018 |
| GO:0045596_negative_regulation_of_cell_differentiation | 1.36E-122 | 0.00182 |
| GO:0051960_regulation_of_nervous_system_development | 1.56E-130 | 0.00184 |
| GO:0006873_cellular_ion_homeostasis | 2.28E-199 | 0.00186 |
| GO:0005924_cell-substrate_adherens_junction | 3.69E-79 | 0.0019 |
| GO:0021575_hindbrain_morphogenesis | 4.07E-23 | 0.00192 |
| GO:0000122_negative_regulation_of_transcription_from_RNA_polymerase_II_promoter | 9.52E-159 | 0.002 |
| GO:0005516_calmodulin_binding | 1.07E-111 | 0.002 |
| GO:0060090_molecular_adaptor_activity | 4.95E-57 | 0.002 |
| REACTOME_GABA_RECEPTOR_ACTIVATION | 2.13E-41 | 0.002 |
| REACTOME_DAG_AND_IP3_SIGNALING | 3.57E-35 | 0.002 |
| GO:0009791_post-embryonic_development | 1.89E-49 | 0.00202 |
| GO:0050954_sensory_perception_of_mechanical_stimulus | 1.50E-83 | 0.00204 |
| GO:0005089_Rho_guanyl-nucleotide_exchange_factor_activity | 4.11E-64 | 0.0021 |
| PC_LPA_receptor_mediated_events | 4.23E-63 | 0.0021 |
| REACTOME_OPIOID_SIGNALLING | 3.79E-61 | 0.0021 |
| REACTOME_ADHERENS_JUNCTIONS_INTERACTIONS | 7.58E-33 | 0.0021 |
| GO:0021695_cerebellar_cortex_development | 3.65E-20 | 0.0021 |
| REACTOME_CELL_JUNCTION_ORGANIZATION | 3.07E-63 | 0.00214 |
| GO:0055080_cation_homeostasis | 1.61E-165 | 0.0022 |
| GO:0021987_cerebral_cortex_development | 6.08E-31 | 0.0022 |
| PID_REELINPATHWAY | 3.67E-30 | 0.0022 |
| GO:0015247_aminophospholipid_transporter_activity | 5.32E-23 | 0.0022 |
| GO:0034655_nucleobase__nucleoside__nucleotide_and_nucleic_acid_catabolic_process | 4.96E-18 | 0.0022 |
| GO:0051668_localization_within_membrane | 4.04E-15 | 0.00228 |
| GO:0055082_cellular_chemical_homeostasis | 2.28E-199 | 0.0023 |
| GO:0051899_membrane_depolarization | 1.07E-33 | 0.0023 |
| GO:0007269_neurotransmitter_secretion | 1.34E-26 | 0.0023 |
| GO:0051017_actin_filament_bundle_formation | 6.81E-20 | 0.0023 |
| GO:0033017_sarcoplasmic_reticulum_membrane | 1.54E-17 | 0.0023 |
| GO:0046578_regulation_of_Ras_protein_signal_transduction | 3.22E-135 | 0.00232 |
| Panther_Integrin_signalling_pathway | 2.40E-91 | 0.00232 |
| PANTHER_MOLECULAR_FUNCTION_G-protein_modulator | 1.21E-32 | 0.00238 |
| GO:0008284_positive_regulation_of_cell_proliferation | 6.48E-237 | 0.0024 |
| GO:0043087_regulation_of_GTPase_activity | 2.90E-78 | 0.0024 |
| GO:0007215_glutamate_signaling_pathway | 4.07E-29 | 0.0024 |
| BIOCARTA_EGFR_SMRTE_PATHWAY | 4.05E-16 | 0.0024 |
| GO:0045893_positive_regulation_of_transcription__DNA-dependent | 3.40E-245 | 0.0025 |
| PANTHER_MOLECULAR_FUNCTION_Other_signaling_molecule | 8.62E-145 | 0.0025 |
| GO:0030055_cell-substrate_junction | 8.53E-80 | 0.0025 |
| PC_Transmission_across_Chemical_Synapses | 7.38E-75 | 0.0025 |
| GO:0035108_limb_morphogenesis | 7.13E-64 | 0.0025 |
| PC_Reelin_signaling_pathway | 3.67E-30 | 0.0025 |
| GO:0051254_positive_regulation_of_RNA_metabolic_process | 2.52E-246 | 0.0026 |
| GO:0006874_cellular_calcium_ion_homeostasis | 6.37E-115 | 0.0026 |
| GO:0008344_adult_locomotory_behavior | 1.99E-44 | 0.0026 |
| GO:0034483_heparan_sulfate_sulfotransferase_activity | 1.88E-16 | 0.0026 |
| GO:0010647_positive_regulation_of_cell_communication | 5.67E-187 | 0.0028 |
| SIG_BCR_SIGNALING_PATHWAY | 3.89E-42 | 0.0028 |
| GO:0004970_ionotropic_glutamate_receptor_activity | 2.18E-21 | 0.0028 |
| GO:0043198_dendritic_shaft | 1.04E-14 | 0.0028 |
| REACTOME_PHOSPHOLIPASE_C_MEDIATED_CASCADE | 2.08E-50 | 0.0029 |
| PID_EPOPATHWAY | 1.44E-33 | 0.0029 |
| GO:0030169_low-density_lipoprotein_binding | 6.78E-26 | 0.0029 |
| GO:0005234_extracellular-glutamate-gated_ion_channel_activity | 2.18E-21 | 0.0029 |
| GO:0046928_regulation_of_neurotransmitter_secretion | 1.90E-20 | 0.0029 |
| GO:0015085_calcium_ion_transmembrane_transporter_activity | 7.61E-19 | 0.0029 |
| PID_ENDOTHELINPATHWAY | 1.18E-56 | 0.003 |
| PC_EPO_signaling_pathway | 1.44E-33 | 0.003 |
| GO:0016667_oxidoreductase_activity__acting_on_sulfur_group_of_donors | 6.74E-30 | 0.003 |
| GO:0003014_renal_system_process | 1.49E-27 | 0.003 |
| GO:0031327_negative_regulation_of_cellular_biosynthetic_process | 8.99E-276 | 0.0032 |
| REACTOME_SIGNALING_BY_PDGF | 1.43E-78 | 0.0032 |
| GO:0002790_peptide_secretion | 7.99E-29 | 0.0032 |
| GO:0060284_regulation_of_cell_development | 7.56E-138 | 0.0033 |
| GO:0007413_axonal_fasciculation | 1.59E-13 | 0.0033 |
| GO:0010628_positive_regulation_of_gene_expression | 2.12E-292 | 0.0034 |
| REACTOME_SIGNALING_BY_RHO_GTPASES | 2.15E-85 | 0.0034 |
| REACTOME_ANTIGEN_ACTIVATES_B_CELL_RECEPTOR_LEADING_TO_GENERATION_OF_SECOND_MESSENGERS | 8.82E-30 | 0.0034 |
| PC_CREB_phosphorylation_through_the_activation_of_CaMKII | 3.52E-16 | 0.0035 |
| GO:0045944_positive_regulation_of_transcription_from_RNA_polymerase_II_promoter | 1.42E-188 | 0.0036 |
| GO:0051172_negative_regulation_of_nitrogen_compound_metabolic_process | 3.25E-260 | 0.0037 |
| GO:0032403_protein_complex_binding | 2.13E-127 | 0.0037 |
| GO:0007605_sensory_perception_of_sound | 5.35E-78 | 0.0037 |
| GO:0021795_cerebral_cortex_cell_migration | 1.57E-17 | 0.0037 |
| PC_Ras_activation_uopn_Ca2+_infux_through_NMDA_receptor | 2.34E-17 | 0.0037 |
| GO:0005925_focal_adhesion | 1.22E-75 | 0.0038 |
| GO:0016566_specific_transcriptional_repressor_activity | 1.43E-24 | 0.0038 |
| GO:0045885_positive_regulation_of_survival_gene_product_expression | 9.07E-15 | 0.0038 |
| PC_Interactions_of_the_immunoglobulin_superfamily_(IgSF)_member_proteins | 6.22E-28 | 0.0039 |
| PC_Formation_of_Platelet_plug | 1.24E-119 | 0.004 |
| GO:0035107_appendage_morphogenesis | 7.13E-64 | 0.004 |
| GO:0030041_actin_filament_polymerization | 6.17E-12 | 0.004 |
| GO:0060173_limb_development | 6.18E-66 | 0.0041 |
| Panther_PDGF_signaling_pathway | 4.33E-34 | 0.0041 |
| REACTOME_GPCR_DOWNSTREAM_SIGNALING | 2.60E-271 | 0.0042 |
| GO:0016481_negative_regulation_of_transcription | 2.99E-233 | 0.0042 |
| GO:0048646_anatomical_structure_formation_involved_in_morphogenesis | 4.46E-199 | 0.0042 |
| PANTHER_BIOLOGICAL_PROCESS_Cell_motility | 8.25E-187 | 0.0042 |
| PANTHER_MOLECULAR_FUNCTION_Calmodulin_related_protein | 4.39E-94 | 0.0042 |
| PANTHER_MOLECULAR_FUNCTION_Signaling_molecule | 3.50E-45 | 0.0042 |
| GO:0051020_GTPase_binding | 1.59E-83 | 0.0043 |
| GO:0019904_protein_domain_specific_binding | 5.60E-197 | 0.0044 |
| GO:0030003_cellular_cation_homeostasis | 2.33E-145 | 0.0044 |
| REACTOME_NEPHRIN_INTERACTIONS | 6.59E-21 | 0.0044 |
| GO:0001662_behavioral_fear_response | 1.37E-19 | 0.0044 |
| GO:0031674_I_band | 5.09E-44 | 0.0045 |
| GO:0022804_active_transmembrane_transporter_activity | 1.02E-220 | 0.0046 |
| GO:0042578_phosphoric_ester_hydrolase_activity | 1.15E-197 | 0.0046 |
| GO:0033555_multicellular_organismal_response_to_stress | 4.93E-33 | 0.0046 |
| GO:0030288_outer_membrane-bounded_periplasmic_space | 2.64E-14 | 0.0046 |
| GO:0015291_secondary_active_transmembrane_transporter_activity | 2.09E-122 | 0.0047 |
| GO:0050808_synapse_organization | 2.37E-42 | 0.0047 |
| PC_Thromboxane_A2_receptor_signaling | 1.02E-29 | 0.0047 |
| PC_Unblocking_of_NMDA_receptor,_glutamate_binding_and_activation | 6.64E-17 | 0.0047 |
| PC_Class_C/3_(Metabotropic_glutamate/pheromone_receptors) | 1.55E-87 | 0.0049 |
| GO:0031346_positive_regulation_of_cell_projection_organization | 8.00E-38 | 0.0049 |
| GO:0005834_heterotrimeric_G-protein_complex | 6.22E-27 | 0.0049 |
| GO:0019199_transmembrane_receptor_protein_kinase_activity | 3.19E-72 | 0.005 |
| GO:0048736_appendage_development | 6.18E-66 | 0.005 |
| REACTOME_NRAGE_SIGNALS_DEATH_THROUGH_JNK | 3.14E-36 | 0.005 |
| PANTHER_MOLECULAR_FUNCTION_Glutamate_receptor | 3.37E-22 | 0.005 |
| GO:0005100_Rho_GTPase_activator_activity | 5.28E-21 | 0.005 |
| GO:0002209_behavioral_defense_response | 1.37E-19 | 0.005 |
| REACTOME_SIGNALING_BY_GPCR | 8.30277317836215e-320 | 0.0051 |
| GO:0006029_proteoglycan_metabolic_process | 8.06E-41 | 0.0051 |
| GO:0005548_phospholipid_transporter_activity | 2.63E-34 | 0.0051 |
| GO:0009187_cyclic_nucleotide_metabolic_process | 6.39E-34 | 0.0051 |
| GO:0046879_hormone_secretion | 8.97E-32 | 0.0051 |
| GO:0034311_diol_metabolic_process | 4.73E-28 | 0.0051 |
| REACTOME_EXTRACELLULAR_MATRIX_ORGANIZATION | 2.29E-63 | 0.0052 |
| GO:0007214_gamma-aminobutyric_acid_signaling_pathway | 8.33E-16 | 0.0052 |
| GO:0045934_negative_regulation_of_nucleobase__nucleoside__nucleotide_and_nucleic_acid_metabolic_process | 2.33E-257 | 0.0053 |
| PANTHER_BIOLOGICAL_PROCESS_Extracellular_matrix_protein-mediated_signaling | 3.85E-57 | 0.0053 |
| GO:0008154_actin_polymerization_or_depolymerization | 4.75E-17 | 0.0053 |
| GO:0007600_sensory_perception | 8.20E-237 | 0.0054 |
| GO:0042734_presynaptic_membrane | 4.60E-29 | 0.0054 |
| PANTHER_MOLECULAR_FUNCTION_Other_transferase | 4.71E-74 | 0.0055 |
| REACTOME_PLATELET_HOMEOSTASIS | 9.38E-67 | 0.0055 |
| PANTHER_MOLECULAR_FUNCTION_Other_oxidoreductase | 3.30E-24 | 0.0055 |
| GO:0009890_negative_regulation_of_biosynthetic_process | 6.93E-278 | 0.0056 |
| PANTHER_MOLECULAR_FUNCTION_Cadherin | 5.10E-49 | 0.0056 |
| GO:0009712_catechol_metabolic_process | 4.73E-28 | 0.0056 |
| GO:0015914_phospholipid_transport | 5.67E-35 | 0.0057 |
| GO:0022029_telencephalon_cell_migration | 3.06E-18 | 0.0057 |
| REACTOME_CREB_PHOSPHORYLATION_THROUGH_THE_ACTIVATION_OF_CAMKII | 1.03E-16 | 0.0058 |
| REACTOME_CELL_EXTRACELLULAR_MATRIX_INTERACTIONS | 2.33E-14 | 0.0058 |
| PC_Platelet_Activation | 3.36E-117 | 0.0059 |
| GO:0042277_peptide_binding | 1.90E-111 | 0.0059 |
| REACTOME_ION_TRANSPORT_BY_P_TYPE_ATPASES | 7.09E-32 | 0.0059 |
| GO:0018958_phenol_metabolic_process | 6.53E-29 | 0.0059 |
| GO:0042417_dopamine_metabolic_process | 4.04E-18 | 0.0059 |
| PC_Thrombin_signalling_through_proteinase_activated_receptors_(PARs) | 8.80E-78 | 0.006 |
| PC_Thromboxane_signalling_through_TP_receptor | 7.54E-74 | 0.006 |
| GO:0021885_forebrain_cell_migration | 3.06E-18 | 0.006 |
| GO:0051347_positive_regulation_of_transferase_activity | 4.37E-155 | 0.0061 |
| REACTOME_DOWNSTREAM_SIGNALING_OF_ACTIVATED_FGFR | 9.18E-72 | 0.0061 |
| REACTOME_COLLAGEN_FORMATION | 9.86E-48 | 0.0061 |
| PID_ERBB4_PATHWAY | 5.17E-28 | 0.0063 |
| GO:0008542_visual_learning | 5.28E-24 | 0.0064 |
| REACTOME_UNBLOCKING_OF_NMDA_RECEPTOR_GLUTAMATE_BINDING_AND_ACTIVATION | 1.29E-15 | 0.0064 |
| GO:0001764_neuron_migration | 2.49E-44 | 0.0065 |
| PID_RHOA_REG_PATHWAY | 4.47E-36 | 0.0065 |
| PC_ErbB4_signaling_events | 4.32E-24 | 0.0065 |
| GO:0045941_positive_regulation_of_transcription | 1.68E-282 | 0.0066 |
| GO:0055074_calcium_ion_homeostasis | 1.25E-118 | 0.0066 |
| GO:0007416_synaptogenesis | 2.14E-24 | 0.0066 |
| BIOCARTA_CELL2CELL_PATHWAY | 8.70E-17 | 0.0066 |
| GO:0045619_regulation_of_lymphocyte_differentiation | 5.45E-44 | 0.0067 |
| PC_VEGFR1_specific_signals | 1.09E-26 | 0.0067 |
| GO:0006584_catecholamine_metabolic_process | 4.73E-28 | 0.0068 |
| GO:0050848_regulation_of_calcium-mediated_signaling | 1.58E-19 | 0.0068 |
| PC_Signal_amplification | 1.61E-85 | 0.0069 |
| GO:0007204_elevation_of_cytosolic_calcium_ion_concentration | 2.77E-73 | 0.007 |
| GO:0030072_peptide_hormone_secretion | 1.10E-26 | 0.007 |
| Panther_Axon_guidance_mediated_by_netrin | 2.66E-20 | 0.007 |
| GO:0009914_hormone_transport | 2.00E-32 | 0.0072 |
| GO:0007270_nerve-nerve_synaptic_transmission | 9.43E-28 | 0.0072 |
| GO:0051588_regulation_of_neurotransmitter_transport | 8.69E-22 | 0.0072 |
| REACTOME_RAS_ACTIVATION_UOPN_CA2_INFUX_THROUGH_NMDA_RECEPTOR | 2.40E-20 | 0.0072 |
| GO:0021955_central_nervous_system_neuron_axonogenesis | 6.75E-18 | 0.0072 |
| GO:0042597_periplasmic_space | 2.64E-14 | 0.0072 |
| GO:0007389_pattern_specification_process | 8.18E-133 | 0.0073 |
| GO:0004857_enzyme_inhibitor_activity | 1.11E-119 | 0.0073 |
| GO:0042596_fear_response | 8.68E-22 | 0.0073 |
| GO:0048565_gut_development | 1.94E-33 | 0.0074 |
| GO:0008219_cell_death | 8.05918745145256e-317 | 0.0075 |
| GO:0051174_regulation_of_phosphorus_metabolic_process | 5.74E-244 | 0.0075 |
| GO:0042325_regulation_of_phosphorylation | 7.87E-239 | 0.0075 |
| GO:0005231_excitatory_extracellular_ligand-gated_ion_channel_activity | 1.21E-35 | 0.0075 |
| GO:0009593_detection_of_chemical_stimulus | 2.64E-25 | 0.0075 |
| GO:0051128_regulation_of_cellular_component_organization | 1.57E-238 | 0.0076 |
| GO:0009967_positive_regulation_of_signal_transduction | 2.51E-167 | 0.0076 |
| GO:0010817_regulation_of_hormone_levels | 6.30E-89 | 0.0076 |
| GO:0051705_behavioral_interaction_between_organisms | 4.03E-25 | 0.0076 |
| REACTOME_CHONDROITIN_SULFATE_DERMATAN_SULFATE_METABOLISM | 4.50E-36 | 0.0077 |
| GO:0060048_cardiac_muscle_contraction | 7.59E-18 | 0.0077 |
| GO:0016339_calcium-dependent_cell-cell_adhesion | 8.12E-13 | 0.0077 |
| GO:0044420_extracellular_matrix_part | 3.03E-83 | 0.0078 |
| GO:0032350_regulation_of_hormone_metabolic_process | 2.09E-15 | 0.0078 |
| PID_TCR_PATHWAY | 1.90E-49 | 0.0079 |
| BIOCARTA_FMLP_PATHWAY | 3.30E-31 | 0.0079 |
| GO:0008092_cytoskeletal_protein_binding | 4.08E-300 | 0.008 |
| GO:0007613_memory | 9.48E-36 | 0.008 |
| GO:0010558_negative_regulation_of_macromolecule_biosynthetic_process | 3.20E-264 | 0.0081 |
| GO:0007169_transmembrane_receptor_protein_tyrosine_kinase_signaling_pathway | 1.42E-153 | 0.0081 |
| GO:0008015_blood_circulation | 4.31E-118 | 0.0081 |
| PC_CXCR4-mediated_signaling_events | 3.05E-98 | 0.0081 |
| GO:0030031_cell_projection_assembly | 1.39E-58 | 0.0082 |
| GO:0045058_T_cell_selection | 5.64E-19 | 0.0082 |
| GO:0044449_contractile_fiber_part | 1.74E-76 | 0.0083 |
| REACTOME_REGULATION_OF_INSULIN_SECRETION | 3.53E-67 | 0.0083 |
| PID_LPA4_PATHWAY | 1.25E-16 | 0.0083 |
| GO:0042805_actinin_binding | 4.65E-14 | 0.0083 |
| SIG_PIP3_SIGNALING_IN_B_LYMPHOCYTES | 1.95E-34 | 0.0084 |
| GO:0050850_positive_regulation_of_calcium-mediated_signaling | 6.42E-18 | 0.0085 |
| REACTOME_CLASS_C_3_METABOTROPIC_GLUTAMATE_PHEROMONE_RECEPTORS | 1.25E-16 | 0.0085 |
| GO:0051969_regulation_of_transmission_of_nerve_impulse | 8.43E-100 | 0.0086 |
| GO:0043195_terminal_button | 1.03E-17 | 0.0086 |
| GO:0051318_G1_phase | 1.07E-17 | 0.0086 |
| GO:0050804_regulation_of_synaptic_transmission | 4.36E-94 | 0.0087 |
| GO:0007015_actin_filament_organization | 3.66E-47 | 0.0088 |
| GO:0016564_transcription_repressor_activity | 2.59E-175 | 0.0089 |
| GO:0033674_positive_regulation_of_kinase_activity | 3.03E-149 | 0.0089 |
| GO:0008081_phosphoric_diester_hydrolase_activity | 3.53E-75 | 0.0089 |
| PANTHER_BIOLOGICAL_PROCESS_Oncogene | 4.48E-66 | 0.0089 |
| GO:0042165_neurotransmitter_binding | 4.18E-57 | 0.0089 |
| GO:0043542_endothelial_cell_migration | 1.35E-22 | 0.0089 |
| REACTOME_CELL_CELL_JUNCTION_ORGANIZATION | 7.40E-46 | 0.009 |
| GO:0035094_response_to_nicotine | 1.06E-18 | 0.009 |
| GO:0045935_positive_regulation_of_nucleobase__nucleoside__nucleotide_and_nucleic_acid_metabolic_process | 1.09338701752984e-312 | 0.0091 |
| GO:0060047_heart_contraction | 7.84E-19 | 0.0091 |
| GO:0044462_external_encapsulating_structure_part | 1.60E-16 | 0.0091 |
| PC_Prostanoid_ligand_receptors | 3.52E-85 | 0.0092 |
| GO:0021696_cerebellar_cortex_morphogenesis | 1.51E-17 | 0.0092 |
| GO:0019220_regulation_of_phosphate_metabolic_process | 5.74E-244 | 0.0093 |
| GO:0007218_neuropeptide_signaling_pathway | 2.26E-56 | 0.0093 |
| GO:0003727_single-stranded_RNA_binding | 1.08E-22 | 0.0093 |
| GO:0030313_cell_envelope | 1.60E-16 | 0.0093 |
| GO:0031290_retinal_ganglion_cell_axon_guidance | 8.29E-18 | 0.0094 |
| GO:0055067_monovalent_inorganic_cation_homeostasis | 5.39E-33 | 0.0095 |
| GO:0051216_cartilage_development | 2.98E-57 | 0.0096 |
| PID_THROMBIN_PAR1_PATHWAY | 3.72E-32 | 0.0096 |
| GO:0003013_circulatory_system_process | 4.31E-118 | 0.0097 |
| GO:0048705_skeletal_system_morphogenesis | 2.09E-64 | 0.0097 |
| GO:0004222_metalloendopeptidase_activity | 1.91E-70 | 0.0098 |
| PC_Muscarinic_acetylcholine_receptors | 1.23E-82 | 0.0099 |
| BIOCARTA_TEL_PATHWAY | 2.24E-22 | 0.0099 |
| GO:0031324_negative_regulation_of_cellular_metabolic_process | 0.00E+00 | 0.01 |
| GO:0051897_positive_regulation_of_protein_kinase_B_signaling_cascade | 3.01E-11 | 0.01 |
| GO:0031644_regulation_of_neurological_system_process | 8.02E-102 | 0.0101 |
| GO:0004714_transmembrane_receptor_protein_tyrosine_kinase_activity | 2.90E-58 | 0.0101 |
| GO:0003015_heart_process | 7.84E-19 | 0.0101 |
| GO:0005615_extracellular_space | 8.75681950689025e-320 | 0.0102 |
| PANTHER_MOLECULAR_FUNCTION_Other_select_calcium_binding_proteins | 6.86E-32 | 0.0104 |
| REACTOME_CREB_PHOSPHORYLATION_THROUGH_THE_ACTIVATION_OF_RAS | 1.17E-23 | 0.0104 |
| PC_GABA_receptor_activation | 1.32E-18 | 0.0104 |
| GO:0043209_myelin_sheath | 3.77E-15 | 0.0106 |
| GO:0016265_death | 2.35521192185651e-317 | 0.0107 |
| GO:0060079_regulation_of_excitatory_postsynaptic_membrane_potential | 7.14E-19 | 0.0107 |
| GO:0070491_transcription_repressor_binding | 2.98E-16 | 0.0108 |
| REACTOME_SIGNALLING_BY_NGF | 2.79E-138 | 0.0109 |
| GO:0004713_protein_tyrosine_kinase_activity | 1.38E-120 | 0.0109 |
| PID_CDC42_PATHWAY | 5.44E-49 | 0.0109 |
| GO:0031280_negative_regulation_of_cyclase_activity | 5.99E-48 | 0.0109 |
| GO:0051173_positive_regulation_of_nitrogen_compound_metabolic_process | 4.79243676466009e-322 | 0.011 |
| PANTHER_MOLECULAR_FUNCTION_Protein_phosphatase | 2.40E-82 | 0.011 |
| BIOCARTA_CCR3_PATHWAY | 8.67E-19 | 0.011 |
| GO:0006470_protein_amino_acid_dephosphorylation | 3.56E-79 | 0.0111 |
| REACTOME_PLATELET_CALCIUM_HOMEOSTASIS | 3.87E-20 | 0.0111 |
| GO:0021533_cell_differentiation_in_hindbrain | 2.24E-12 | 0.0111 |
| GO:0006937_regulation_of_muscle_contraction | 1.47E-53 | 0.0112 |
| PANTHER_BIOLOGICAL_PROCESS_G-protein_mediated_signaling | 8.94E-304 | 0.0113 |
| PC_Platelet_activation_triggers | 2.05E-97 | 0.0113 |
| GO:0030017_sarcomere | 9.87E-66 | 0.0114 |
| GO:0007194_negative_regulation_of_adenylate_cyclase_activity | 5.99E-48 | 0.0114 |
| GO:0050864_regulation_of_B_cell_activation | 1.25E-31 | 0.0115 |
| GO:0008237_metallopeptidase_activity | 2.78E-113 | 0.0118 |
| GO:0015293_symporter_activity | 2.79E-91 | 0.0118 |
| GO:0051339_regulation_of_lyase_activity | 2.03E-73 | 0.0118 |
| GO:0051350_negative_regulation_of_lyase_activity | 5.99E-48 | 0.0118 |
| GO:0015297_antiporter_activity | 7.09E-42 | 0.0118 |
| GO:0030018_Z_disc | 3.38E-35 | 0.0118 |
| GO:0018107_peptidyl-threonine_phosphorylation | 1.11E-13 | 0.0118 |
| PANTHER_MOLECULAR_FUNCTION_Other_transporter | 4.90E-186 | 0.012 |
| GO:0032318_regulation_of_Ras_GTPase_activity | 1.12E-65 | 0.0121 |
| GO:0019897_extrinsic_to_plasma_membrane | 4.57E-42 | 0.0121 |
| GO:0031279_regulation_of_cyclase_activity | 2.03E-73 | 0.0123 |
| PID_P38_MK2PATHWAY | 1.02E-19 | 0.0123 |
| GO:0021952_central_nervous_system_projection_neuron_axonogenesis | 4.74E-15 | 0.0124 |
| GO:0090092_regulation_of_transmembrane_receptor_protein_serine/threonine_kinase_signaling_pathway | 5.25E-58 | 0.0126 |
| PANTHER_MOLECULAR_FUNCTION_Membrane-bound_signaling_molecule | 6.63E-80 | 0.0127 |
| GO:0051592_response_to_calcium_ion | 7.64E-39 | 0.0127 |
| GO:0019201_nucleotide_kinase_activity | 1.54E-18 | 0.0127 |
| GO:0014070_response_to_organic_cyclic_substance | 2.16E-86 | 0.0128 |
| ST_WNT_CA2_CYCLIC_GMP_PATHWAY | 3.36E-21 | 0.0128 |
| GO:0009790_embryonic_development | 5.59E-276 | 0.0129 |
| GO:0004674_protein_serine/threonine_kinase_activity | 5.58E-231 | 0.0129 |
| REACTOME_PLATELET_ACTIVATION_SIGNALING_AND_AGGREGATION | 8.89E-120 | 0.013 |
| GO:0050885_neuromuscular_process_controlling_balance | 1.36E-24 | 0.0132 |
| GO:0005581_collagen | 6.08E-27 | 0.0133 |
| GO:0007266_Rho_protein_signal_transduction | 1.57E-23 | 0.0133 |
| PC_Serotonin_receptors | 1.40E-87 | 0.0134 |
| GO:0019098_reproductive_behavior | 3.62E-16 | 0.0134 |
| GO:0008047_enzyme_activator_activity | 5.15E-188 | 0.0136 |
| PID_INTEGRIN4_PATHWAY | 3.82E-16 | 0.0138 |
| GO:0002062_chondrocyte_differentiation | 1.87E-12 | 0.0138 |
| GO:0042101_T_cell_receptor_complex | 7.05E-11 | 0.0138 |
| GO:0045086_positive_regulation_of_interleukin-2_biosynthetic_process | 1.30E-09 | 0.0138 |
| REACTOME_DOWNSTREAM_SIGNAL_TRANSDUCTION | 5.52E-60 | 0.014 |
| GO:0007292_female_gamete_generation | 3.47E-53 | 0.014 |
| GO:0048041_focal_adhesion_formation | 4.68E-16 | 0.014 |
| REACTOME_DCC_MEDIATED_ATTRACTIVE_SIGNALING | 6.11E-16 | 0.014 |
| GO:0009395_phospholipid_catabolic_process | 1.07E-19 | 0.0141 |
| REACTOME_TANDEM_PORE_DOMAIN_POTASSIUM_CHANNELS | 1.96E-12 | 0.0141 |
| GO:0005070_SH3/SH2_adaptor_activity | 1.21E-38 | 0.0143 |
| GO:0045860_positive_regulation_of_protein_kinase_activity | 1.83E-142 | 0.0144 |
| GO:0034707_chloride_channel_complex | 1.32E-31 | 0.0144 |
| REACTOME_POST_NMDA_RECEPTOR_ACTIVATION_EVENTS | 3.08E-29 | 0.0144 |
| GO:0030312_external_encapsulating_structure | 2.56E-17 | 0.0144 |
| PC_P2Y_receptors | 3.99E-83 | 0.0145 |
| PC_ADP_signalling_through_P2Y_purinoceptor_1 | 8.29E-69 | 0.0148 |
| REACTOME_PLC_BETA_MEDIATED_EVENTS | 3.54E-37 | 0.0149 |
| PID_VEGFR1_PATHWAY | 2.77E-23 | 0.0149 |
| REACTOME_G_ALPHA_S_SIGNALLING_EVENTS | 1.15E-80 | 0.0151 |
| PC_Cell_junction_organization | 3.36E-45 | 0.0152 |
| PID_EPHA_FWDPATHWAY | 2.59E-30 | 0.0154 |
| GO:0030315_T-tubule | 7.34E-21 | 0.0154 |
| GO:0046068_cGMP_metabolic_process | 8.93E-11 | 0.0154 |
| GO:0001816_cytokine_production | 7.81E-35 | 0.0155 |
| PID_RXR_VDR_PATHWAY | 1.63E-24 | 0.0155 |
| GO:0002020_protease_binding | 3.11E-17 | 0.0155 |
| PANTHER_MOLECULAR_FUNCTION_Extracellular_matrix_linker_protein | 1.17E-25 | 0.0156 |
| REACTOME_HS_GAG_DEGRADATION | 1.59E-18 | 0.0157 |
| GO:0045061_thymic_T_cell_selection | 1.87E-13 | 0.0157 |
| PID_RAS_PATHWAY | 3.37E-23 | 0.0158 |
| REACTOME_SIGNALING_BY_ERBB2 | 1.02E-69 | 0.016 |
| REACTOME_REGULATION_OF_INSULIN_SECRETION_BY_ACETYLCHOLINE | 3.47E-12 | 0.016 |
| GO:0030246_carbohydrate_binding | 7.36E-187 | 0.0162 |
| GO:0030005_cellular_di-__tri-valent_inorganic_cation_homeostasis | 6.95E-130 | 0.0162 |
| PC_Eicosanoid_ligand-binding_receptors | 3.26E-86 | 0.0163 |
| PANTHER_MOLECULAR_FUNCTION_Extracellular_matrix | 3.19E-75 | 0.0163 |
| GO:0008093_cytoskeletal_adaptor_activity | 9.66E-21 | 0.0165 |
| PC_Adrenoceptors | 4.92E-86 | 0.0166 |
| PC_Lectin_pathway_of_complement_activation | 2.31E-81 | 0.0166 |
| GO:0055123_digestive_system_development | 3.64E-23 | 0.0166 |
| GO:0035023_regulation_of_Rho_protein_signal_transduction | 9.37E-73 | 0.0168 |
| GO:0015269_calcium-activated_potassium_channel_activity | 3.07E-18 | 0.0168 |
| GO:0043062_extracellular_structure_organization | 2.95E-99 | 0.0169 |
| GO:0031267_small_GTPase_binding | 1.35E-71 | 0.0169 |
| GO:0048546_digestive_tract_morphogenesis | 3.64E-23 | 0.0169 |
| GO:0005901_caveola | 1.60E-40 | 0.017 |
| GO:0043679_nerve_terminal | 7.81E-16 | 0.0171 |
| GO:0045892_negative_regulation_of_transcription__DNA-dependent | 1.42E-194 | 0.0172 |
| GO:0008484_sulfuric_ester_hydrolase_activity | 1.53E-14 | 0.0172 |
| GO:0021761_limbic_system_development | 2.53E-25 | 0.0174 |
| PANTHER_BIOLOGICAL_PROCESS_Other_developmental_process | 4.78E-54 | 0.0175 |
| GO:0046483_heterocycle_metabolic_process | 9.39E-194 | 0.0176 |
| GO:0060341_regulation_of_cellular_localization | 1.67E-162 | 0.0176 |
| PID_DELTANP63PATHWAY | 4.98E-37 | 0.0176 |
| PC_Free_fatty_acid_receptors | 4.59E-68 | 0.0177 |
| GO:0030073_insulin_secretion | 6.18E-17 | 0.0182 |
| GO:0032102_negative_regulation_of_response_to_external_stimulus | 2.71E-31 | 0.0184 |
| GO:0030183_B_cell_differentiation | 8.50E-35 | 0.0185 |
| ST_MYOCYTE_AD_PATHWAY | 6.72E-23 | 0.0188 |
| GO:0003777_microtubule_motor_activity | 3.20E-48 | 0.0189 |
| GO:0045786_negative_regulation_of_cell_cycle | 7.84E-44 | 0.0189 |
| GO:0008201_heparin_binding | 4.08E-78 | 0.0192 |
| PC_IL2-mediated_signaling_events | 1.16E-74 | 0.0193 |
| REACTOME_ACTIVATION_OF_NMDA_RECEPTOR_exprON_GLUTAMATE_BINDING_AND_POSTSYNAPTIC_EVENTS | 4.67E-30 | 0.0193 |
| GO:0015833_peptide_transport | 7.66E-35 | 0.0194 |
| GO:0016234_inclusion_body | 1.05E-15 | 0.0194 |
| GO:0003774_motor_activity | 2.86E-92 | 0.0195 |
| GO:0001505_regulation_of_neurotransmitter_levels | 9.38E-44 | 0.0195 |
| GO:0010629_negative_regulation_of_gene_expression | 1.57E-244 | 0.02 |
| GO:0043068_positive_regulation_of_programmed_cell_death | 1.71E-227 | 0.02 |
| GO:0048471_perinuclear_region_of_cytoplasm | 3.00E-182 | 0.02 |
| GO:0009792_embryonic_development_ending_in_birth_or_egg_hatching | 6.66E-170 | 0.02 |
| REACTOME_INTEGRATION_OF_ENERGY_METABOLISM | 5.51E-82 | 0.02 |
| GO:0017016_Ras_GTPase_binding | 2.63E-66 | 0.02 |
| PC_BCR_signaling_pathway | 1.58E-44 | 0.02 |
| PID_CD8TCRPATHWAY | 3.61E-39 | 0.02 |
| GO:0005884_actin_filament | 4.72E-32 | 0.02 |
| GO:0017137_Rab_GTPase_binding | 3.79E-30 | 0.02 |
| REACTOME_INTERACTION_BETWEEN_L1_AND_ANKYRINS | 8.58E-28 | 0.02 |
| GO:0007632_visual_behavior | 1.94E-25 | 0.02 |
| GO:0035249_synaptic_transmission__glutamatergic | 1.31E-18 | 0.02 |
| GO:0018210_peptidyl-threonine_modification | 8.18E-16 | 0.02 |
| BIOCARTA_AKAPCENTROSOME_PATHWAY | 2.74E-13 | 0.02 |
| GO:0046716_muscle_maintenance | 1.06E-10 | 0.02 |
| GO:0014014_negative_regulation_of_gliogenesis | 4.06E-10 | 0.02 |
| GO:0007628_adult_walking_behavior | 3.33E-24 | 0.0203 |
| PANTHER_MOLECULAR_FUNCTION_Non-motor_actin_binding_protein | 5.31E-102 | 0.0204 |
| PC_Signaling_events_mediated_by_Hepatocyte_Growth_Factor_Receptor_(c-Met) | 3.92633968750039e-320 | 0.0207 |
| GO:0051253_negative_regulation_of_RNA_metabolic_process | 4.41E-195 | 0.0207 |
| PC_Cell-extracellular_matrix_interactions | 1.69E-14 | 0.0207 |
| GO:0060249_anatomical_structure_homeostasis | 3.90E-48 | 0.0209 |
| GO:0012501_programmed_cell_death | 1.06E-254 | 0.021 |
| GO:0006915_apoptosis | 9.40E-254 | 0.021 |
| REACTOME_METABOLISM_OF_CARBOHYDRATES | 2.35E-128 | 0.021 |
| GO:0050865_regulation_of_cell_activation | 1.47E-100 | 0.021 |
| REACTOME_SIGNALING_BY_FGFR | 9.35E-79 | 0.021 |
| PANTHER_BIOLOGICAL_PROCESS_Segment_specification | 2.20E-50 | 0.021 |
| PANTHER_MOLECULAR_FUNCTION_Select_regulatory_molecule | 5.91E-47 | 0.021 |
| GO:0045454_cell_redox_homeostasis | 5.37E-40 | 0.021 |
| PID_SYNDECAN_1_PATHWAY | 8.88E-31 | 0.021 |
| GO:0021954_central_nervous_system_neuron_development | 3.87E-29 | 0.021 |
| GO:0005227_calcium_activated_cation_channel_activity | 2.79E-21 | 0.021 |
| SA_B_CELL_RECEPTOR_COMPLEXES | 1.26E-19 | 0.021 |
| GO:0048265_response_to_pain | 6.52E-17 | 0.021 |
| GO:0008328_ionotropic_glutamate_receptor_complex | 6.29E-16 | 0.021 |
| GO:0005537_mannose_binding | 3.13E-13 | 0.021 |
| REACTOME_MYOGENESIS | 5.69E-24 | 0.0211 |
| GO:0016597_amino_acid_binding | 2.86E-41 | 0.0213 |
| PC_Transport_of_inorganic_cations/anions_and_amino_acids/oligopeptides | 8.13E-48 | 0.0214 |
| PID_BCR_5PATHWAY | 1.90E-44 | 0.0214 |
| GO:0030016_myofibril | 2.03E-74 | 0.0216 |
| GO:0010942_positive_regulation_of_cell_death | 3.33E-228 | 0.022 |
| GO:0051338_regulation_of_transferase_activity | 9.99E-199 | 0.022 |
| GO:0051046_regulation_of_secretion | 1.00E-127 | 0.022 |
| GO:0060348_bone_development | 5.16E-73 | 0.022 |
| PC_Opsins | 4.18E-66 | 0.022 |
| PANTHER_BIOLOGICAL_PROCESS_Extracellular_transport_and_import | 1.18E-50 | 0.022 |
| GO:0050730_regulation_of_peptidyl-tyrosine_phosphorylation | 6.63E-43 | 0.022 |
| PANTHER_MOLECULAR_FUNCTION_ATP-binding_cassette_(ABC)_transporter | 3.42E-33 | 0.022 |
| REACTOME_NITRIC_OXIDE_STIMULATES_GUANYLATE_CYCLASE | 4.49E-29 | 0.022 |
| REACTOME_ACTIVATION_OF_KAINATE_RECEPTORS_exprON_GLUTAMATE_BINDING | 4.40E-28 | 0.022 |
| GO:0060078_regulation_of_postsynaptic_membrane_potential | 2.03E-20 | 0.022 |
| GO:0005251_delayed_rectifier_potassium_channel_activity | 1.96E-14 | 0.022 |
| GO:0016079_synaptic_vesicle_exocytosis | 1.21E-10 | 0.022 |
| GO:0030035_microspike_assembly | 1.47E-21 | 0.0221 |
| GO:0000080_G1_phase_of_mitotic_cell_cycle | 3.60E-13 | 0.0222 |
| GO:0043954_cellular_component_maintenance | 5.94E-18 | 0.0223 |
| BIOCARTA_CDMAC_PATHWAY | 4.65E-18 | 0.0227 |
| GO:0030516_regulation_of_axon_extension | 5.97E-18 | 0.0227 |
| GO:0043065_positive_regulation_of_apoptosis | 3.84E-224 | 0.023 |
| GO:0071310_cellular_response_to_organic_substance | 2.85E-104 | 0.023 |
| PC_GPCR_downstream_signaling | 4.71E-87 | 0.023 |
| GO:0008509_anion_transmembrane_transporter_activity | 4.19E-83 | 0.023 |
| GO:0045761_regulation_of_adenylate_cyclase_activity | 3.37E-71 | 0.023 |
| PANTHER_BIOLOGICAL_PROCESS_Tumor_suppressor | 1.42E-54 | 0.023 |
| GO:0009123_nucleoside_monophosphate_metabolic_process | 7.80E-46 | 0.023 |
| GO:0043279_response_to_alkaloid | 3.13E-45 | 0.023 |
| PANTHER_MOLECULAR_FUNCTION_Cytoskeletal_protein | 1.05E-43 | 0.023 |
| GO:0005254_chloride_channel_activity | 7.53E-39 | 0.023 |
| PID_A6B1_A6B4_INTEGRIN_PATHWAY | 4.74E-37 | 0.023 |
| PANTHER_MOLECULAR_FUNCTION_Nuclear_hormone_receptor | 2.18E-35 | 0.023 |
| GO:0051262_protein_tetramerization | 1.69E-28 | 0.023 |
| GO:0050680_negative_regulation_of_epithelial_cell_proliferation | 1.40E-20 | 0.023 |
| GO:0045930_negative_regulation_of_mitotic_cell_cycle | 9.78E-20 | 0.023 |
| PC_Nucleotide-like_(purinergic)_receptors | 1.05E-87 | 0.0231 |
| GO:0003707_steroid_hormone_receptor_activity | 1.33E-36 | 0.0238 |
| GO:0007189_activation_of_adenylate_cyclase_activity_by_G-protein_signaling_pathway | 4.58E-32 | 0.0238 |
| PC_Signaling_events_mediated_by_focal_adhesion_kinase | 1.05808098466187e-310 | 0.024 |
| REACTOME_G_ALPHA1213_SIGNALLING_EVENTS | 1.33E-53 | 0.024 |
| PC_Amino_acid_and_oligopeptide_SLC_transporters | 8.13E-48 | 0.024 |
| GO:0006940_regulation_of_smooth_muscle_contraction | 4.47E-33 | 0.024 |
| PC_Cell-cell_junction_organization | 1.77E-20 | 0.024 |
| GO:0008306_associative_learning | 1.84E-15 | 0.024 |
| GO:0004065_arylsulfatase_activity | 7.74E-12 | 0.024 |
| GO:0071495_cellular_response_to_endogenous_stimulus | 6.53E-93 | 0.0241 |
| GO:0010559_regulation_of_glycoprotein_biosynthetic_process | 4.34E-13 | 0.0242 |
| GO:0005529_sugar_binding | 9.16E-92 | 0.0243 |
| GO:0045494_photoreceptor_cell_maintenance | 5.94E-18 | 0.0243 |
| GO:0007601_visual_perception | 4.68E-118 | 0.025 |
| PC_Creation_of_C4_and_C2_activators | 1.74E-82 | 0.025 |
| GO:0043292_contractile_fiber | 1.11E-79 | 0.025 |
| GO:0048704_embryonic_skeletal_system_morphogenesis | 9.54E-30 | 0.025 |
| GO:0016776_phosphotransferase_activity__phosphate_group_as_acceptor | 6.68E-25 | 0.025 |
| GO:0046058_cAMP_metabolic_process | 1.87E-22 | 0.025 |
| GO:0048538_thymus_development | 1.84E-16 | 0.025 |
| GO:0048639_positive_regulation_of_developmental_growth | 1.96E-14 | 0.025 |
| GO:0004930_G-protein_coupled_receptor_activity | 7.58E-229 | 0.026 |
| GO:0043009_chordate_embryonic_development | 6.66E-170 | 0.026 |
| PC_Amine_ligand-binding_receptors | 6.30E-94 | 0.026 |
| GO:0030817_regulation_of_cAMP_biosynthetic_process | 7.89E-77 | 0.026 |
| GO:0005604_basement_membrane | 3.78E-60 | 0.026 |
| PID_LYSOPHOSPHOLIPID_PATHWAY | 5.29E-55 | 0.026 |
| PANTHER_MOLECULAR_FUNCTION_Microtubule_binding_motor_protein | 6.81E-41 | 0.026 |
| Panther_Axon_guidance_mediated_by_Slit/Robo | 9.22E-18 | 0.026 |
| GO:0005796_Golgi_lumen | 3.67E-15 | 0.026 |
| Panther_VEGF_signaling_pathway | 8.59E-13 | 0.026 |
| GO:0060042_retina_morphogenesis_in_camera-type_eye | 2.30E-11 | 0.026 |
| PANTHER_MOLECULAR_FUNCTION_Glycosyltransferase | 9.00E-121 | 0.027 |
| PANTHER_MOLECULAR_FUNCTION_Extracellular_matrix_structural_protein | 1.54E-56 | 0.027 |
| GO:0010578_regulation_of_adenylate_cyclase_activity_involved_in_G-protein_signaling | 4.58E-32 | 0.027 |
| GO:0010579_positive_regulation_of_adenylate_cyclase_activity_by_G-protein_signaling_pathway | 4.58E-32 | 0.027 |
| GO:0005581_COL8A2_GO:0005581_updated_with_COL8A2 | 9.00E-28 | 0.027 |
| PC_GPCR_ligand_binding | 6.06E-193 | 0.0279 |
| REACTOME_SLC_MEDIATED_TRANSMEMBRANE_TRANSPORT | 5.15E-144 | 0.028 |
| PC_Histamine_receptors | 3.01E-86 | 0.028 |
| GO:0030155_regulation_of_cell_adhesion | 6.95E-82 | 0.028 |
| GO:0030808_regulation_of_nucleotide_biosynthetic_process | 1.69E-80 | 0.028 |
| GO:0008238_exopeptidase_activity | 2.59E-57 | 0.028 |
| GO:0010720_positive_regulation_of_cell_development | 1.84E-52 | 0.028 |
| PC_Organic_anion_transporters | 8.13E-48 | 0.028 |
| GO:0030139_endocytic_vesicle | 8.87E-40 | 0.028 |
| GO:0030140_trans-Golgi_network_transport_vesicle | 6.60E-19 | 0.028 |
| PC_p38_signaling_mediated_by_MAPKAP_kinases | 6.08E-18 | 0.028 |
| GO:0006066_cellular_alcohol_metabolic_process | 1.23E-207 | 0.029 |
| PC_Signaling_by_GPCR | 2.08E-193 | 0.029 |
| GO:0050953_sensory_perception_of_light_stimulus | 4.68E-118 | 0.029 |
| PANTHER_BIOLOGICAL_PROCESS_Cell_surface_receptor_mediated_signal_transduction | 2.57E-97 | 0.029 |
| GO:0009897_external_side_of_plasma_membrane | 1.35E-92 | 0.029 |
| PC_G_alpha_(q)_signalling_events | 4.18E-66 | 0.029 |
| GO:0031404_chloride_ion_binding | 2.84E-39 | 0.029 |
| GO:0060041_retina_development_in_camera-type_eye | 2.94E-21 | 0.029 |
| PC_CREB_phosphorylation_through_the_activation_of_Ras | 6.06E-21 | 0.029 |
| GO:0005086_ARF_guanyl-nucleotide_exchange_factor_activity | 6.10E-19 | 0.029 |
| GO:0021544_subpallium_development | 2.54E-10 | 0.029 |
| GO:0016491_oxidoreductase_activity | 2.04E-303 | 0.03 |
| PC_Axon_guidance | 9.61E-120 | 0.03 |
| GO:0019900_kinase_binding | 5.77E-108 | 0.03 |
| GO:0051480_cytosolic_calcium_ion_homeostasis | 7.87E-78 | 0.03 |
| GO:0002521_leukocyte_differentiation | 1.02E-74 | 0.03 |
| GO:0030098_lymphocyte_differentiation | 1.04E-62 | 0.03 |
| GO:0051048_negative_regulation_of_secretion | 1.82E-44 | 0.03 |
| GO:0004879_ligand-dependent_nuclear_receptor_activity | 7.80E-39 | 0.03 |
| PID_NETRIN_PATHWAY | 9.03E-30 | 0.03 |
| PC_Activation_of_NMDA_receptor_upon_glutamate_binding_and_postsynaptic_events | 2.89E-24 | 0.03 |
| GO:0031984_organelle_subcompartment | 1.61E-23 | 0.03 |
| GO:0051289_protein_homotetramerization | 6.42E-21 | 0.03 |
| REACTOME_IONOTROPIC_ACTIVITY_OF_KAINATE_RECEPTORS | 7.42E-14 | 0.03 |
| GO:0035004_phosphoinositide_3-kinase_activity | 3.38E-10 | 0.03 |
| GO:0030141_secretory_granule | 3.84E-106 | 0.031 |
| REACTOME_NCAM_SIGNALING_FOR_NEURITE_OUT_GROWTH | 2.22E-48 | 0.031 |
| GO:0045580_regulation_of_T_cell_differentiation | 4.46E-34 | 0.031 |
| PID_GLYPICAN_1PATHWAY | 1.70E-25 | 0.031 |
| BIOCARTA_SPPA_PATHWAY | 5.51E-25 | 0.031 |
| GO:0050921_positive_regulation_of_chemotaxis | 1.06E-24 | 0.031 |
| GO:0043500_muscle_adaptation | 1.50E-17 | 0.031 |
| PANTHER_BIOLOGICAL_PROCESS_Intracellular_signaling_cascade | 2.60E-141 | 0.032 |
| GO:0001932_regulation_of_protein_amino_acid_phosphorylation | 7.13E-110 | 0.032 |
| GO:0004721_phosphoprotein_phosphatase_activity | 7.24E-91 | 0.032 |
| GO:0016311_dephosphorylation | 3.19E-88 | 0.032 |
| GO:0032101_regulation_of_response_to_external_stimulus | 9.38E-85 | 0.032 |
| PC_Formyl_peptide_receptors_bind_formyl_peptides_and_many_other_ligands | 2.08E-74 | 0.032 |
| GO:0008277_regulation_of_G-protein_coupled_receptor_protein_signaling_pathway | 5.00E-34 | 0.032 |
| GO:0048863_stem_cell_differentiation | 1.93E-22 | 0.032 |
| GO:0045995_regulation_of_embryonic_development | 1.60E-16 | 0.032 |
| GO:0050926_regulation_of_positive_chemotaxis | 5.26E-14 | 0.032 |
| GO:0012502_induction_of_programmed_cell_death | 1.63E-177 | 0.033 |
| PC_Hemostasis | 1.52E-176 | 0.033 |
| PANTHER_MOLECULAR_FUNCTION_Metalloprotease | 2.55E-94 | 0.033 |
| GO:0030814_regulation_of_cAMP_metabolic_process | 4.09E-79 | 0.033 |
| GO:0043176_amine_binding | 6.83E-72 | 0.033 |
| REACTOME_TRANSPORT_OF_INORGANIC_CATIONS_ANIONS_AND_AMINO_ACIDS_OLIGOPEPTIDES | 3.43E-68 | 0.033 |
| GO:0050920_regulation_of_chemotaxis | 4.91E-27 | 0.033 |
| GO:0019898_extrinsic_to_membrane | 1.96E-276 | 0.034 |
| GO:0006917_induction_of_apoptosis | 5.72E-176 | 0.034 |
| GO:0016791_phosphatase_activity | 9.15E-131 | 0.034 |
| GO:0008194_UDP-glycosyltransferase_activity | 1.59E-72 | 0.034 |
| PID_HDAC_CLASSII_PATHWAY | 5.64E-25 | 0.034 |
| GO:0022839_ion_gated_channel_activity | 4.48E-20 | 0.034 |
| GO:0001990_regulation_of_systemic_arterial_blood_pressure_by_hormone | 3.80E-15 | 0.034 |
| GO:0016303_1-phosphatidylinositol-3-kinase_activity | 3.38E-10 | 0.034 |
| PC_Signaling_in_Immune_system | 2.43E-217 | 0.035 |
| PC_Chemokine_receptors_bind_chemokines | 2.23E-83 | 0.035 |
| GO:0001503_ossification | 2.67E-67 | 0.035 |
| GO:0005253_anion_channel_activity | 2.71E-43 | 0.035 |
| GO:0034329_cell_junction_assembly | 1.29E-41 | 0.035 |
| GO:0016486_peptide_hormone_processing | 1.56E-18 | 0.035 |
| GO:0050664_oxidoreductase_activity__acting_on_NADH_or_NADPH__with_oxygen_as_acceptor | 4.58E-10 | 0.035 |
| GO:0045730_respiratory_burst | 4.14E-09 | 0.035 |
| GO:0006461_protein_complex_assembly | 2.76E-227 | 0.036 |
| GO:0031252_cell_leading_edge | 3.87E-96 | 0.036 |
| GO:0030802_regulation_of_cyclic_nucleotide_biosynthetic_process | 1.69E-80 | 0.036 |
| PC_Activation_of_C3_and_C5 | 1.00E-77 | 0.036 |
| GO:0035113_embryonic_appendage_morphogenesis | 1.50E-53 | 0.036 |
| PC_RNA_Polymerase_III_Transcription_Termination | 1.62E-17 | 0.036 |
| PC_Complement_cascade | 2.39E-83 | 0.037 |
| GO:0046700_heterocycle_catabolic_process | 1.02E-60 | 0.037 |
| PANTHER_MOLECULAR_FUNCTION_Tyrosine_protein_kinase_receptor | 2.92E-59 | 0.037 |
| PANTHER_MOLECULAR_FUNCTION_Nucleotide_kinase | 8.33E-30 | 0.037 |
| REACTOME_AMINE_COMPOUND_SLC_TRANSPORTERS | 2.55E-24 | 0.037 |
| PID_EPHA2_FWDPATHWAY | 7.21E-14 | 0.037 |
| GO:0070887_cellular_response_to_chemical_stimulus | 8.42E-155 | 0.038 |
| GO:0007187_G-protein_signaling__coupled_to_cyclic_nucleotide_second_messenger | 6.31E-75 | 0.038 |
| PANTHER_MOLECULAR_FUNCTION_Cell_adhesion_molecule | 1.18E-66 | 0.038 |
| GO:0030414_peptidase_inhibitor_activity | 1.34E-65 | 0.038 |
| GO:0030594_neurotransmitter_receptor_activity | 1.18E-49 | 0.038 |
| GO:0030166_proteoglycan_biosynthetic_process | 1.78E-25 | 0.038 |
| GO:0009798_axis_specification | 4.65E-21 | 0.038 |
| GO:0046658_anchored_to_plasma_membrane | 1.19E-19 | 0.038 |
| GO:0035176_social_behavior | 1.25E-13 | 0.038 |
| GO:0045686_negative_regulation_of_glial_cell_differentiation | 4.06E-10 | 0.038 |
| GO:0005539_glycosaminoglycan_binding | 1.18E-96 | 0.039 |
| GO:0030326_embryonic_limb_morphogenesis | 1.50E-53 | 0.039 |
| GO:0016209_antioxidant_activity | 1.17E-28 | 0.039 |
| GO:0005080_protein_kinase_C_binding | 4.97E-21 | 0.039 |
| GO:0046631_alpha-beta_T_cell_activation | 2.92E-16 | 0.039 |
| GO:0001958_endochondral_ossification | 2.01E-11 | 0.039 |
| GO:0008593_regulation_of_Notch_signaling_pathway | 3.59E-11 | 0.039 |
| GO:0019838_growth_factor_binding | 2.43E-74 | 0.04 |
| PC_Signaling_events_regulated_by_Ret_tyrosine_kinase | 1.87E-46 | 0.04 |
| GO:0016247_channel_regulator_activity | 2.64E-41 | 0.04 |
| GO:0019902_phosphatase_binding | 5.30E-41 | 0.04 |
| REACTOME_CA_DEPENDENT_EVENTS | 2.63E-27 | 0.04 |
| BIOCARTA_NOS1_PATHWAY | 4.80E-23 | 0.04 |
| GO:0030512_negative_regulation_of_transforming_growth_factor_beta_receptor_signaling_pathway | 4.88E-16 | 0.04 |
| GO:0051896_regulation_of_protein_kinase_B_signaling_cascade | 8.45E-14 | 0.04 |
| GO:0050854_regulation_of_antigen_receptor-mediated_signaling_pathway | 1.49E-13 | 0.04 |
| GO:0009892_negative_regulation_of_metabolic_process | 1.92685601878086e-322 | 0.041 |
| GO:0051240_positive_regulation_of_multicellular_organismal_process | 1.86E-133 | 0.041 |
| PC_G_alpha_(i)_signalling_events | 1.25E-72 | 0.041 |
| GO:0005342_organic_acid_transmembrane_transporter_activity | 2.64E-66 | 0.041 |
| GO:0030278_regulation_of_ossification | 1.55E-57 | 0.041 |
| PID_TRKRPATHWAY | 1.28E-50 | 0.041 |
| GO:0050769_positive_regulation_of_neurogenesis | 5.83E-47 | 0.041 |
| PANTHER_BIOLOGICAL_PROCESS_Anion_transport | 8.63E-41 | 0.041 |
| GO:0040014_regulation_of_multicellular_organism_growth | 1.49E-37 | 0.041 |
| BIOCARTA_NO1_PATHWAY | 2.25E-34 | 0.041 |
| REACTOME_NCAM1_INTERACTIONS | 4.55E-29 | 0.041 |
| PC_RXR_and_RAR_heterodimerization_with_other_nuclear_receptor | 2.28E-19 | 0.041 |
| GO:0015850_organic_alcohol_transport | 3.68E-17 | 0.041 |
| PC_Adherens_junctions_interactions | 4.11E-14 | 0.041 |
| GO:0010605_negative_regulation_of_macromolecule_metabolic_process | 1.09E-306 | 0.042 |
| REACTOME_GASTRIN_CREB_SIGNALLING_PATHWAY_VIA_PKC_AND_MAPK | 1.84E-119 | 0.042 |
| GO:0051259_protein_oligomerization | 1.84E-100 | 0.042 |
| GO:0043168_anion_binding | 6.81E-49 | 0.042 |
| GO:0044447_axoneme_part | 6.80E-18 | 0.042 |
| GO:0001542_ovulation_from_ovarian_follicle | 1.97E-17 | 0.042 |
| GO:0050927_positive_regulation_of_positive_chemotaxis | 5.26E-14 | 0.042 |
| GO:0043069_negative_regulation_of_programmed_cell_death | 1.18E-192 | 0.043 |
| GO:0030799_regulation_of_cyclic_nucleotide_metabolic_process | 3.97E-83 | 0.043 |
| PID_IL2_1PATHWAY | 4.43E-40 | 0.043 |
| GO:0050768_negative_regulation_of_neurogenesis | 5.56E-32 | 0.043 |
| GO:0043549_regulation_of_kinase_activity | 7.05E-191 | 0.044 |
| PC_Initial_triggering_of_complement | 2.39E-83 | 0.044 |
| GO:0007229_integrin-mediated_signaling_pathway | 1.08E-47 | 0.044 |
| GO:0048701_embryonic_cranial_skeleton_morphogenesis | 3.94E-16 | 0.044 |
| PC_Myogenesis | 9.55E-15 | 0.044 |
| GO:0043560_insulin_receptor_substrate_binding | 2.18E-11 | 0.044 |
| GO:0060548_negative_regulation_of_cell_death | 1.18E-192 | 0.045 |
| GO:0045165_cell_fate_commitment | 8.56E-81 | 0.045 |
| PANTHER_BIOLOGICAL_PROCESS_T-cell_mediated_immunity | 5.15E-69 | 0.045 |
| GO:0051260_protein_homooligomerization | 8.94E-59 | 0.045 |
| GO:0033993_response_to_lipid | 4.88E-22 | 0.045 |
| GO:0050886_endocrine_process | 1.29E-17 | 0.045 |
| GO:0031294_lymphocyte_costimulation | 3.12E-12 | 0.045 |
| GO:0055066_di-__tri-valent_inorganic_cation_homeostasis | 1.87E-137 | 0.046 |
| PANTHER_BIOLOGICAL_PROCESS_Vision | 2.57E-100 | 0.046 |
| GO:0048511_rhythmic_process | 2.78E-89 | 0.046 |
| PC_Alternative_complement_activation | 1.38E-78 | 0.046 |
| PC_Adenosine_P1_receptors | 3.24E-77 | 0.046 |
| GO:0046943_carboxylic_acid_transmembrane_transporter_activity | 2.64E-66 | 0.046 |
| GO:0005275_amine_transmembrane_transporter_activity | 2.64E-57 | 0.046 |
| GO:0030879_mammary_gland_development | 2.76E-42 | 0.046 |
| PC_Post_NMDA_receptor_activation_events | 2.89E-24 | 0.046 |
| GO:0045884_regulation_of_survival_gene_product_expression | 3.81E-17 | 0.046 |
| GO:0004407_histone_deacetylase_activity | 9.23E-16 | 0.046 |
| GO:0045921_positive_regulation_of_exocytosis | 1.49E-12 | 0.046 |
| GO:0006163_purine_nucleotide_metabolic_process | 9.97E-111 | 0.047 |
| GO:0006690_icosanoid_metabolic_process | 2.51E-36 | 0.047 |
| PC_Signaling_events_mediated_by_HDAC_Class_II | 1.77E-25 | 0.047 |
| GO:0031985_Golgi_cisterna | 5.99E-22 | 0.047 |
| GO:0042058_regulation_of_epidermal_growth_factor_receptor_signaling_pathway | 1.22E-20 | 0.047 |
| GO:0033077_T_cell_differentiation_in_the_thymus | 1.92E-19 | 0.047 |
| GO:0030295_protein_kinase_activator_activity | 6.63E-16 | 0.047 |
| GO:0006949_syncytium_formation | 7.87E-16 | 0.047 |
| PC_Classical_antibody-mediated_complement_activation | 7.49E-79 | 0.048 |
| REACTOME_INTEGRIN_CELL_SURFACE_INTERACTIONS | 1.95E-56 | 0.048 |
| GO:0019229_regulation_of_vasoconstriction | 1.02E-37 | 0.048 |
| REACTOME_GABA_B_RECEPTOR_ACTIVATION | 6.03E-32 | 0.048 |
| GO:0043197_dendritic_spine | 1.20E-27 | 0.048 |
| GO:0008375_acetylglucosaminyltransferase_activity | 2.71E-26 | 0.048 |
| PANTHER_BIOLOGICAL_PROCESS_Antioxidation_and_free_radical_removal | 2.05E-20 | 0.048 |
| PANTHER_BIOLOGICAL_PROCESS_Sulfur_redox_metabolism | 1.86E-19 | 0.048 |
| REACTOME_DOWNSTREAM_TCR_SIGNALING | 3.68E-18 | 0.048 |
| GO:0021766_hippocampus_development | 4.92E-17 | 0.048 |
| GO:0007520_myoblast_fusion | 8.90E-15 | 0.048 |
| PC_CDO_in_myogenesis | 9.55E-15 | 0.048 |
| GO:0004016_adenylate_cyclase_activity | 1.23E-14 | 0.048 |
| GO:0015035_protein_disulfide_oxidoreductase_activity | 2.14E-12 | 0.048 |
| GO:0030291_protein_serine/threonine_kinase_inhibitor_activity | 2.81E-12 | 0.048 |
| GO:0043235_receptor_complex | 8.79E-85 | 0.049 |
| GO:0051924_regulation_of_calcium_ion_transport | 9.16E-46 | 0.049 |
| REACTOME_G_ALPHA_Z_SIGNALLING_EVENTS | 3.06E-39 | 0.049 |
| GO:0006941_striated_muscle_contraction | 7.91E-30 | 0.049 |
| PC_Amino_acid_transport_across_the_plasma_membrane | 3.87E-25 | 0.049 |
| GO:0046888_negative_regulation_of_hormone_secretion | 8.24E-22 | 0.049 |
| GO:0010811_positive_regulation_of_cell-substrate_adhesion | 2.43E-17 | 0.049 |
| GO:0045076_regulation_of_interleukin-2_biosynthetic_process | 1.54E-13 | 0.049 |
| GO:0002792_negative_regulation_of_peptide_secretion | 1.88E-13 | 0.049 |
| BIOCARTA_LECTIN_PATHWAY | 3.28E-11 | 0.049 |
| GO:0005095_GTPase_inhibitor_activity | 4.52E-11 | 0.049 |
