# Supplementary material for: Genome-wide association study identifies genetic factors that modify age at onset in Machado-Joseph disease
Source: Aging (Albany NY). 2020 Mar 23;12(6):4742–56. doi: 10.18632/aging.102825 (PMC7138549; doi:10.18632/aging.102825)
Supplement: Supplementary Table 7 [file aging-12-102825-s001..docx]

**Supplementary Table 7.** **Top gene sets and pathways enriched in PASCAL.**

| Pathway ID | P |
| --- | --- |
| GO_ANATOMICAL_STRUCTURE_FORMATION_INVOLVED_IN_MORPHOGENESIS | < 1.00E-16 |
| GO_ION_TRANSPORT | < 1.00E-16 |
| GO_NEUROLOGICAL_SYSTEM_PROCESS | < 1.00E-16 |
| GO_NEGATIVE_REGULATION_OF_GENE_EXPRESSION | < 1.00E-16 |
| GO_CHEMICAL_HOMEOSTASIS | < 1.00E-16 |
| GO_IMMUNE_SYSTEM_PROCESS | < 1.00E-16 |
| GO_POSITIVE_REGULATION_OF_RESPONSE_TO_STIMULUS | < 1.00E-16 |
| GO_POSITIVE_REGULATION_OF_CELL_COMMUNICATION | < 1.00E-16 |
| GO_REGULATION_OF_HYDROLASE_ACTIVITY | < 1.00E-16 |
| GO_CELL_CELL_SIGNALING | < 1.00E-16 |
| GO_SMALL_MOLECULE_METABOLIC_PROCESS | < 1.00E-16 |
| GO_HOMEOSTATIC_PROCESS | < 1.00E-16 |
| GO_REGULATION_OF_INTRACELLULAR_SIGNAL_TRANSDUCTION | < 1.00E-16 |
| GO_POSITIVE_REGULATION_OF_BIOSYNTHETIC_PROCESS | < 1.00E-16 |
| GO_NEGATIVE_REGULATION_OF_NITROGEN_COMPOUND_METABOLIC_PROCESS | < 1.00E-16 |
| GO_REGULATION_OF_NERVOUS_SYSTEM_DEVELOPMENT | < 1.00E-16 |
| GO_ESTABLISHMENT_OF_LOCALIZATION_IN_CELL | < 1.00E-16 |
| GO_VESICLE_MEDIATED_TRANSPORT | < 1.00E-16 |
| GO_POSITIVE_REGULATION_OF_CELLULAR_COMPONENT_ORGANIZATION | < 1.00E-16 |
| GO_POSITIVE_REGULATION_OF_GENE_EXPRESSION | < 1.00E-16 |
| GO_CARBOHYDRATE_DERIVATIVE_METABOLIC_PROCESS | < 1.00E-16 |
| GO_CATION_TRANSPORT | < 1.00E-16 |
| GO_POSITIVE_REGULATION_OF_HYDROLASE_ACTIVITY | < 1.00E-16 |
| GO_RESPONSE_TO_ENDOGENOUS_STIMULUS | < 1.00E-16 |
| GO_POSITIVE_REGULATION_OF_CATALYTIC_ACTIVITY | < 1.00E-16 |
| GO_RESPONSE_TO_EXTERNAL_STIMULUS | < 1.00E-16 |
| GO_ION_TRANSMEMBRANE_TRANSPORT | < 1.00E-16 |
| GO_INTRACELLULAR_SIGNAL_TRANSDUCTION | < 1.00E-16 |
| GO_REGULATION_OF_CELL_PROJECTION_ORGANIZATION | < 1.00E-16 |
| GO_POSITIVE_REGULATION_OF_MOLECULAR_FUNCTION | < 1.00E-16 |
| GO_CELLULAR_RESPONSE_TO_ORGANIC_SUBSTANCE | < 1.00E-16 |
| GO_POSITIVE_REGULATION_OF_PHOSPHORUS_METABOLIC_PROCESS | < 1.00E-16 |
| GO_REGULATION_OF_CELLULAR_LOCALIZATION | < 1.00E-16 |
| GO_REGULATION_OF_MULTICELLULAR_ORGANISMAL_DEVELOPMENT | < 1.00E-16 |
| GO_MACROMOLECULAR_COMPLEX_ASSEMBLY | < 1.00E-16 |
| GO_ORGAN_MORPHOGENESIS | < 1.00E-16 |
| GO_POSITIVE_REGULATION_OF_MULTICELLULAR_ORGANISMAL_PROCESS | < 1.00E-16 |
| GO_G_PROTEIN_COUPLED_RECEPTOR_SIGNALING_PATHWAY | < 1.00E-16 |
| GO_REGULATION_OF_RESPONSE_TO_STRESS | < 1.00E-16 |
| GO_PHOSPHATE_CONTAINING_COMPOUND_METABOLIC_PROCESS | < 1.00E-16 |
| GO_REGULATION_OF_CELL_DIFFERENTIATION | < 1.00E-16 |
| GO_REGULATION_OF_IMMUNE_SYSTEM_PROCESS | < 1.00E-16 |
| GO_RESPONSE_TO_OXYGEN_CONTAINING_COMPOUND | < 1.00E-16 |
| GO_LOCOMOTION | < 1.00E-16 |
| GO_NEUROGENESIS | < 1.00E-16 |
| GO_SYSTEM_PROCESS | < 1.00E-16 |
| GO_MOVEMENT_OF_CELL_OR_SUBCELLULAR_COMPONENT | < 1.00E-16 |
| GO_POSITIVE_REGULATION_OF_PROTEIN_METABOLIC_PROCESS | < 1.00E-16 |
| GO_RESPONSE_TO_ABIOTIC_STIMULUS | < 1.00E-16 |
| GO_POSITIVE_REGULATION_OF_INTRACELLULAR_SIGNAL_TRANSDUCTION | < 1.00E-16 |
| GO_HEAD_DEVELOPMENT | < 1.00E-16 |
| GO_ORGANONITROGEN_COMPOUND_METABOLIC_PROCESS | < 1.00E-16 |
| GO_POSITIVE_REGULATION_OF_PROTEIN_MODIFICATION_PROCESS | < 1.00E-16 |
| GO_NEGATIVE_REGULATION_OF_DEVELOPMENTAL_PROCESS | < 1.00E-16 |
| GO_REGULATION_OF_ANATOMICAL_STRUCTURE_MORPHOGENESIS | < 1.00E-16 |
| GO_REGULATION_OF_CELL_DEATH | < 1.00E-16 |
| GO_NEGATIVE_REGULATION_OF_RESPONSE_TO_STIMULUS | < 1.00E-16 |
| GO_PROTEIN_LOCALIZATION | < 1.00E-16 |
| GO_CELLULAR_RESPONSE_TO_ENDOGENOUS_STIMULUS | < 1.00E-16 |
| GO_CELL_PROJECTION_ORGANIZATION | < 1.00E-16 |
| GO_NEGATIVE_REGULATION_OF_PROTEIN_METABOLIC_PROCESS | < 1.00E-16 |
| GO_PROTEIN_COMPLEX_SUBUNIT_ORGANIZATION | < 1.00E-16 |
| GO_NEURON_DIFFERENTIATION | < 1.00E-16 |
| GO_POSITIVE_REGULATION_OF_DEVELOPMENTAL_PROCESS | < 1.00E-16 |
| GO_REGULATION_OF_PROTEIN_MODIFICATION_PROCESS | < 1.00E-16 |
| GO_POSITIVE_REGULATION_OF_TRANSPORT | < 1.00E-16 |
| GO_REGULATION_OF_TRANSPORT | < 1.00E-16 |
| GO_POSITIVE_REGULATION_OF_CELL_DIFFERENTIATION | < 1.00E-16 |
| GO_REGULATION_OF_ION_TRANSPORT | < 1.00E-16 |
| GO_CELLULAR_RESPONSE_TO_STRESS | < 1.00E-16 |
| GO_CELL_DEVELOPMENT | < 1.00E-16 |
| GO_REGULATION_OF_ORGANELLE_ORGANIZATION | < 1.00E-16 |
| GO_PHOSPHORYLATION | < 1.00E-16 |
| GO_NEGATIVE_REGULATION_OF_MULTICELLULAR_ORGANISMAL_PROCESS | < 1.00E-16 |
| GO_REGULATION_OF_CELL_DEVELOPMENT | < 1.00E-16 |
| GO_CATABOLIC_PROCESS | < 1.00E-16 |
| GO_TRANSMEMBRANE_TRANSPORT | < 1.00E-16 |
| GO_INORGANIC_ION_TRANSMEMBRANE_TRANSPORT | < 1.00E-16 |
| GO_CENTRAL_NERVOUS_SYSTEM_DEVELOPMENT | < 1.00E-16 |
| GO_REGULATION_OF_PHOSPHORUS_METABOLIC_PROCESS | < 1.00E-16 |
| GO_REGULATION_OF_CELL_PROLIFERATION | < 1.00E-16 |
| GO_BIOLOGICAL_ADHESION | < 1.00E-16 |
| GO_TISSUE_DEVELOPMENT | < 1.00E-16 |
| GO_NEGATIVE_REGULATION_OF_CELL_COMMUNICATION | < 1.00E-16 |
| GO_REGULATION_OF_TRANSCRIPTION_FROM_RNA_POLYMERASE_II_PROMOTER | < 1.00E-16 |
| GO_VACUOLE | < 1.00E-16 |
| GO_NEURON_PART | < 1.00E-16 |
| GO_INTRINSIC_COMPONENT_OF_PLASMA_MEMBRANE | < 1.00E-16 |
| GO_CELL_JUNCTION | < 1.00E-16 |
| GO_CYTOSKELETON | < 1.00E-16 |
| GO_INTRACELLULAR_VESICLE | < 1.00E-16 |
| GO_SYNAPSE | < 1.00E-16 |
| GO_NEURON_PROJECTION | < 1.00E-16 |
| GO_EXTRACELLULAR_SPACE | < 1.00E-16 |
| GO_MITOCHONDRION | < 1.00E-16 |
| GO_CELL_SURFACE | < 1.00E-16 |
| GO_TRANSPORTER_COMPLEX | < 1.00E-16 |
| GO_MEMBRANE_PROTEIN_COMPLEX | < 1.00E-16 |
| GO_PLASMA_MEMBRANE_REGION | < 1.00E-16 |
| GO_CELL_PROJECTION | < 1.00E-16 |
| GO_CELL_PROJECTION_PART | < 1.00E-16 |
| GO_GOLGI_APPARATUS | < 1.00E-16 |
| GO_ENDOPLASMIC_RETICULUM | < 1.00E-16 |
| GO_MEMBRANE_REGION | < 1.00E-16 |
| GO_SYNAPSE_PART | < 1.00E-16 |
| GO_PLASMA_MEMBRANE_PROTEIN_COMPLEX | < 1.00E-16 |
| GO_SIGNAL_TRANSDUCER_ACTIVITY | < 1.00E-16 |
| GO_CYTOSKELETAL_PROTEIN_BINDING | < 1.00E-16 |
| GO_PROTEIN_DIMERIZATION_ACTIVITY | < 1.00E-16 |
| GO_GATED_CHANNEL_ACTIVITY | < 1.00E-16 |
| GO_PASSIVE_TRANSMEMBRANE_TRANSPORTER_ACTIVITY | < 1.00E-16 |
| GO_CALCIUM_ION_BINDING | < 1.00E-16 |
| GO_SIGNALING_RECEPTOR_ACTIVITY | < 1.00E-16 |
| GO_NUCLEIC_ACID_BINDING_TRANSCRIPTION_FACTOR_ACTIVITY | < 1.00E-16 |
| GO_MOLECULAR_FUNCTION_REGULATOR | < 1.00E-16 |
| GO_IDENTICAL_PROTEIN_BINDING | < 1.00E-16 |
| GO_TRANSMEMBRANE_TRANSPORTER_ACTIVITY | < 1.00E-16 |
| GO_RIBONUCLEOTIDE_BINDING | < 1.00E-16 |
| GO_ENZYME_REGULATOR_ACTIVITY | < 1.00E-16 |
| GO_ENZYME_BINDING | < 1.00E-16 |
| GO_RECEPTOR_ACTIVITY | < 1.00E-16 |
| GO_ADENYL_NUCLEOTIDE_BINDING | < 1.00E-16 |
| GO_RECEPTOR_BINDING | < 1.00E-16 |
| GO_TRANSPORTER_ACTIVITY | < 1.00E-16 |
| GO_MACROMOLECULAR_COMPLEX_BINDING | < 1.00E-16 |
| GO_NEURON_DEVELOPMENT | 1.11E-16 |
| GO_CELL_DEATH | 1.11E-16 |
| GO_POSITIVE_REGULATION_OF_CELL_PROLIFERATION | 1.11E-16 |
| GO_REGULATION_OF_NEURON_DIFFERENTIATION | 1.11E-16 |
| GO_REGULATION_OF_GTPASE_ACTIVITY | 1.11E-16 |
| GO_SOMATODENDRITIC_COMPARTMENT | 1.11E-16 |
| GO_SEQUENCE_SPECIFIC_DNA_BINDING | 1.11E-16 |
| GO_TRANSFERASE_ACTIVITY_TRANSFERRING_PHOSPHORUS_CONTAINING_GROUPS | 1.11E-16 |
| GO_EPITHELIUM_DEVELOPMENT | 2.22E-16 |
| GO_MEMBRANE_ORGANIZATION | 2.22E-16 |
| GO_GOLGI_APPARATUS_PART | 2.22E-16 |
| GO_NEGATIVE_REGULATION_OF_MOLECULAR_FUNCTION | 5.55E-16 |
| GO_LIPID_METABOLIC_PROCESS | 5.55E-16 |
| GO_ESTABLISHMENT_OF_PROTEIN_LOCALIZATION | 8.88E-16 |
| GO_TRANSITION_METAL_ION_BINDING | 1.44E-15 |
| GO_METAL_ION_TRANSPORT | 1.78E-15 |
| GO_PROTEIN_COMPLEX_BIOGENESIS | 2.11E-15 |
| GO_CELL_MOTILITY | 3.55E-15 |
| GO_ENDOPLASMIC_RETICULUM_PART | 3.66E-15 |
| GO_REGULATION_OF_CELLULAR_COMPONENT_MOVEMENT | 6.44E-15 |
| GO_REGULATION_OF_RESPONSE_TO_EXTERNAL_STIMULUS | 7.11E-15 |
| GO_PROTEIN_COMPLEX_BINDING | 8.22E-15 |
| GO_METAL_ION_TRANSMEMBRANE_TRANSPORTER_ACTIVITY | 8.33E-15 |
| GO_CELLULAR_COMPONENT_MORPHOGENESIS | 8.77E-15 |
| GO_CELL_CELL_ADHESION | 1.34E-14 |
| GO_CATION_TRANSMEMBRANE_TRANSPORTER_ACTIVITY | 1.34E-14 |
| GO_KINASE_ACTIVITY | 1.92E-14 |
| GO_CELLULAR_HOMEOSTASIS | 2.29E-14 |
| GO_NEGATIVE_REGULATION_OF_CELL_DEATH | 2.94E-14 |
| GO_CELLULAR_MACROMOLECULE_LOCALIZATION | 3.46E-14 |
| GO_CYTOSKELETAL_PART | 3.69E-14 |
| GO_REGULATION_OF_TRANSFERASE_ACTIVITY | 6.03E-14 |
| GO_REGULATION_OF_TRANSMEMBRANE_TRANSPORT | 7.44E-14 |
| GO_REGULATION_OF_NEURON_PROJECTION_DEVELOPMENT | 8.23E-14 |
| GO_REGULATION_OF_CELL_MORPHOGENESIS | 8.47E-14 |
| GO_ENZYME_LINKED_RECEPTOR_PROTEIN_SIGNALING_PATHWAY | 1.09E-13 |
| GO_INORGANIC_CATION_TRANSMEMBRANE_TRANSPORTER_ACTIVITY | 1.42E-13 |
| REACTOME_SIGNALING_BY_GPCR | 1.48E-13 |
| GO_CATION_CHANNEL_ACTIVITY | 1.83E-13 |
| GO_SENSORY_PERCEPTION | 1.87E-13 |
| GO_REPRODUCTION | 1.92E-13 |
| GO_IMMUNE_RESPONSE | 2.09E-13 |
| GO_POSITIVE_REGULATION_OF_TRANSCRIPTION_FROM_RNA_POLYMERASE_II_PROMOTER | 2.12E-13 |
| GO_SYNAPTIC_SIGNALING | 2.30E-13 |
| GO_RNA_BINDING | 2.33E-13 |
| GO_PROTEOLYSIS | 4.98E-13 |
| REACTOME_GPCR_DOWNSTREAM_SIGNALING | 6.28E-13 |
| GO_CATION_CHANNEL_COMPLEX | 7.46E-13 |
| GO_POSITIVE_REGULATION_OF_IMMUNE_SYSTEM_PROCESS | 1.51E-12 |
| GO_SINGLE_ORGANISM_CELLULAR_LOCALIZATION | 1.61E-12 |
| GO_BEHAVIOR | 1.92E-12 |
| GO_REGULATION_OF_CELLULAR_COMPONENT_BIOGENESIS | 2.22E-12 |
| GO_AXON | 3.06E-12 |
| GO_RESPONSE_TO_ORGANIC_CYCLIC_COMPOUND | 3.11E-12 |
| GO_DEFENSE_RESPONSE | 3.14E-12 |
| GO_CIRCULATORY_SYSTEM_DEVELOPMENT | 3.31E-12 |
| GO_REGULATION_OF_CELL_ADHESION | 3.60E-12 |
| GO_NEGATIVE_REGULATION_OF_CELL_DIFFERENTIATION | 3.74E-12 |
| GO_SECRETION | 4.56E-12 |
| GO_PROTEIN_PHOSPHORYLATION | 5.36E-12 |
| GO_NEURON_PROJECTION_DEVELOPMENT | 5.65E-12 |
| GO_PERINUCLEAR_REGION_OF_CYTOPLASM | 5.69E-12 |
| GO_REGULATION_OF_SECRETION | 5.79E-12 |
| GO_CELLULAR_RESPONSE_TO_OXYGEN_CONTAINING_COMPOUND | 6.91E-12 |
| GO_REGULATION_OF_PROTEIN_LOCALIZATION | 7.59E-12 |
| GO_DENDRITE | 1.08E-11 |
| GO_SINGLE_ORGANISM_BIOSYNTHETIC_PROCESS | 1.09E-11 |
| GO_REGULATION_OF_SYSTEM_PROCESS | 1.33E-11 |
| GO_REGULATORY_REGION_NUCLEIC_ACID_BINDING | 1.56E-11 |
| GO_POSITIVE_REGULATION_OF_CELL_DEVELOPMENT | 1.59E-11 |
| GO_RECEPTOR_COMPLEX | 1.80E-11 |
| GO_POSITIVE_REGULATION_OF_NERVOUS_SYSTEM_DEVELOPMENT | 2.38E-11 |
| GO_REGULATION_OF_KINASE_ACTIVITY | 2.53E-11 |
| GO_CELL_MORPHOGENESIS_INVOLVED_IN_DIFFERENTIATION | 2.60E-11 |
| GO_SENSORY_ORGAN_DEVELOPMENT | 2.76E-11 |
| GO_POSTSYNAPSE | 3.21E-11 |
| GO_REGULATION_OF_IMMUNE_RESPONSE | 3.66E-11 |
| GO_CALCIUM_ION_TRANSPORT | 4.61E-11 |
| GO_POSITIVE_REGULATION_OF_CELL_PROJECTION_ORGANIZATION | 4.66E-11 |
| GO_DIVALENT_INORGANIC_CATION_TRANSPORT | 5.24E-11 |
| GO_CELLULAR_CHEMICAL_HOMEOSTASIS | 7.68E-11 |
| GO_ANCHORING_JUNCTION | 8.70E-11 |
| GO_ORGANOPHOSPHATE_METABOLIC_PROCESS | 9.15E-11 |
| GO_RESPONSE_TO_NITROGEN_COMPOUND | 1.11E-10 |
| GO_CELL_ACTIVATION | 1.24E-10 |
| GO_MULTICELLULAR_ORGANISMAL_SIGNALING | 1.28E-10 |
| GO_LIGAND_GATED_CHANNEL_ACTIVITY | 1.33E-10 |
| GO_REGULATION_OF_MEMBRANE_POTENTIAL | 1.34E-10 |
| GO_NEGATIVE_REGULATION_OF_CATALYTIC_ACTIVITY | 1.39E-10 |
| GO_CELLULAR_CATABOLIC_PROCESS | 1.41E-10 |
| GO_REGULATION_OF_METAL_ION_TRANSPORT | 1.49E-10 |
| REACTOME_NEURONAL_SYSTEM | 2.04E-10 |
| GO_SECRETION_BY_CELL | 2.15E-10 |
| GO_NEGATIVE_REGULATION_OF_TRANSCRIPTION_FROM_RNA_POLYMERASE_II_PROMOTER | 2.43E-10 |
| GO_CELL_PART_MORPHOGENESIS | 2.63E-10 |
| GO_SINGLE_ORGANISM_CATABOLIC_PROCESS | 2.76E-10 |
| GO_SYNAPTIC_MEMBRANE | 2.92E-10 |
| GO_PROTEINACEOUS_EXTRACELLULAR_MATRIX | 2.99E-10 |
| GO_GOLGI_MEMBRANE | 3.34E-10 |
| GO_EXTRACELLULAR_MATRIX | 3.53E-10 |
| GO_CALCIUM_ION_TRANSMEMBRANE_TRANSPORT | 3.74E-10 |
| GO_POSITIVE_REGULATION_OF_NEURON_DIFFERENTIATION | 4.59E-10 |
| GO_POSITIVE_REGULATION_OF_NEURON_PROJECTION_DEVELOPMENT | 4.84E-10 |
| GO_EMBRYO_DEVELOPMENT | 5.43E-10 |
| GO_IMMUNE_SYSTEM_DEVELOPMENT | 5.50E-10 |
| GO_REGULATION_OF_HEART_CONTRACTION | 6.02E-10 |
| GO_REGULATION_OF_BLOOD_CIRCULATION | 6.09E-10 |
| GO_OXIDATION_REDUCTION_PROCESS | 6.22E-10 |
| GO_CELL_MORPHOGENESIS_INVOLVED_IN_NEURON_DIFFERENTIATION | 7.24E-10 |
| GO_ZINC_ION_BINDING | 7.50E-10 |
| GO_REGULATION_OF_CELL_CYCLE | 8.19E-10 |
| GO_CALCIUM_ION_TRANSMEMBRANE_TRANSPORTER_ACTIVITY | 8.92E-10 |
| GO_PROTEIN_DOMAIN_SPECIFIC_BINDING | 9.15E-10 |
| GO_REGULATION_OF_VESICLE_MEDIATED_TRANSPORT | 9.68E-10 |
| GO_SENSORY_PERCEPTION_OF_MECHANICAL_STIMULUS | 1.06E-09 |
| GO_CELL_CYCLE | 1.31E-09 |
| GO_CYTOSKELETON_ORGANIZATION | 1.34E-09 |
| GO_DIVALENT_INORGANIC_CATION_TRANSMEMBRANE_TRANSPORTER_ACTIVITY | 1.40E-09 |
| GO_DOUBLE_STRANDED_DNA_BINDING | 2.38E-09 |
| GO_ENDOSOME | 2.42E-09 |
| GO_POSITIVE_REGULATION_OF_TRANSFERASE_ACTIVITY | 2.43E-09 |
| GO_NEURON_PROJECTION_MORPHOGENESIS | 3.08E-09 |
| GO_CELL_BODY | 3.39E-09 |
| GO_REGULATION_OF_GROWTH | 3.46E-09 |
| KEGG_NEUROACTIVE_LIGAND_RECEPTOR_INTERACTION | 3.67E-09 |
| GO_POTASSIUM_CHANNEL_COMPLEX | 4.35E-09 |
| GO_CIRCULATORY_SYSTEM_PROCESS | 5.69E-09 |
| GO_CELLULAR_LIPID_METABOLIC_PROCESS | 5.81E-09 |
| GO_HYDROLASE_ACTIVITY_ACTING_ON_ESTER_BONDS | 5.86E-09 |
| GO_PROTEIN_HOMODIMERIZATION_ACTIVITY | 6.74E-09 |
| GO_CELL_CELL_ADHESION_VIA_PLASMA_MEMBRANE_ADHESION_MOLECULES | 7.69E-09 |
| GO_FOREBRAIN_DEVELOPMENT | 8.22E-09 |
| GO_G_PROTEIN_COUPLED_RECEPTOR_ACTIVITY | 8.82E-09 |
| GO_ORGANIC_ACID_METABOLIC_PROCESS | 9.62E-09 |
| GO_ENZYME_ACTIVATOR_ACTIVITY | 1.02E-08 |
| GO_ACTIN_BINDING | 1.05E-08 |
| GO_TAXIS | 1.14E-08 |
| REACTOME_DEVELOPMENTAL_BIOLOGY | 1.15E-08 |
| GO_ION_HOMEOSTASIS | 1.16E-08 |
| GO_RESPONSE_TO_WOUNDING | 1.18E-08 |
| GO_REGULATION_OF_BODY_FLUID_LEVELS | 1.48E-08 |
| GO_POLY_A_RNA_BINDING | 1.51E-08 |
| GO_REGULATION_OF_ACTIN_FILAMENT_BASED_PROCESS | 1.56E-08 |
| GO_OXIDOREDUCTASE_ACTIVITY | 1.59E-08 |
| GO_CATALYTIC_COMPLEX | 2.01E-08 |
| GO_NUCLEAR_OUTER_MEMBRANE_ENDOPLASMIC_RETICULUM_MEMBRANE_NETWORK | 2.07E-08 |
| GO_HOMOPHILIC_CELL_ADHESION_VIA_PLASMA_MEMBRANE_ADHESION_MOLECULES | 2.12E-08 |
| GO_SKELETAL_SYSTEM_DEVELOPMENT | 2.13E-08 |
| GO_REGULATION_OF_CELL_MORPHOGENESIS_INVOLVED_IN_DIFFERENTIATION | 2.20E-08 |
| GO_REGULATION_OF_MAPK_CASCADE | 2.31E-08 |
| GO_TRANSMEMBRANE_RECEPTOR_PROTEIN_TYROSINE_KINASE_SIGNALING_PATHWAY | 2.63E-08 |
| GO_POSTSYNAPTIC_MEMBRANE | 2.79E-08 |
| GO_RESPONSE_TO_HORMONE | 2.91E-08 |
| GO_REGULATION_OF_ANATOMICAL_STRUCTURE_SIZE | 2.97E-08 |
| GO_PRESYNAPSE | 3.06E-08 |
| GO_MICROTUBULE_CYTOSKELETON | 3.72E-08 |
| GO_MUSCLE_SYSTEM_PROCESS | 3.74E-08 |
| GO_TELENCEPHALON_DEVELOPMENT | 4.12E-08 |
| REACTOME_TRANSMEMBRANE_TRANSPORT_OF_SMALL_MOLECULES | 4.46E-08 |
| GO_PROTEIN_KINASE_ACTIVITY | 5.03E-08 |
| GO_LOCOMOTORY_BEHAVIOR | 5.36E-08 |
| GO_ACTIN_FILAMENT_BASED_PROCESS | 5.86E-08 |
| GO_CARDIAC_CONDUCTION | 6.05E-08 |
| GO_REGULATION_OF_CATION_TRANSMEMBRANE_TRANSPORT | 6.06E-08 |
| GO_SENSORY_PERCEPTION_OF_CHEMICAL_STIMULUS | 6.41E-08 |
| GO_NEGATIVE_REGULATION_OF_CELLULAR_COMPONENT_ORGANIZATION | 6.63E-08 |
| GO_POSITIVE_REGULATION_OF_KINASE_ACTIVITY | 7.33E-08 |
| GO_RNA_POLYMERASE_II_TRANSCRIPTION_FACTOR_ACTIVITY_SEQUENCE_SPECIFIC_DNA_BINDING | 8.93E-08 |
| GO_ANION_TRANSPORT | 9.19E-08 |
| GO_ENDOCYTOSIS | 9.78E-08 |
| GO_ACTIN_CYTOSKELETON | 1.04E-07 |
| GO_HYDROLASE_ACTIVITY_ACTING_ON_ACID_ANHYDRIDES | 1.09E-07 |
| GO_NUCLEOSIDE_TRIPHOSPHATASE_REGULATOR_ACTIVITY | 1.14E-07 |
| GO_MUSCLE_CONTRACTION | 1.41E-07 |
| GO_APICAL_PART_OF_CELL | 1.43E-07 |
| GO_MODULATION_OF_SYNAPTIC_TRANSMISSION | 1.44E-07 |
| GO_KINASE_BINDING | 1.63E-07 |
| GO_WOUND_HEALING | 1.81E-07 |
| GO_HEMOSTASIS | 1.91E-07 |
| GO_NEURON_RECOGNITION | 2.10E-07 |
| GO_REGULATION_OF_DEFENSE_RESPONSE | 2.12E-07 |
| REACTOME_ION_CHANNEL_TRANSPORT | 2.16E-07 |
| GO_GUANYL_NUCLEOTIDE_EXCHANGE_FACTOR_ACTIVITY | 2.20E-07 |
| GO_NEURON_PROJECTION_GUIDANCE | 2.40E-07 |
| GO_SIDE_OF_MEMBRANE | 2.70E-07 |
| GO_RESPONSE_TO_CYTOKINE | 2.75E-07 |
| GO_CELLULAR_RESPONSE_TO_NITROGEN_COMPOUND | 2.87E-07 |
| GO_SINGLE_ORGANISM_BEHAVIOR | 2.91E-07 |
| GO_ORGANONITROGEN_COMPOUND_BIOSYNTHETIC_PROCESS | 2.93E-07 |
| GO_SINGLE_ORGANISM_CELL_ADHESION | 2.97E-07 |
| GO_ANCHORED_COMPONENT_OF_MEMBRANE | 2.99E-07 |
| GO_TRANSCRIPTION_FROM_RNA_POLYMERASE_II_PROMOTER | 3.19E-07 |
| GO_CELL_LEADING_EDGE | 3.40E-07 |
| GO_REGULATION_OF_HORMONE_LEVELS | 3.56E-07 |
| GO_NEGATIVE_REGULATION_OF_PROTEIN_MODIFICATION_PROCESS | 3.76E-07 |
| GO_SECRETORY_VESICLE | 4.02E-07 |
| GO_CARBOHYDRATE_DERIVATIVE_BIOSYNTHETIC_PROCESS | 4.29E-07 |
| GO_REGULATION_OF_CELLULAR_COMPONENT_SIZE | 4.83E-07 |
| GO_LIPID_BINDING | 5.23E-07 |
| GO_MULTICELLULAR_ORGANISM_REPRODUCTION | 5.27E-07 |
| GO_VOLTAGE_GATED_ION_CHANNEL_ACTIVITY | 5.35E-07 |
| GO_NEGATIVE_REGULATION_OF_CELL_PROLIFERATION | 6.40E-07 |
| GO_ACTIN_BASED_CELL_PROJECTION | 7.03E-07 |
| KEGG_CALCIUM_SIGNALING_PATHWAY | 7.23E-07 |
| GO_EPITHELIAL_CELL_DIFFERENTIATION | 7.25E-07 |
| GO_RESPONSE_TO_LIPID | 7.80E-07 |
| GO_GROWTH | 8.00E-07 |
| GO_POSITIVE_REGULATION_OF_ION_TRANSPORT | 8.01E-07 |
| GO_DIVALENT_INORGANIC_CATION_HOMEOSTASIS | 8.05E-07 |
| GO_LEUKOCYTE_ACTIVATION | 8.06E-07 |
| GO_REGULATION_OF_CYTOKINE_PRODUCTION | 8.45E-07 |
| GO_NEUROTRANSMITTER_TRANSPORT | 9.04E-07 |
| GO_RESPONSE_TO_BIOTIC_STIMULUS | 1.05E-06 |
| GO_MONOVALENT_INORGANIC_CATION_TRANSPORT | 1.05E-06 |
| GO_PEPTIDASE_ACTIVITY | 1.05E-06 |
| GO_REGULATION_OF_PROTEOLYSIS | 1.06E-06 |
| GO_HINDBRAIN_DEVELOPMENT | 1.09E-06 |
| REACTOME_HEMOSTASIS | 1.10E-06 |
| GO_NEGATIVE_REGULATION_OF_PHOSPHORUS_METABOLIC_PROCESS | 1.23E-06 |
| GO_CELL_RECOGNITION | 1.24E-06 |
| GO_EXTRACELLULAR_LIGAND_GATED_ION_CHANNEL_ACTIVITY | 1.25E-06 |
| GO_REGULATION_OF_HOMEOSTATIC_PROCESS | 1.27E-06 |
| GO_HEART_PROCESS | 1.44E-06 |
| GO_CYTOPLASMIC_VESICLE_PART | 1.48E-06 |
| GO_VASCULATURE_DEVELOPMENT | 1.51E-06 |
| GO_PRESYNAPTIC_PROCESS_INVOLVED_IN_SYNAPTIC_TRANSMISSION | 1.89E-06 |
| REACTOME_IMMUNE_SYSTEM | 1.98E-06 |
| REACTOME_TRANSMISSION_ACROSS_CHEMICAL_SYNAPSES | 2.03E-06 |
| GO_CELL_CELL_JUNCTION | 2.04E-06 |
| GO_APICAL_PLASMA_MEMBRANE | 2.36E-06 |
| GO_NEGATIVE_REGULATION_OF_CELL_DEVELOPMENT | 2.43E-06 |
| GO_MULTI_ORGANISM_REPRODUCTIVE_PROCESS | 2.49E-06 |
| GO_REGULATION_OF_INTRACELLULAR_TRANSPORT | 2.65E-06 |
| GO_POSITIVE_REGULATION_OF_CELL_DEATH | 2.76E-06 |
| GO_POSITIVE_REGULATION_OF_IMMUNE_RESPONSE | 2.77E-06 |
| GO_MONOVALENT_INORGANIC_CATION_TRANSMEMBRANE_TRANSPORTER_ACTIVITY | 2.89E-06 |
| GO_DEVELOPMENTAL_PROCESS_INVOLVED_IN_REPRODUCTION | 2.90E-06 |
| GO_SYNAPSE_ORGANIZATION | 3.36E-06 |
| GO_REGULATION_OF_CALCIUM_ION_TRANSPORT | 3.50E-06 |
| GO_EXTRACELLULAR_STRUCTURE_ORGANIZATION | 3.54E-06 |
| REACTOME_GABA_A_RECEPTOR_ACTIVATION | 3.65E-06 |
| GO_DEVELOPMENTAL_GROWTH | 3.86E-06 |
| GO_REGULATION_OF_CELL_ACTIVATION | 4.26E-06 |
| GO_POSITIVE_REGULATION_OF_LOCOMOTION | 4.41E-06 |
| GO_RESPONSE_TO_OXYGEN_LEVELS | 5.13E-06 |
| GO_CELL_PROJECTION_MEMBRANE | 5.77E-06 |
| GO_SULFUR_COMPOUND_BINDING | 5.93E-06 |
| GO_REGULATION_OF_SEQUESTERING_OF_CALCIUM_ION | 5.95E-06 |
| GO_CARDIAC_MUSCLE_CELL_CONTRACTION | 6.08E-06 |
| GO_TUBE_DEVELOPMENT | 6.25E-06 |
| REACTOME_CELL_CELL_COMMUNICATION | 6.68E-06 |
| REACTOME_AXON_GUIDANCE | 6.73E-06 |
| GO_VOCALIZATION_BEHAVIOR | 6.74E-06 |
| GO_TRANSMEMBRANE_RECEPTOR_PROTEIN_PHOSPHATASE_ACTIVITY | 6.89E-06 |
| GO_RAS_GUANYL_NUCLEOTIDE_EXCHANGE_FACTOR_ACTIVITY | 6.93E-06 |
| GO_GABA_RECEPTOR_COMPLEX | 7.36E-06 |
| GO_ACTIN_FILAMENT_BASED_MOVEMENT | 7.84E-06 |
| GO_POSITIVE_REGULATION_OF_MAPK_CASCADE | 7.88E-06 |
| GO_POSITIVE_REGULATION_OF_CELLULAR_COMPONENT_BIOGENESIS | 8.14E-06 |
| GO_PROTEIN_OLIGOMERIZATION | 8.24E-06 |
| GO_REGULATION_OF_CYTOSKELETON_ORGANIZATION | 8.70E-06 |
| GO_ANION_TRANSMEMBRANE_TRANSPORTER_ACTIVITY | 8.71E-06 |
| GO_EXTERNAL_SIDE_OF_PLASMA_MEMBRANE | 8.77E-06 |
| GO_ION_CHANNEL_BINDING | 9.82E-06 |
| GO_ACTIVATION_OF_IMMUNE_RESPONSE | 9.92E-06 |
| GO_REGULATION_OF_CYTOPLASMIC_TRANSPORT | 1.06E-05 |
| GO_REGULATION_OF_HEART_RATE | 1.07E-05 |
| GO_REGULATION_OF_DENDRITE_DEVELOPMENT | 1.09E-05 |
| GO_REGULATION_OF_HEART_RATE_BY_CARDIAC_CONDUCTION | 1.15E-05 |
| GO_NEGATIVE_REGULATION_OF_HYDROLASE_ACTIVITY | 1.19E-05 |
| GO_SYNAPSE_ASSEMBLY | 1.19E-05 |
| GO_CELL_PROLIFERATION | 1.22E-05 |
| GO_SEXUAL_REPRODUCTION | 1.23E-05 |
| GO_LYTIC_VACUOLE | 1.27E-05 |
| GO_SERINE_HYDROLASE_ACTIVITY | 1.28E-05 |
| GO_NEGATIVE_REGULATION_OF_CELL_ADHESION | 1.30E-05 |
| GO_SIGNAL_RELEASE | 1.32E-05 |
| GO_POSITIVE_REGULATION_OF_CELL_ADHESION | 1.32E-05 |
| GO_ACTIN_MEDIATED_CELL_CONTRACTION | 1.34E-05 |
| GO_VOLTAGE_GATED_CATION_CHANNEL_ACTIVITY | 1.37E-05 |
| GO_REGULATION_OF_SYNAPSE_STRUCTURE_OR_ACTIVITY | 1.37E-05 |
| GO_IMMUNE_EFFECTOR_PROCESS | 1.39E-05 |
| GO_PHOSPHOLIPID_BINDING | 1.39E-05 |
| GO_DETECTION_OF_STIMULUS | 1.45E-05 |
| GO_DENDRITE_DEVELOPMENT | 1.53E-05 |
| GO_STRUCTURAL_MOLECULE_ACTIVITY | 1.54E-05 |
| GO_PEPTIDYL_AMINO_ACID_MODIFICATION | 1.57E-05 |
| KEGG_AXON_GUIDANCE | 1.59E-05 |
| GO_CELLULAR_RESPONSE_TO_CYTOKINE_STIMULUS | 1.63E-05 |
| GO_CELL_SUBSTRATE_JUNCTION | 1.70E-05 |
| GO_REGULATION_OF_CALCIUM_ION_IMPORT | 1.74E-05 |
| REACTOME_LIGAND_GATED_ION_CHANNEL_TRANSPORT | 1.78E-05 |
| GO_EAR_DEVELOPMENT | 1.92E-05 |
| KEGG_VASCULAR_SMOOTH_MUSCLE_CONTRACTION | 2.00E-05 |
| GO_RESPONSE_TO_GROWTH_FACTOR | 2.01E-05 |
| GO_EYE_DEVELOPMENT | 2.05E-05 |
| GO_POSITIVE_REGULATION_OF_CELL_MORPHOGENESIS_INVOLVED_IN_DIFFERENTIATION | 2.13E-05 |
| GO_CELL_CYCLE_PROCESS | 2.14E-05 |
| GO_ADULT_BEHAVIOR | 2.15E-05 |
| GO_NITROGEN_COMPOUND_TRANSPORT | 2.19E-05 |
| GO_NEURON_CELL_CELL_ADHESION | 2.28E-05 |
| GO_GABA_RECEPTOR_ACTIVITY | 2.33E-05 |
| GO_GLYCOSAMINOGLYCAN_BINDING | 2.38E-05 |
| GO_GLAND_DEVELOPMENT | 2.43E-05 |
| REACTOME_NEUROTRANSMITTER_RECEPTOR_BINDING_AND_DOWNSTREAM_TRANSMISSION_IN_THE_POSTSYNAPTIC_CELL | 2.48E-05 |
| GO_REGULATION_OF_POTASSIUM_ION_TRANSMEMBRANE_TRANSPORT | 2.59E-05 |
| GO_CELL_CELL_SIGNALING_INVOLVED_IN_CARDIAC_CONDUCTION | 2.60E-05 |
| GO_ACTION_POTENTIAL | 2.72E-05 |
| GO_NEGATIVE_REGULATION_OF_LOCOMOTION | 2.78E-05 |
| GO_EMBRYO_DEVELOPMENT_ENDING_IN_BIRTH_OR_EGG_HATCHING | 2.94E-05 |
| GO_REGULATION_OF_MULTI_ORGANISM_PROCESS | 2.96E-05 |
| GO_REGULATION_OF_RELEASE_OF_SEQUESTERED_CALCIUM_ION_INTO_CYTOSOL_BY_SARCOPLASMIC_RETICULUM | 2.96E-05 |
| GO_REGULATION_OF_TRANSPORTER_ACTIVITY | 2.98E-05 |
| GO_REGULATION_OF_CARDIAC_MUSCLE_CONTRACTION | 3.05E-05 |
| GO_REGULATION_OF_CELL_GROWTH | 3.06E-05 |
| GO_VACUOLAR_PART | 3.14E-05 |
| GO_RESPONSE_TO_INORGANIC_SUBSTANCE | 3.17E-05 |
| GO_NEUROMUSCULAR_PROCESS | 3.18E-05 |
| GO_CYTOSOLIC_CALCIUM_ION_TRANSPORT | 3.23E-05 |
| GO_REGULATION_OF_SYSTEMIC_ARTERIAL_BLOOD_PRESSURE | 3.27E-05 |
| GO_REGULATION_OF_RELEASE_OF_SEQUESTERED_CALCIUM_ION_INTO_CYTOSOL | 3.47E-05 |
| GO_POSITIVE_REGULATION_OF_ORGANELLE_ORGANIZATION | 3.66E-05 |
| GO_AGING | 3.68E-05 |
| GO_GLYCOPROTEIN_METABOLIC_PROCESS | 3.78E-05 |
| GO_COGNITION | 3.91E-05 |
| GO_ORGANELLE_LOCALIZATION | 4.10E-05 |
| GO_EXOCYTOSIS | 4.25E-05 |
| GO_REGULATION_OF_WNT_SIGNALING_PATHWAY | 4.61E-05 |
| GO_LOCALIZATION_WITHIN_MEMBRANE | 4.85E-05 |
| GO_REGULATION_OF_CATABOLIC_PROCESS | 4.91E-05 |
| GO_MEMBRANE_MICRODOMAIN | 4.97E-05 |
| GO_REGULATION_OF_CARDIAC_CONDUCTION | 5.01E-05 |
| GO_REGULATION_OF_NEUROTRANSMITTER_LEVELS | 5.05E-05 |
| GO_NEGATIVE_REGULATION_OF_NERVOUS_SYSTEM_DEVELOPMENT | 5.21E-05 |
| GO_SECRETORY_GRANULE | 5.48E-05 |
| GO_CELL_COMMUNICATION_INVOLVED_IN_CARDIAC_CONDUCTION | 5.48E-05 |
| GO_ANION_TRANSMEMBRANE_TRANSPORT | 5.54E-05 |
| GO_CARDIAC_MUSCLE_CELL_ACTION_POTENTIAL | 5.92E-05 |
| GO_PHOSPHORIC_ESTER_HYDROLASE_ACTIVITY | 6.12E-05 |
| GO_SCAFFOLD_PROTEIN_BINDING | 6.16E-05 |
| GO_REGULATION_OF_PROTEIN_SERINE_THREONINE_KINASE_ACTIVITY | 6.46E-05 |
| GO_PROTEIN_SERINE_THREONINE_KINASE_ACTIVITY | 6.47E-05 |
| GO_VESICLE_MEMBRANE | 6.47E-05 |
| GO_CELL_ADHESION_MOLECULE_BINDING | 6.49E-05 |
| GO_CELLULAR_RESPONSE_TO_ORGANIC_CYCLIC_COMPOUND | 6.90E-05 |
| GO_POSITIVE_REGULATION_OF_ENDOTHELIAL_CELL_PROLIFERATION | 6.91E-05 |
| GO_CALMODULIN_BINDING | 7.01E-05 |
| GO_INNERVATION | 7.33E-05 |
| GO_REGULATION_OF_STRIATED_MUSCLE_CONTRACTION | 8.05E-05 |
| GO_NEURON_MIGRATION | 8.20E-05 |
| GO_NERVE_DEVELOPMENT | 8.53E-05 |
| GO_SECOND_MESSENGER_MEDIATED_SIGNALING | 8.54E-05 |
| GO_PLATELET_ACTIVATION | 8.81E-05 |
| GO_POTASSIUM_ION_TRANSPORT | 8.89E-05 |
| GO_LYMPHOCYTE_ACTIVATION | 9.22E-05 |
| GO_POTASSIUM_CHANNEL_ACTIVITY | 9.23E-05 |
| GO_VENTRICULAR_CARDIAC_MUSCLE_CELL_ACTION_POTENTIAL | 9.29E-05 |
| GO_ORGANIC_ANION_TRANSPORT | 9.40E-05 |
| GO_CEREBELLAR_CORTEX_DEVELOPMENT | 9.48E-05 |
| GO_HINDBRAIN_MORPHOGENESIS | 9.62E-05 |
